# Low systolic blood pressure increases risk of allergic rhinitis: Evidence from a bidirectional Mendelian randomization study accounting for smoking environment

**DOI:** 10.18332/tid/220356

**Published:** 2026-06-19

**Authors:** Zhu Mao, Qingqing Xu, Yuting Huo, Yunliang Liu

**Affiliations:** 1 Department of Otolaryngology, Fujian Children’s Hospital (Fujian Branch of Shanghai Children’s Medical Center), College of Clinical Medicine for Obstetrics & Gynecology and Pediatrics, Fujian Medical University, Fuzhou, People's Republic of China; 2 Department of Otolaryngology, Fujian Maternity and Child Health Hospital, College of Clinical Medicine for Obstetrics & Gynecology and Pediatrics, Fujian Medical University, Fuzhou, People's Republic of China

**Keywords:** systolic blood pressure, smoking environment, allergic rhinitis, Mendelian randomization

## Abstract

**INTRODUCTION:**

Allergic rhinitis (AR) is one of the most common diseases in adults. Dust and secondhand smoke in the air can both trigger AR and elevated blood pressure. The objective of this study was to assess the impact of systolic blood pressure (SBP) on the risk of AR using a bidirectional two-sample Mendelian randomization (MR) framework.

**METHODS:**

Summary-level genome-wide association study data for SBP and AR were retrieved from the MR-Base platform. Single-nucleotide polymorphisms (SNPs) associated with SBP as exposure at genome-wide significance (p<5×10^-8^) and exhibiting low linkage disequilibrium (r^2^<0.001) were selected as instrumental variables. Bidirectional two-sample MR analyses were performed using the inverse-variance weighted method (IVW), weighted median estimator, and MR-Egger regression to estimate associations between both directions.

**RESULTS:**

A total of 421 independent SNPs associated with SBP were included in the forward MR analysis. All three methods consistently indicated that genetically lower SBP is associated with an increased risk of AR (IVW odds ratio, OR=0.9997; 95% CI: 0.9995–0.9999; weighted median OR=0.9998; 95% CI: 0.9995–1.0001; MR-Egger OR=0.9996; 95% CI: 0.9990–1.0001). Reverse MR analysis provided no evidence that genetic liability to AR influences SBP.

**CONCLUSIONS:**

These findings provide genetic evidence for a relationship in which lower systolic blood pressure increases susceptibility to allergic rhinitis.

## INTRODUCTION

Tobacco use and exposure to secondhand tobacco smoke (SHS) are well-established carcinogenic factors and represent major public health concerns. Tobacco consumption remains the single largest preventable health risk and the leading cause of premature mortality in the European Union (EU), accounting for nearly 700000 deaths annually^1^. Observational studies conducted in several Asian countries among both adult and pediatric populations have reported a significant association between tobacco exposure and the development of allergic rhinitis (AR)^2,3^. Furthermore, a large population-based study in China demonstrated that children exposed to passive smoking have an increased risk of developing AR and asthma^4^.

Accumulating epidemiological evidence has consistently shown that tobacco exposure adversely affects blood pressure regulation and cardiovascular prognosis, establishing it as an important modifiable risk factor for cardiovascular disease^5^. Moreover, worsening smoking behaviors have been associated with an increased risk of cardiovascular events and all-cause mortality^6^. A cross-cohort collaborative study involving 182364 participants reported that smoking-induced subclinical vascular damage, including elevated blood pressure and increased arterial stiffness, can be partially reversed within five years after smoking cessation; however, these measures do not fully return to the physiological levels observed in never smokers^7^.

Although the pathophysiology of hypertension is multifactorial and not yet fully elucidated, emerging evidence suggests a complex interplay between hypertension and AR^8^. However, the temporal divergence in the onset of AR and CVD may complicate this association^9^. AR prevalence typically peaks during the second to fourth decades of life and subsequently declines, whereas the incidence of CVD increases with advancing age^10^ . This temporal mismatch raises the question of whether lower systolic blood pressure (SBP) could be a risk factor for AR. Contrary to this hypothesis, Kony et al.^11^ reported that, after adjusting for confounding variables including age, body mass index (BMI), hypercholesterolemia, and smoking status, men with AR exhibited significantly higher SBP compared to their non-AR counterparts (130 ± 12.7 vs 123.5 ± 13.9 mmHg; p=0.002), suggesting that AR may serve as an independent risk factor for elevated SBP in males^11^. Conversely, a 2018 study by Sakallioglu et al.^12^ found no significant differences in systolic or diastolic blood pressure among patients with seasonal or perennial AR. Their analysis concluded that there was no statistically significant association between AR and blood pressure elevation^12^.

Establishing a relationship between low SBP and AR poses significant challenges in observational studies due to inherent methodological limitations, including the absence of randomization, potential confounding factors, and the risk of reverse causality. Although randomized controlled trials (RCTs) are considered the gold standard for causal inference, ethical and logistical constraints often limit their feasibility in this context. Mendelian randomization (MR) has emerged as a robust alternative approach, leveraging genetic variants as instrumental variables to infer associations between exposures and outcomes. These genetic variants, which are randomly allocated at conception in accordance with Mendel’s laws, provide a natural experiment that minimizes confounding and mitigates reverse causality^13^. The rapid advancement of genome-wide association studies (GWAS) and meta-analyses of GWAS data has further enhanced the validity and applicability of MR as a powerful tool for causal inference in epidemiology^14^.

Two-sample MR, which utilizes summary-level statistics from independent GWAS datasets, enables the estimation of the effects by linking genetic variants associated with an exposure in one sample to those associated with an outcome in a separate, but comparable, sample. This design improves statistical power and generalizability while preserving the foundational assumptions of MR. Recent MR studies investigating the association between AR and hypertension have suggested a potential protective effect of AR against the development of hypertension in European populations^15^. Specifically, these findings indicate that AR may be linked to a reduced risk of hypertension. However, previous analyses were limited by the small number of single-nucleotide polymorphisms (SNPs) used as instrumental variables and the reliance on outdated genetic datasets, which may compromise the robustness and generalizability of the results. In the present study, we sought to address the aforementioned gap by performing a bidirectional two-sample Mendelian randomization (MR) analysis to elucidate the relationship between systolic blood pressure (SBP) and allergic rhinitis (AR), with active or passive smoking incorporated as a key moderating variable. We utilized the most up-to-date GWAS summary statistics for SBP (ieu-b-38), AR (ebi-a-GCST90038664), and current tobacco smoking (ukb-b-223). By integrating smoking-related genetic instruments into our analysis, we aimed to determine whether the association between SBP and AR is independent of active or passive smoking, or whether smoking mediates or modifies this relationship.

## METHODS

### Data source

**Figure 1:**
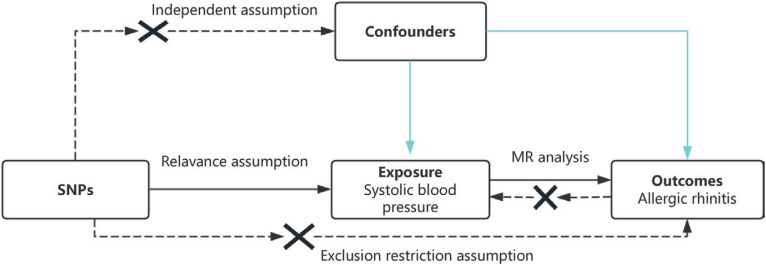
Diagram of the MR models assessing if low systolic blood pressure increases the risk of allergic rhinitis, accounting for smoking

**Figure 2:**
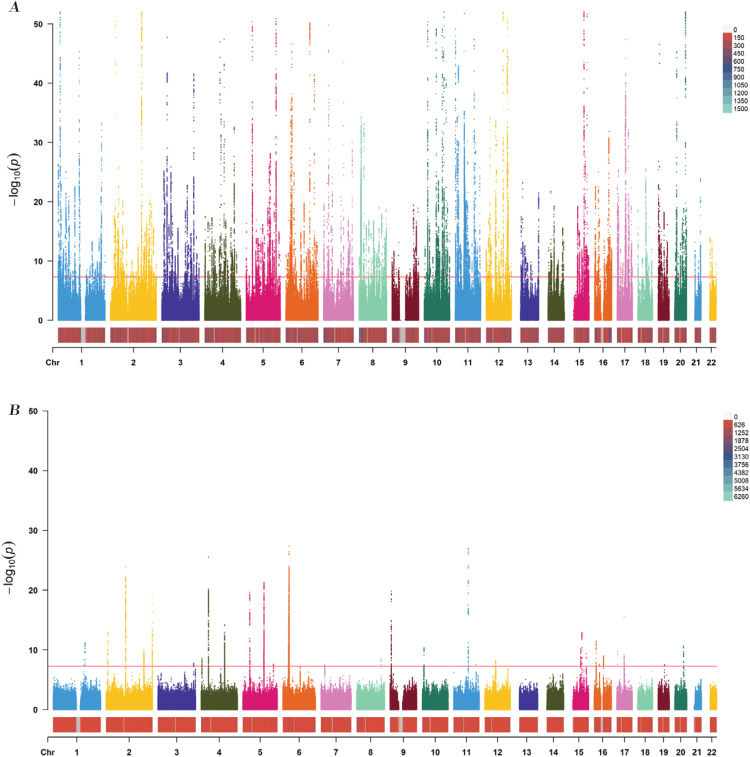
Manhattan plot of the included single nucleotide polymorphisms: A) SBP, data from IEU OpenGWAS in 2018 (SNPs=7088083; red line as threshold; p=5×10^-8^); B) AR data from IEU OpenGWAS in 2021 (SNPs=9587836; red line as threshold; p=5×10^-8^)

This study was conducted in strict accordance with the core design principles and analytical framework of MR, as outlined in Figure 1. Summary-level GWAS data for SBP were obtained from the International Consortium of Blood Pressure (ieu-b-38), published in 2018 and accessible via the IEU Open GWAS Project^16^. This dataset comprises 757601 individuals and includes 7088083 SNPs (Figure 2A).

The GWAS summary data for AR were sourced from the EMBL-EBI database (study ID: ebi-a-GCST90038664), based on a European cohort published in 2021^17^. This dataset includes 484598 participants and 9587836 SNPs (Figure 2B). Detailed characteristics of the included studies and datasets are summarized in Table 1. The smoking dataset was obtained from the UK Biobank^18^ and included 462434 participants and 9851867 SNPs. The data were published in 2018.

**Table 1:** Details of studies and datasets used in the study, data from 2018 and 2021 (N=757601 for systolic blood pressure; N=484598 for allergic rhinitis; N=462434 for current tobacco smoking)

*Exposure/outcomes*	*Web source*	*Sample size*	*SNP size*	*Authors*	*Consortium*	*Year*	*Population studied*
**Systolic blood pressure**	IEU Open GWAS project (ieu-b-38)	757601	7088083	Evangelou et al.^16^	International Consortium of Blood Pressure	2018	European
**Allergic rhinitis**	EMBL-EBI (ebi-a-GCST90038664)	484598	9587836	NA	NA	2021	European
**Current tobacco smoking**	UK Biobank (ukb-b-223)	462434	9851867	Hemani et al.^20^	MRC-IEU	2018	European

All MR analyses were conducted in accordance with the STROBE-MR reporting guidelines to ensure methodological rigor and transparency (Supplementary file Table 1)^19^.

### Selection of instrumental variables

Instrumental variables (IVs) were rigorously selected based on strict inclusion criteria to ensure the validity of the MR analysis. SNPs were required to: 1) reach genome-wide significance (p<5×10^-8^) for association with the exposure; and 2) exhibit a minor allele frequency (MAF) greater than 0.01 in the outcome dataset. To minimize linkage disequilibrium (LD), SNPs with pairwise r^2^ >0.001 within a 10000 kb window were excluded. Additionally, SNPs identified as associated with potential confounders or outcomes via the *FastTraitR* package were excluded to mitigate horizontal pleiotropy. Specifically, variants such as rs6771917, rs2643826, rs3918226, rs3735533, rs12978472, rs12258967, rs57866767, rs6271, rs6031431, rs17608766, rs7463212, rs12509595, rs35443, rs2024385, rs7134677, rs7338758, rs12906962, rs77924615, rs35783704, rs8118848 – implicated in medication use (calcium channel blockers) – were manually removed from the analysis, several SNPs (rs2014408, rs17080102, rs3918226, rs17249754, rs17010957, rs13107325, rs3918226) were excluded due to their known associations with tobacco use. After applying these filters, a total of 421 independent SNPs were retained for downstream analysis.

The proportion of variance explained by each SNP was calculated using the formula:

R^2^ = [2β^2^×EAF×(1−EAF)]/[2β^2^×EAF×(1−EAF) + 2SE^2^×N×EAF×(1−EAF)]

where β is the effect size, EAF is the effect allele frequency, SE is the standard error, and N is the sample size. Instrument strength was evaluated using the F-statistic:

F = [(N−k−1)/k]×[R^2^/(1 − R^2^)]

where k denotes the number of IVs, an F-statistic <10 was considered indicative of weak instruments, which may bias the estimates^20^.

### Main analysis

Following the extraction of summary-level GWAS data for SBP and AR via the MR-Base platform, a two-sample MR analysis was performed using the *TwoSampleMR* package (v0.6.14) in R (v4.3.2). Three complementary MR methods were applied to estimate the effects: inverse variance weighted (IVW), weighted median, and MR-Egger regression^20-23^. The IVW method, which assumes all instruments are valid, combines the SNP-specific Wald ratios using a meta-analytic approach^20,21^. The weighted median approach provides consistent estimates when at least 50% of the weight comes from valid instruments. MR-Egger regression, which accounts for directional pleiotropy, produces unbiased estimates even if some instruments are invalid, provided the InSIDE assumption is met^23^. The slope of the MR-Egger regression represents the estimate, while the intercept term tests for the presence of unbalanced horizontal pleiotropy. Results are reported as odds ratio (OR) with corresponding 95% confidence interval (CI), and a p<0.05 was considered statistically significant.

### Sensitivity analysis

To assess the robustness of the findings, leave-one-out analysis was conducted. Each SNP was sequentially removed, and the estimate was recalculated using the IVW method to evaluate the influence of individual SNPs on the overall results. This approach ensures that the observed associations are not disproportionately driven by any single instrumental variable^24^.

## RESULTS

### Characteristics of included SNPs

Comprehensive information on the instrumental variables used in this study is provided in Supplementary file Table 2. This includes the effect allele (EA), effect allele frequency (EAF), and detailed association estimates with both SBP and AR, including β coefficients, standard errors (SE), and corresponding p-values.

### The effect of SBP on AR

**Figure 3:**
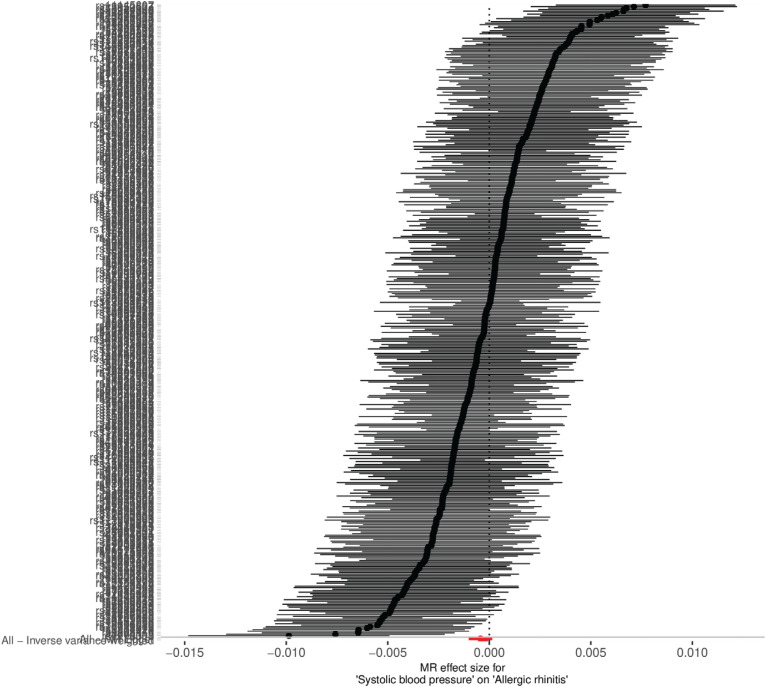
Forest plot of single-nucleotide polymorphisms (SNPs) associated with SBP and the risk of AR. Black points represent the log odds ratio (OR) for systolic blood pressure per standard deviation (SD) decrease in allergic rhinitis, data from 2018 and 2021 (N=7088083 for SBP; N=9587836 for AR)

**Figure 4:**
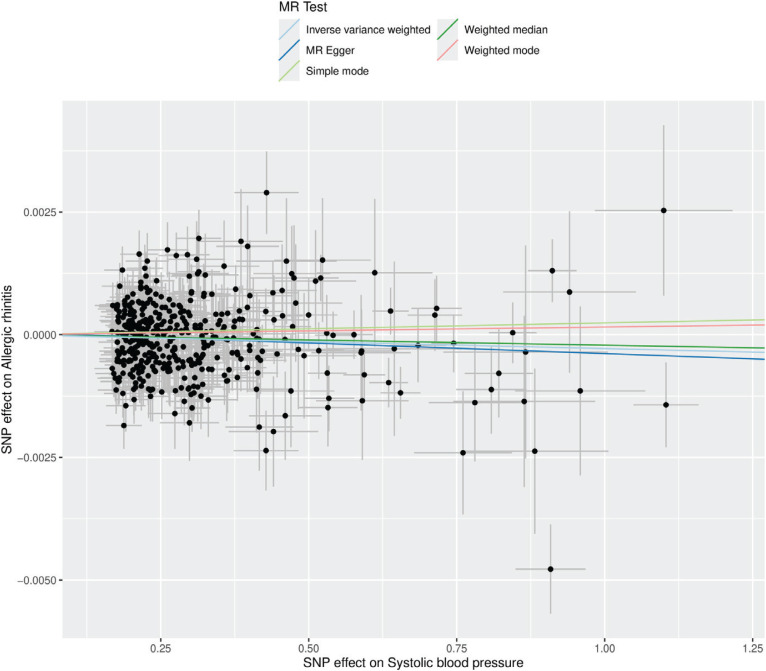
Scatter plot of SNP effect estimates for systolic blood pressure on the x-axis (in standard deviation units) versus allergic rhinitis on the y-axis (log odds ratio), including 95% CI. The regression slopes correspond to estimates from inverse-variance weighted (IVW), weighted median, and MR-Egger analyses. IEU OpenGWAS 2018 and 2021 (N=1242119)

The effect of SBP on the risk of AR was evaluated using multiple MR methods. As shown in Table 2, the IVW method indicated a statistically significant inverse association, suggesting that lower genetically predicted SBP is associated with an increased risk of AR (OR=0.9997; 95% CI: 0.9995–0.9999). This trend was corroborated by the weighted median estimator (OR=0.9998; 95% CI: 0.9995–1.0001) and MR-Egger regression (OR=0.9996; 95% CI: 0.9990–1.0001), though the latter did not reach statistical significance. These findings are visually represented in the forest plot (Figure 3) and scatter plot (Figure 4).

**Table 2:** Associations between genetically determined MR analysis of exposures with outcomes, IEU OpenGWAS 2018 and 2021 (N=1242119)

*Exposure*	*Outcome*	*The forward MR*	*β*	*SE*	*OR (95% CI)*	*p*
ieu-b-38	ebi-a-GCST90038664	MR Egger	-0.0004	0.0005	0.9996 (0.9990–1.0001)	0.145
		Weighted median	-0.0002	0.0002	0.9998 (0.9995–1.0001)	0.160
		IVW	-0.0003	0.0001	0.9997 (0.9995–0.9999)	0.011
		Simple mode	0.0002	0.0005	1.0000 (0.9992–1.0013)	0.645
		Weighted mode	0.0003	0.0004	1.0000 (0.9994–1.0010)	0.702
***Exposure***	***Outcome***	***The reverse MR***	***β***	***SE***	***OR (95% CI)***	***p***
ebi-a-GCST90038664	ieu-b-38	MR Egger	22.144	10.339	0.000 (6.548–26.166)	0.041
		Weighted median	0.304	2.991	1.356 (0.004–476.724)	0.919
		IVW	2.124	3.162	8.365 (0.01–4113.977)	0.502
		Simple mode	-3.845	6.099	0.021 (1.38E-07–3324.778)	0.534
		Weighted mode	-3.084	5.816	0.046 (5.12E-07–4089.663)	0.600

IVW: inverse variance weighted. SE: standard error.

### Sensitivity analysis

Assessment of directional pleiotropy using MR-Egger regression revealed no significant evidence of bias (intercept=0.000047, SE=0.000087, p=0.587), as shown in Table 3. Although Cochran’s Q test indicated the presence of heterogeneity among the instrumental variables in both the IVW and MR-Egger analyses, the overall estimates remained stable. Furthermore, leave-one-out sensitivity analysis demonstrated that no single SNP exerted a disproportionate influence on the estimate, thereby affirming the robustness of the findings (Supplementary file Figure 1).

**Table 3:** Heterogeneity and pleiotropy analyses, IEU OpenGWAS 2018 and 2021 (N=1242119)

*Analyses*	*Exposure*	*Outcome*	*Heterogeneity test*			*Pleiotropy test*	
			*IVW Q*	*p*	*MR-Egger Q*	*p*	*MR-Egger / PRESSO p*
**Forward MR analyses**	ieu-b-38	ebi-a-GCST90038664	653.429	0.000	652.969	0.000	0.587
**Reverse MR analyses**	ebi-a-GCST90038664	ieu-b-38	98.709	0.000	85.711	0.000	0.053

IVW: inverse variance weighted. MR: Mendelian randomization.

### Reverse mendelian randomization analysis

To explore the potential reverse association between AR and SBP, a bidirectional MR analysis was conducted. After removing the SNP rs6771917, which was associated with the confounding trait ‘heart function’, a total of 29 independent SNPs significantly associated with AR (p<5×10^-8^) were included in the reverse analysis. As shown in Table 2, the IVW method revealed no evidence of an effect of genetically predicted AR on SBP. Similarly, MR-Egger and IVW-based Cochran’s Q statistics (Table 3) indicated no significant heterogeneity among the selected SNPs. The MR-PRESSO global test detected no statistically significant pleiotropy (p=0.053). Leave-one-out analysis further confirmed the robustness of the results, showing that exclusion of any individual SNP did not materially alter the outcome (Supplementary file Figure 2).

### Additional mendelian randomization analysis

To determine whether the association between systolic blood pressure (SBP) and AR is independent of active or passive smoking, or whether smoking mediates or modifies this relationship, we conducted additional Mendelian randomization analyses to assess the causal effects of smoking on SBP and on AR. However, no evidence of a relationship was identified between smoking and SBP or between smoking and AR. (Supplementary file Table 3).

## DISCUSSION

Tobacco control is a global public health priority, and passive smoking remains a major environmental health hazard, particularly in indoor environments and among vulnerable populations such as children, pregnant women, and non-smoking adults^25^. Tobacco smoke contains thousands of chemicals, including nicotine, tar, and formaldehyde. Their exposure induces oxidative stress and promotes the release of pro-inflammatory cytokines (e.g. IL-8, IL-6, IL-17, TNF-α), resulting in nasal mucosal injury, increased epithelial permeability, and excessive mucus secretion^26^. In allergic rhinitis (AR), secondhand smoke exposure is associated with reduced expression of tight junction (TJ) molecules, contributing to epithelial barrier disruption^27^. Moreover, secondhand smoke impairs ciliary motility, limiting clearance of secretions and foreign particles, thereby promoting persistent inflammation and chronic rhinitis symptoms such as nasal obstruction and purulent discharge^28^. Beyond these respiratory damages, the systemic inflammatory response triggered by secondhand smoke also exerts adverse effects on cardiovascular function, as active and passive smoking have been associated with subtle but significant increases in SBP among adolescents and adults^29^.

Lee et al.^30^ reported an inverse association between AR and diabetes mellitus, suggesting potential heterogeneity in allergic disease–metabolic disease interactions. In a large-scale, population-based study involving 30590 Korean participants, the prevalence of AR was significantly lower among individuals with metabolic syndrome, with an odds ratio (OR) of 0.84 (95% CI: 0.76–0.93). Similarly, individuals with hypertension exhibited a reduced prevalence of AR (OR=0.85; 95% CI: 0.77–0.94), and those with impaired fasting glucose demonstrated a lower AR prevalence (OR=0.81; 95% CI: 0.73–0.89), further supporting a potential inverse correlation between AR and metabolic or cardiovascular conditions^9^.

A similar MR study has suggested that AR may have a protective effect against hypertension^15^. However, that study has notable limitations, including a small sample size and a lack of adjustment for potential confounders. In the present study, we employed a bidirectional, two-sample MR approach using summary statistics from large-scale GWAS to investigate the potential relationship between SBP and AR. This represents the most extensive MR study to date evaluating the impact of SBP-related genetic variants on AR risk. In our forward MR analysis, we rigorously selected 421 SNPs significantly associated with SBP (p<5×10^-8^) as IVs. These SNPs were chosen based on strict criteria to minimize pleiotropic effects, employing the *FastTraitR* package to exclude variants associated with known confounders, including calcium channel blocker use and cigarette smoking interaction. All IVs demonstrated adequate strength, with F-statistics exceeding the conventional threshold of 10. The primary MR analysis utilized three complementary methods: IVW, weighted median, and MR-Egger regression, all of which consistently suggested a potential inverse association between genetically predicted SBP and the risk of AR. Sensitivity analyses revealed no significant pleiotropy or outlier influence, and the use of two independent GWAS datasets enhanced the robustness of the findings. In contrast, the reverse MR analysis, using AR-associated SNPs as IVs (after removing confounding SNPs such as rs6771917), provided no evidence supporting an effect of AR on SBP, thus reinforcing the directionality of the observed association.

MR methodologies offer several advantages over conventional observational studies by mitigating issues such as reverse causality and unmeasured confounding. However, the biological mechanisms underpinning the association between reduced SBP and increased AR susceptibility remain poorly understood. According to the 2023 classification guidelines for allergic diseases issued by the European Academy of Allergy and Clinical Immunology (EAACI), allergic diseases are categorized into seven distinct types based on underlying immunological mechanisms^31^. Recent studies suggest that damage to the nasal mucosa caused by hypoperfusion may recruit eosinophils to promote repair of the injured tissue, with the resulting type II inflammation potentially exacerbating the progression of rhinitis^32^. Additionally, low perfusion of the nasal epithelium may drive polarization of M0 macrophages toward the M2 phenotype, further promoting the development of rhinitis^33^. Histamine, in particular, binds to H1 receptors to induce vasoconstriction and increased vascular permeability, both of which are relevant in the context of cardiovascular inflammation^34^. These inflammatory processes have been implicated in the development of myocardial remodeling and other CVDs^35-38^. Calcium-permeable ion channels, which constitute the pharmacological targets of many antihypertensive agents, have been implicated in the exacerbation of rhinitis through their blockade^39^. To minimize confounding by these mechanisms, we applied the *FastTraitR* package to exclude twenty SNPs associated with calcium channel blocker usage from our SBP instrument set, and we removed the SNP rs6771917 from the allergic rhinitis AR dataset due to its association with cardiac function.

### Strengths and limitations

Our MR framework leveraged summary statistics from large-scale GWAS and meta-analyses to achieve rigorous control of confounding and to mitigate reverse associations. These comprehensive datasets provided substantial statistical power and extensive genomic coverage. Nevertheless, several limitations merit consideration. First, European ancestry GWAS data, which may limit generalizability, and the absence of sex-specific SBP data in the database prevent assessment of differences between males and females. Second, unobserved pleiotropy cannot be addressed. Cochran’s Q test revealed notable heterogeneity among instrumental variables in both SBP and AR analyses under MR-Egger and inverse-variance weighted models, although this heterogeneity did not materially affect the estimates. Third, there may be potential violations of the MR assumptions. The two-sample MR design may be vulnerable to overidentification bias, potentially inflating SNP–exposure associations and gene-environment interactions not assessed. Fourth, there is a lack of relationships for diastolic blood pressure (DBP), mean blood pressure (MBP), or hypertension. The IEU Open GWAS database does not disaggregate SBP into distinct phenotypic subtypes, precluding subtype-specific inference regarding AR risk. Fifth, despite large sample sizes, heterogeneity led to wide confidence intervals and modest effect sizes. The effect of SBP on AR, though statistically robust, is limited in clinical utility and primarily offers theoretical insight into associations and multifactorial disease modeling rather than immediate practical application. Lastly, the lack of a significant association when we incorporated smoking as a moderating variable may be related to the definition of the smoking phenotype in the GWAS data and limitations in sample size.

In this study, MR analysis indicated that a 10 mmHg decrease in SBP is associated with an increased risk of AR (IVW, OR=0.997; 95% CI: 0.995–0.999). This effect may be modest, corresponding to only a 0.3% change in risk (1 minus 0.997), which is far below the conventional thresholds for clinically meaningful effects, such as an OR deviation of 0.1 or a risk change of ≥5%. This suggests that the impact is secondary and supportive rather than a primary determinant of AR onset or progression.

## CONCLUSIONS

This bidirectional MR study provides evidence supporting a potential relationship between genetically predicted lower SBP and increased risk of AR, while excluding reverse causation. Although exposure to a smoking environment may not play a dominant role in the SBP–AR association, it may nonetheless interact with other factors to jointly exert a mediating or moderating effect on this association. These findings highlight the intricate interplay between immune regulation and cardiovascular physiology and underscore the need for further mechanistic and clinical investigations.

## Supplementary Material



## Data Availability

The datasets used and/or analyzed during the current study are available from the corresponding author on reasonable request. The data supporting this research are available from the following source: DOI:10.6084/m9.figshare.29108483.

## References

[1] GBD 2019 Tobacco Collaborators. Spatial, temporal, and demographic patterns in prevalence of smoking tobacco use and attributable disease burden in 204 countries and territories, 1990-2019: a systematic analysis from the Global Burden of Disease Study 2019. Lancet. 2021;397(10292):2337-2360. doi: 10.1016/S0140-6736(21)01169-7 34051883 PMC8223261

[2] Shigehara K, Matsumoto N, Tsuge M, et al. Maternal smoking during infancy increases the risk of allergic diseases in children: a nationwide longitudinal survey in Japan. Allergy Asthma Clin Immunol. 2025;21(1):4. doi:10.1186/s13223-025-00952-9 39825417 PMC11740415

[3] Seo YG, Paek YJ, Kim JH, Kim JK, Noh HM. Relationship between heated tobacco product use and allergic rhinitis in Korean adults. Tob Induc Dis. 2023;21(November):146. doi:10.18332/tid/174130 37954489 PMC10632938

[4] Huang CF, Chie WC, Wang IJ. Effect of environmental exposures on allergen sensitization and the development of childhood allergic diseases: a large-scale population-based study. World Allergy Organ J. 2021;14(1):100495. doi: 10.1016/j.waojou.2020.100495 33510830 PMC7804989

[5] Fan H, Zhang X. Effects of smoking intensity trajectory, cumulative smoking exposure, and the number of years since quitting on the subsequent risk of hypertension. J Clin Hypertens (Greenwich). 2022;24(7):937-944. doi: 10.1111/jch.14534 35765239 PMC9278583

[6] Song S, Lee HA, Kim Y, Jeon BK, Moon CM, Park J. Dynamic changing smoking habits and cardiovascular events in patients newly diagnosed with hypertension, diabetes, or dyslipidemia: a national cohort study. Front Cardiovasc Med. 2023;10:1190227. doi:10.3389/fcvm.2023.1190227 37448792 PMC10336696

[7] Yao Z, Tasdighi E, Dardari ZA, et al. Association between cigarette smoking and subclinical markers of cardiovascular harm. J Am Coll Cardiol. 2025;85(10):1018-1034. doi:10.1016/j.jacc.2024.12.032 40074467 PMC12812482

[8] Cao X, Zhao G, Peng H, Mi Y, Zhou M, Guo Y. Association between allergic diseases and hypertension: co-occurrence pattern analysis. Allergy Asthma Proc. 2025;46(2):e61-e69. doi:10.2500/aap.2025.46.240110 40011983

[9] Hwang IC, Lee YJ, Ahn HY, Lee SM. Association between allergic rhinitis and metabolic conditions: a nationwide survey in Korea. Allergy Asthma Clin Immunol. 2016;12:5. doi:10.1186/s13223-015-0108-7 26807136 PMC4722723

[10] Wheatley LM, Togias A. Clinical practice. Allergic rhinitis. N Engl J Med. 2015;372(5):456-463. doi:10.1056/NEJMcp1412282 25629743 PMC4324099

[11] Kony S, Zureik M, Neukirch C, Leynaert B, Vervloet D, Neukirch F. Rhinitis is associated with increased systolic blood pressure in men: a population-based study. Am J Respir Crit Care Med. 2003;167(4):538-543. doi:10.1164/rccm.200208-851OC 12446269

[12] Sakallioglu O, Polat C, Akyigit A, Cetiner H, Duzer S. Allergic rhinitis and arterial blood pressure: a population-based study. J Laryngol Otol. 2018;132(5):418-422. doi:10.1017/S0022215118000580 29706138

[13] Ference BA, Ray KK, Catapano AL, et al. Mendelian Randomization study of ACLY and cardiovascular disease. N Engl J Med. 2019;380(11):1033-1042. doi:10.1056/NEJMoa1806747 30865797 PMC7612927

[14] Li MJ, Liu Z, Wang P, et al. GWASdb v2: an update database for human genetic variants identified by genome-wide association studies. Nucleic Acids Res. 2016;44(D1):D869-D876. doi:10.1093/nar/gkv1317 26615194 PMC4702921

[15] Zhang Y, Li X, Song Z, Yang Y. Association between allergic rhinitis and hypertension risk: a bidirectional 2-sample mendelian randomization study. Medicine (Baltimore). 2023;102(50):e36700. doi:10.1097/MD.0000000000036700 38115257 PMC10727617

[16] Evangelou E, Warren HR, Mosen-Ansorena D, et al. Genetic analysis of over 1 million people identifies 535 new loci associated with blood pressure traits. Nat Genet. 2018;50(10):1412-1425. doi:10.1038/s41588-018-0205-x 30224653 PMC6284793

[17] Dönertaş HM, Fabian DK, Valenzuela MF, Partridge L, Thornton JM. Common genetic associations between age-related diseases. Nat Aging. 2021;1(4):400-412. doi:10.1038/s43587-021-00051-5 33959723 PMC7610725

[18] Hemani G, Zheng J, Elsworth B, et al. The MR-Base platform supports systematic causal inference across the human phenome. Elife. 2018;7: e34408. doi:10.7554/eLife.34408 29846171 PMC5976434

[19] Skrivankova VW, Richmond RC, Woolf BAR, et al. Strengthening the reporting of observational studies in epidemiology using Mendelian randomization: the STROBE-MR statement. JAMA. 2021;326(16):1614-1621. doi:10.1001/jama.2021.18236 34698778

[20] Burgess S, Thompson SG; CRP CHD Genetics Collaboration. Avoiding bias from weak instruments in Mendelian randomization studies. Int J Epidemiol. 2011;40(3):755-764. doi:10.1093/ije/dyr036 21414999

[21] Bowden J, Del Greco M F, Minelli C, Davey Smith G, Sheehan N, Thompson J. A framework for the investigation of pleiotropy in two-sample summary data Mendelian randomization. Stat Med. 2017;36(11):1783-1802. doi:10.1002/sim.7221 28114746 PMC5434863

[22] Bowden J, Davey Smith G, Haycock PC, Burgess S. Consistent estimation in Mendelian randomization with some invalid instruments using a weighted median estimator. Genet Epidemiol. 2016;40(4):304-314. doi:10.1002/gepi.21965 27061298 PMC4849733

[23] Hartwig FP, Davey Smith G, Bowden J. Robust inference in summary data Mendelian randomization via the zero modal pleiotropy assumption. Int J Epidemiol. 2017;46(6):1985-1998. doi:10.1093/ije/dyx102 29040600 PMC5837715

[24] Mikshowsky AA, Gianola D, Weigel KA. Assessing genomic prediction accuracy for Holstein sires using bootstrap aggregation sampling and leave-one-out cross validation. J Dairy Sci. 2017;100(1):453-464. doi:10.3168/jds.2016-11496 27889124

[25] Tobacco. World Health Organization. June 25, 2025. Accessed April 6, 2026. https://www.who.int/news-room/fact-sheets/detail/tobacco

[26] Strzelak A, Ratajczak A, Adamiec A, Feleszko W. Tobacco smoke induces and alters immune responses in the lung triggering inflammation, allergy, asthma and other lung diseases: a mechanistic review. Int J Environ Res Public Health. 2018;15(5):1033. doi:10.3390/ijerph15051033 29883409 PMC5982072

[27] Nur Husna SM, Siti Sarah CO, Tan HT, Md Shukri N, Mohd Ashari NS, Wong KK. Reduced occludin and claudin-7 expression is associated with urban locations and exposure to second-hand smoke in allergic rhinitis patients. Sci Rep. 2021;11(1):1245. doi:10.1038/s41598-020-79208-y 33441633 PMC7806883

[28] Deslee G, Dury S, Perotin JM, et al. Bronchial epithelial spheroids: an alternative culture model to investigate epithelium inflammation-mediated COPD. Respir Res. 2007;8(1):86. doi:10.1186/1465-9921-8-86 18039378 PMC2214730

[29] Levy RV, Brathwaite KE, Sarathy H, Reidy K, Kaskel FJ, Melamed ML. Analysis of active and passive tobacco exposures and blood pressure in US children and adolescents. JAMA Netw Open. 2021;4(2):e2037936. doi:10.1001/jamanetworkopen.2020.37936 33620445 PMC7903259

[30] Lee TK, Jeon YJ, Jung SJ. Bi-directional association between allergic rhinitis and diabetes mellitus from the national representative data of South Korea. Sci Rep. 2021;11(1):4344. doi:10.1038/s41598-021-83787-9 33623055 PMC7902822

[31] Jutel M, Agache I, Zemelka-Wiacek M, et al. Nomenclature of allergic diseases and hypersensitivity reactions: Adapted to modern needs: An EAACI position paper. Allergy. 2023;78(11):2851-2874. doi:10.1111/all.15889 37814905

[32] Arnold IC, Munitz A. Spatial adaptation of eosinophils and their emerging roles in homeostasis, infection and disease. Nat Rev Immunol. 2024;24(12):858-877. doi:10.1038/s41577-024-01048-y 38982311

[33] Niu M, Wu H, Wang Y, et al. Macrophage polarization and allergic rhinitis: a review. Int Immunopharmacol. 2025;164:115334. doi:10.1016/j.intimp.2025.115334 40795498

[34] Gao S, Liu K, Ku W, et al. Histamine induced high mobility group box-1 release from vascular endothelial cells through H1 receptor. Front Immunol. 2022;13:930683. doi:10.3389/fimmu.2022.930683 36275732 PMC9583674

[35] Simpson JL, Phipps S, Baines KJ, Oreo KM, Gunawardhana L, Gibson PG. Elevated expression of the NLRP3 inflammasome in neutrophilic asthma. Eur Respir J. 2014;43(4):1067-1076. doi:10.1183/09031936.00105013 24136334

[36] Schmidt A, Éliás S, Joshi RN, Tegnér J. In vitro differentiation of human CD4+FOXP3+ induced regulatory T cells (iTregs) from naïve CD4+ T cells using a TGF-β-containing protocol. J Vis Exp. 2016;(118):55015. doi:10.3791/55015 28060341 PMC5226637

[37] Chen W. TGF-β regulation of T cells. Annu Rev Immunol. 2023;41:483-512. doi:10.1146/annurev-immunol-101921-045939 36750317 PMC12453633

[38] Greten FR, Grivennikov SI. Inflammation and cancer: triggers, mechanisms, and consequences. Immunity. 2019;51(1):27-41. doi:10.1016/j.immuni.2019.06.025 31315034 PMC6831096

[39] Lin H, Zheng C, Li J, Yang C, Hu L. Ca2+ -activated K+ channel-3.1 blocker TRAM-34 alleviates murine allergic rhinitis. Int Immunopharmacol. 2014;23(2):642-648. doi:10.1016/j.intimp.2014.10.017 25466273

