# Supplementary material for: Low systolic blood pressure increases risk of allergic rhinitis: Evidence from a bidirectional Mendelian randomization study accounting for smoking environment
Source: Tob Induc Dis. 2026 Jun 19;24:10.18332/tid/220356. doi: 10.18332/tid/220356 (PMC13281030; doi:10.18332/tid/220356)
Supplement: Supplementary file 1 [file TID-24-94-s1.pdf]

Suppl-Table1 STROBE-MR checklist of recommended items to address in reports of Mendelian randomization studies[1 2]

Item No.	Section		Checklist item	Page No.	Relevant text from manuscript
1	**TITLE and ABSTRACT**		Indicate Mendelian randomization (MR) as the study's design in the title and/or the abstract if that is a main purpose of the study	1	TITLE and ABSTRACT
	**INTRODUCTION**				
2	**Background**		Explain the scientific background and rationale for the reported study. What is the exposure? Is a potential causal relationship between exposure and outcome plausible? Justify why MR is a helpful method to address the study question	3-4	Line 80-97
3	**Objectives**		State specific objectives clearly, including pre-specified causal hypotheses (if any). State that MR is a method that, under specific assumptions, intends to estimate causal effects	4	Line 104-109
	**METHODS**				
4	**Study design and data sources**		Present key elements of the study design early in the article. Consider including a table listing sources of data for all phases of the study. For each data source contributing to the analysis, describe the following:		
		a)	Setting: Describe the study design and the underlying population, if possible. Describe the setting, locations, and relevant dates, including periods of recruitment, exposure, follow-up, and data collection, when available.	4-5	Line113-121
		b)	Participants: Give the eligibility criteria, and the sources and methods of selection of participants. Report the sample size, and whether any power or sample size calculations were carried out prior to the main analysis	4-5	Line113-121
		c)	Describe measurement, quality control and selection of genetic variants	4-5	Line113-121
		d)	For each exposure, outcome, and other relevant variables, describe methods of assessment and diagnostic criteria for diseases	5	Table1
		e)	Provide details of ethics committee approval and participant informed consent, if relevant		N/A
5	**Assumptions**		Explicitly state the three core IV assumptions for the main analysis (relevance, independence and exclusion restriction) as well assumptions for any additional or sensitivity analysis	6	Line 160-167
6	**Statistical methods: main analysis**		Describe statistical methods and statistics used		

		a)	Describe how quantitative variables were handled in the analyses (i.e., scale, units, model)	6	Line 148
		b)	Describe how genetic variants were handled in the analyses and, if applicable, how their weights were selected	6	Line 142-147
		c)	Describe the MR estimator (e.g. two-stage least squares, Wald ratio) and related statistics. Detail the included covariates and, in case of two-sample MR, whether the same covariate set was used for adjustment in the two samples	6	Line 164-165
		d)	Explain how missing data were addressed	5-6	Line 136-146
		e)	If applicable, indicate how multiple testing was addressed	6-7	Line 160-179
7	**Assessment of assumptions**		Describe any methods or prior knowledge used to assess the assumptions or justify their validity	7	Line 175-179
8	**Sensitivity analyses and additional analyses**		Describe any sensitivity analyses or additional analyses performed (e.g. comparison of effect estimates from different approaches, independent replication, bias analytic techniques, validation of instruments, simulations)	7	Line 174
9	**Software and pre-registration**				
		a)	Name statistical software and package(s), including version and settings used	6	Line 162
		b)	State whether the study protocol and details were pre-registered (as well as when and where)		
	**RESULTS**				
10	**Descriptive data**				
		a)	Report the numbers of individuals at each stage of included studies and reasons for exclusion. Consider use of a flow diagram	7	Line 189, Table 2
		b)	Report summary statistics for phenotypic exposure(s), outcome(s), and other relevant variables (e.g. means, SDs, proportions)	8	Table 2
		c)	If the data sources include meta-analyses of previous studies, provide the assessments of heterogeneity across these studies		N/A
		d)	For two-sample MR:<br>i. Provide justification of the similarity of the genetic variant-exposure associations between the exposure and outcome samples<br>ii. Provide information on the number of individuals who overlap between the	7	Table 2

			exposure and outcome studies		
11	**Main results**				
		a)	Report the associations between genetic variant and exposure, and between genetic variant and outcome, preferably on an interpretable scale	7	Table 2
		b)	Report MR estimates of the relationship between exposure and outcome, and the measures of uncertainty from the MR analysis, on an interpretable scale, such as odds ratio or relative risk per SD difference	7	Table 2
		c)	If relevant, consider translating estimates of relative risk into absolute risk for a meaningful time period		N/A
		d)	Consider plots to visualize results (e.g. forest plot, scatterplot of associations between genetic variants and outcome versus between genetic variants and exposure)	8	Figure 3,4
12	**Assessment of assumptions**				
		a)	Report the assessment of the validity of the assumptions	8-9	Line 218-227
		b)	Report any additional statistics (e.g., assessments of heterogeneity across genetic variants, such as I^2, Q statistic or E-value)	9	Table 3
13	**Sensitivity analyses and additional analyses**				
		a)	Report any sensitivity analyses to assess the robustness of the main results to violations of the assumptions	8	Line 209-215
		b)	Report results from other sensitivity analyses or additional analyses	8	Line 209-215
		c)	Report any assessment of direction of causal relationship (e.g., bidirectional MR)	8	Line 218-227
		d)	When relevant, report and compare with estimates from non-MR analyses		N/A
		e)	Consider additional plots to visualize results (e.g., leave-one-out analyses)	8	Suppl-Figure 1
	**DISCUSSION**				
14	**Key results**		Summarize key results with reference to study objectives	9-10	Line 245-261
15	**Limitations**		Discuss limitations of the study, taking into account the validity of the IV assumptions, other sources of potential bias, and imprecision. Discuss both direction and magnitude of any potential bias and any efforts to address them	11	Line 282-293

16	**Interpretation**				
		a)	Meaning: Give a cautious overall interpretation of results in the context of their limitations and in comparison with other studies	9	Line 234-244
		b)	Mechanism: Discuss underlying biological mechanisms that could drive a potential causal relationship between the investigated exposure and the outcome, and whether the gene-environment equivalence assumption is reasonable. Use causal language carefully, clarifying that IV estimates may provide causal effects only under certain assumptions	10	Line 262-271
		c)	Clinical relevance: Discuss whether the results have clinical or public policy relevance, and to what extent they inform effect sizes of possible interventions	11	Line 296-298
17	**Generalizability**		Discuss the generalizability of the study results (a) to other populations, (b) across other exposure periods/timings, and (c) across other levels of exposure	11	Line 296-298
	**OTHER INFORMATION**				
18	**Funding**		Describe sources of funding and the role of funders in the present study and, if applicable, sources of funding for the databases and original study or studies on which the present study is based	12	Line 318
19	**Data and data sharing**		Provide the data used to perform all analyses or report where and how the data can be accessed, and reference these sources in the article. Provide the statistical code needed to reproduce the results in the article, or report whether the code is publicly accessible and if so, where	12	Line 315
20	**Conflicts of Interest**		All authors should declare all potential conflicts of interest	12	Line 305

This checklist is copyrighted by the Equator Network under the Creative Commons Attribution 3.0 Unported (CC BY 3.0) license.

1. Skrivankova VW, Richmond RC, Woolf BAR, Yarmolinsky J, Davies NM, Swanson SA, et al. Strengthening the Reporting of Observational Studies in Epidemiology using Mendelian Randomization (STROBE-MR) Statement. JAMA. 2021;under review.

2. Skrivankova VW, Richmond RC, Woolf BAR, Davies NM, Swanson SA, VanderWeele TJ, et al. Strengthening the Reporting of Observational Studies in Epidemiology using Mendelian Randomisation (STROBE-MR): Explanation and Elaboration. BMJ. 2021;375:n2233.

Suppl-Table2 Association analysis for Systolic blood pressure (SBP)-decreasing GWAS risk alleles with the diagnosis of Allergic rhinitis (AR), IEU OpenGWAS 2018 and 2021 (N=1,242,119)**.**

Chr	Position	SNP	EA	EAF	SBP			AR		
					β	SE	P	β	SE	P
1	113044328	rs10776752	G	0.926031	0.8211	0.0576	4.16E-46	0.000787182	0.000900106	0.36
1	207208335	rs10864035	C	0.599411	-0.1793	0.0307	5.21E-09	-0.000407657	0.000479932	0.4
1	180865798	rs10914124	T	0.614208	-0.234	0.0312	6.38E-14	-0.000375914	0.000483505	0.45
1	217736417	rs11117882	A	0.769789	0.2741	0.0357	1.62E-14	0.000454308	0.000559257	0.41
1	41865293	rs11210029	A	0.638119	0.203	0.0313	8.84E-11	-0.000228466	0.000490299	0.63
1	150572037	rs11585169	T	0.433082	0.1796	0.0308	5.50E-09	0.000188316	0.000478497	0.68
1	197297417	rs12042924	T	0.538043	0.1807	0.0303	2.47E-09	-0.000990622	0.000474248	0.035
1	59621911	rs12063372	G	0.61908	0.1989	0.0318	3.98E-10	-0.000472027	0.000488524	0.34
1	43765089	rs1209384	A	0.380785	-0.2558	0.0313	3.02E-16	-0.000592859	0.000485411	0.24
1	67007389	rs12136922	G	0.493887	0.2027	0.0304	2.60E-11	0.000861138	0.000473011	0.065
1	28707612	rs12137056	C	0.689976	-0.2327	0.0328	1.30E-12	-0.00125482	0.000509859	0.016
1	169090660	rs12731646	C	0.601369	-0.189	0.0307	7.44E-10	0.000688354	0.000483289	0.16
1	42364877	rs1408945	G	0.581156	-0.3196	0.0304	7.51E-26	-0.0000262061	0.000478597	0.97
1	243387788	rs1565440	G	0.619181	0.1746	0.0311	1.98E-08	0.00112635	0.000485946	0.017
1	228204279	rs1745417	C	0.488672	0.2871	0.0301	1.45E-21	0.000555705	0.00047059	0.24
1	16348729	rs1889785	G	0.538269	0.1782	0.0304	4.58E-09	-0.000369988	0.000472045	0.4
1	6659505	rs2232460	G	0.66458	-0.2171	0.032	1.17E-11	-0.0010477	0.000497612	0.036
1	230849359	rs2493134	T	0.581316	0.3736	0.0309	1.18E-33	-0.000155181	0.000481571	0.75
1	3327032	rs2493296	C	0.862986	0.4183	0.0442	2.97E-21	0.000521921	0.000684983	0.44
1	2164116	rs263532	T	0.573043	-0.1798	0.0307	4.72E-09	0.000467342	0.000477031	0.32
1	207974818	rs2724377	A	0.533345	-0.1938	0.0301	1.21E-10	0.000765808	0.000471996	0.095
1	27407850	rs34079867	C	0.743035	0.1992	0.0354	1.83E-08	-0.00011807	0.000542262	0.79

1	25366987	rs404100	C	0.556986	0.1935	0.0303	1.70E-10	-0.000211038	0.000473475	0.65
1	184585182	rs4651224	C	0.547085	0.1986	0.0306	8.57E-11	-0.000063945	0.000478535	0.89
1	10767902	rs488834	C	0.241479	-0.3799	0.0365	2.27E-25	-0.000405596	0.000550778	0.44
1	249155909	rs4926499	G	0.169938	0.2965	0.0438	1.29E-11	0.001108	0.000641394	0.073
1	115827266	rs59980837	G	0.981267	1.0997	0.1163	3.21E-21	-0.00253252	0.00173719	0.13
1	56979681	rs61772592	A	0.879433	0.3181	0.0455	2.73E-12	0.000464594	0.000724763	0.51
1	78555928	rs658780	T	0.746763	0.2028	0.0347	5.08E-09	-0.000202432	0.000543129	0.67
1	11881441	rs6699618	C	0.838416	-0.9115	0.041	1.7E-09	0.00130475	0.000638064	0.035
1	221265336	rs72742507	C	0.701294	-0.2053	0.0328	3.87E-10	0.000369105	0.000514999	0.46
1	94051350	rs7514579	A	0.765539	-0.2243	0.0361	5.19E-10	-0.000142014	0.000560894	0.77
1	15810172	rs75461554	C	0.800626	-0.3016	0.0377	1.24E-15	0.000848881	0.000588304	0.17
1	209970355	rs7555285	G	0.194025	0.2294	0.0376	1.05E-09	0.00110516	0.000593005	0.052
1	156129796	rs76719272	C	0.873915	-0.2738	0.0461	2.86E-09	-0.00160727	0.000718019	0.025
1	56606206	rs778124	G	0.62461	0.2965	0.0311	1.52E-21	-0.0000449278	0.000485552	0.91
1	1684169	rs7796	C	0.514719	-0.3385	0.0314	4.27E-27	0.000847499	0.00047339	0.074
1	89242954	rs786923	C	0.372888	-0.3082	0.031	2.73E-23	0.000190707	0.000488406	0.75
2	174977976	rs10048760	T	0.522059	0.1862	0.0301	6.17E-10	-0.000352782	0.000473833	0.44
2	66773469	rs10188003	C	0.614465	0.1883	0.0307	8.59E-10	0.0000495999	0.000483423	0.93
2	112744260	rs10207726	C	0.70754	-0.2142	0.033	8.53E-11	0.000650568	0.000515904	0.21
2	230629138	rs1044822	C	0.849145	-0.248	0.0424	4.94E-09	-0.000292704	0.000658567	0.63
2	227185749	rs10804330	T	0.574475	-0.2351	0.0306	1.55E-14	-0.000388135	0.000479994	0.41
2	43196694	rs115262049	A	0.914885	-0.5893	0.0552	1.32E-26	-0.000331915	0.000848446	0.67
2	43397614	rs12464602	G	0.376439	-0.2437	0.0315	1.02E-14	-0.000186384	0.000491291	0.76
2	182987704	rs12473915	G	0.790134	-0.295	0.0375	3.64E-15	0.0016302	0.00057796	0.0044
2	204085635	rs12693982	C	0.604436	0.2575	0.0309	7.86E-17	0.000632503	0.00048062	0.18
2	213188795	rs12694277	T	0.305937	0.2018	0.0335	1.70E-09	0.000364468	0.000522974	0.45

2	26911745	rs1275985	C	0.393111	-0.5411	0.0308	4.32E-69	-0.0000113924	0.000485125	1
2	55779476	rs13016772	C	0.24727	0.2522	0.0355	1.21E-12	-0.000555535	0.000547635	0.3
2	191634958	rs13412750	G	0.727771	-0.2889	0.0341	2.41E-17	0.0004818	0.00052858	0.37
2	37517566	rs13420463	A	0.757636	-0.3143	0.036	2.53E-18	0.00128344	0.000553158	0.022
2	242344695	rs139354822	T	0.973491	-0.6115	0.0975	3.57E-10	0.00126263	0.0015059	0.39
2	135630498	rs17257081	A	0.820846	-0.2274	0.0392	6.59E-09	0.000645846	0.000616843	0.3
2	177540601	rs17610485	T	0.432802	0.1738	0.0304	1.08E-08	-0.000169365	0.000478247	0.73
2	19744462	rs17760259	T	0.581208	0.2654	0.0304	2.54E-18	0.0000686197	0.000477009	0.88
2	218680529	rs2161967	T	0.435166	-0.2836	0.0307	2.52E-20	0.00045274	0.00047859	0.38
2	65287896	rs2249105	A	0.631967	-0.2927	0.0313	8.65E-21	0.000120239	0.000485963	0.78
2	25187115	rs2384063	C	0.239096	0.3266	0.0357	5.78E-20	-0.0000531704	0.000550163	0.93
2	121996007	rs2580350	G	0.4444	0.1769	0.0307	8.30E-09	0.000334227	0.000476602	0.45
2	164954174	rs268263	T	0.257667	0.5938	0.0353	1.70E-63	0.00081611	0.000546348	0.14
2	231279616	rs3754944	C	0.42044	0.1768	0.0308	9.45E-09	0.000491751	0.000478169	0.3
2	218779144	rs3828282	C	0.441672	-0.1857	0.0318	5.23E-09	0.000209162	0.000477724	0.68
2	208521512	rs3845811	C	0.57498	0.2942	0.0309	1.71E-21	-0.000370068	0.00047691	0.42
2	73403040	rs4577304	T	0.514474	0.1767	0.0302	4.89E-09	-0.000612263	0.000473397	0.19
2	180738654	rs4894132	T	0.733488	-0.2469	0.0342	5.23E-13	0.000339893	0.000531949	0.57
2	40555733	rs4952609	A	0.745277	-0.2124	0.0347	9.30E-10	0.000674738	0.000544061	0.21
2	162278233	rs55732192	G	0.908115	-0.3358	0.0521	1.15E-10	0.0000745445	0.000817316	0.91
2	145726621	rs55944332	A	0.76984	0.2613	0.0355	1.83E-13	-0.00173076	0.000561207	0.0022
2	146989797	rs62170470	T	0.603697	-0.1972	0.0321	8.08E-10	0.000240457	0.000489774	0.64
2	68503044	rs6731373	G	0.646512	0.1913	0.0326	4.41E-09	0.000116101	0.000491469	0.78
2	69534650	rs6732123	G	0.577305	-0.1737	0.0307	1.53E-08	-0.000014017	0.00047832	0.96
2	114083120	rs6737318	A	0.780908	-0.2348	0.0364	1.11E-10	0.000268831	0.000569955	0.7
2	177053298	rs71421551	G	0.7176	0.2374	0.0333	1.01E-12	-0.000641095	0.000522242	0.2

2	86326717	rs72847885	A	0.663878	-0.2413	0.0318	3.25E-14	0.000452308	0.000498303	0.36
3	41925398	rs1052501	C	0.17186	0.2262	0.0412	4.01E-08	0.000565597	0.00063456	0.37
3	14943965	rs11925504	G	0.426906	-0.2901	0.0305	1.88E-21	-0.000885285	0.000475291	0.058
3	138101529	rs1199330	A	0.878846	0.2654	0.047	1.63E-08	0.000738555	0.000724942	0.34
3	121682388	rs12637573	A	0.470105	0.1731	0.0302	9.94E-09	0.000885701	0.000471903	0.059
3	169096900	rs1290784	C	0.55331	0.4124	0.0303	3.47E-42	-0.000104778	0.000473255	0.84
3	185322643	rs13081203	G	0.649976	0.2221	0.0323	6.15E-12	-0.000563171	0.00049313	0.26
3	85656311	rs1375564	C	0.371727	0.2579	0.0315	2.67E-16	-0.000555785	0.000493627	0.25
3	20073193	rs189267552	T	0.987947	-0.8664	0.139	4.57E-10	-0.000353185	0.00217075	0.85
3	169325621	rs2111557	C	0.53621	0.1764	0.0302	5.19E-09	0.0005436	0.000472268	0.24
3	183435713	rs262986	G	0.525042	-0.2371	0.0305	7.62E-15	0.0000426337	0.000473411	0.94
3	153732022	rs35288962	G	0.738635	0.2299	0.0344	2.34E-11	0.000489156	0.000534836	0.37
3	56771251	rs3772219	A	0.67124	-0.2733	0.0324	3.31E-17	0.000379084	0.000500956	0.44
3	168697602	rs3980686	G	0.886256	-0.4998	0.0487	1.04E-24	0.000402373	0.000746838	0.63
3	70920485	rs4499560	A	0.32263	0.2199	0.0326	1.53E-11	-0.000120694	0.000509274	0.8
3	169534538	rs4955575	A	0.748361	-0.2158	0.0348	5.61E-10	-0.000609845	0.000542173	0.28
3	124557643	rs6438857	T	0.57805	-0.2736	0.0305	2.95E-19	-0.000297083	0.000477156	0.51
3	53562894	rs6445583	G	0.254131	0.2774	0.0349	1.89E-15	-0.000196135	0.000539924	0.68
3	158212823	rs6788907	G	0.735814	0.221	0.0339	7.07E-11	-0.000609461	0.000532007	0.25
3	41107173	rs6788984	A	0.852482	-0.2999	0.0432	3.86E-12	-0.000507277	0.000669125	0.46
3	27704702	rs68115553	A	0.982055	0.6445	0.1143	1.71E-08	0.000285983	0.00177882	0.87
3	37598382	rs743395	C	0.626369	0.2597	0.0317	2.56E-16	-0.0000734299	0.000494768	0.86
3	53143901	rs7615099	A	0.67146	-0.1891	0.0321	3.84E-09	0.000626991	0.000502425	0.22
3	66422246	rs7618284	G	0.651076	-0.1891	0.0331	1.11E-08	0.000189522	0.00050589	0.73
3	154680449	rs79539362	T	0.89873	-0.4003	0.0504	1.98E-15	-0.000320121	0.000777806	0.66
3	11495983	rs9848170	G	0.405959	0.3231	0.0307	6.67E-26	0.000267085	0.000479489	0.58

3	196228360	rs9869437	C	0.643669	-0.2001	0.0318	3.12E-10	0.000509254	0.000494721	0.29
3	141152017	rs9876694	C	0.935221	0.4713	0.0651	4.50E-13	-0.00124405	0.000973412	0.2
3	133949366	rs9880098	G	0.622195	0.3081	0.0308	1.47E-23	-0.000142983	0.000486369	0.78
4	106911321	rs11097909	T	0.146694	0.3628	0.043	3.25E-17	0.0009359	0.000664125	0.16
4	46595623	rs12511987	T	0.819804	0.2329	0.0399	5.31E-09	-0.000341634	0.000616039	0.58
4	156639846	rs12643599	A	0.637865	-0.3134	0.0313	1.34E-23	-0.000517543	0.000487749	0.26
4	2668217	rs1290933	C	0.320046	-0.2847	0.0327	3.14E-18	-0.00108206	0.000507712	0.033
4	63768826	rs13107261	G	0.627858	-0.1778	0.0314	1.49E-08	0.000288279	0.000487055	0.61
4	103188709	rs13107325	C	0.92814	-0.9086	0.0592	3.65E-53	-0.00477901	0.000911337	0.0000 0016
4	89750668	rs13149209	T	0.779156	-0.281	0.0367	1.91E-14	0.000597224	0.000569472	0.26
4	108861082	rs1493132	T	0.655236	0.1766	0.0318	2.80E-08	0.000151986	0.000494751	0.77
4	86719165	rs17010957	T	0.851651	0.534	0.043	2.07E-35	0.00129727	0.000666861	0.05
4	157678511	rs17035181	T	0.852965	-0.3074	0.0429	7.75E-13	-0.000876363	0.00066418	0.2
4	111408718	rs1814951	G	0.118241	-0.3231	0.0466	4.11E-12	-0.000170974	0.000731527	0.83
4	38387244	rs2291434	G	0.467921	-0.2622	0.0303	5.00E-18	-0.000747967	0.000471644	0.11
4	145740898	rs2353940	T	0.755468	0.2075	0.0358	6.79E-09	0.00098352	0.000551553	0.071
4	3451109	rs2498323	G	0.901659	0.3171	0.0517	8.60E-10	-0.000198134	0.000788366	0.78
4	18008232	rs2610990	A	0.259232	0.2903	0.0343	2.59E-17	-0.000274576	0.000536002	0.59
4	157004869	rs28634038	T	0.666829	0.18	0.032	1.86E-08	0.000876921	0.000504178	0.079
4	26785356	rs28667801	A	0.594081	0.2627	0.0314	5.95E-17	0.00071875	0.000481938	0.14
4	2246927	rs34535756	C	0.959133	0.478	0.0786	1.19E-09	-0.00064653	0.00119495	0.55
4	144085327	rs4690774	A	0.521206	-0.21	0.0303	4.19E-12	-0.000629456	0.00047218	0.17
4	156398688	rs4691666	C	0.471824	-0.3631	0.0302	2.68E-33	-0.000101353	0.000472106	0.89
4	120555696	rs4834792	T	0.519145	0.1973	0.0303	7.44E-11	0.000276509	0.000471411	0.51
4	77414988	rs5020545	C	0.564266	-0.2179	0.0305	9.05E-13	-0.000748059	0.000477207	0.12

4	83830244	rs60909079	G	0.735294	-0.2114	0.0351	1.71E-09	-0.000221811	0.000534	0.72
4	54801228	rs60991988	T	0.881773	-0.3789	0.0498	2.77E-14	-0.000356622	0.000746581	0.63
4	48713862	rs62309747	G	0.539591	-0.2244	0.0304	1.56E-13	0.000304726	0.000471868	0.49
4	138464842	rs7439567	C	0.58602	0.2537	0.0309	2.21E-16	0.000432354	0.000479681	0.38
4	169688000	rs869396	C	0.529528	-0.2115	0.0305	4.08E-12	0.00012765	0.000474776	0.8
5	127411454	rs10045307	C	0.764705	0.2024	0.0362	2.26E-08	0.000357483	0.000560127	0.54
5	1279790	rs10069690	C	0.735504	0.3098	0.0369	4.63E-17	0.00041482	0.000534821	0.43
5	33194751	rs10941043	T	0.712595	0.2585	0.0332	6.91E-15	-0.000066256	0.000519243	0.88
5	114428167	rs11241313	C	0.689106	-0.2071	0.0326	2.11E-10	-0.000257245	0.000508334	0.62
5	157817634	rs11960210	T	0.624788	-0.4727	0.0313	1.57E-51	0.000164903	0.000485352	0.79
5	179411477	rs12153395	G	0.889049	-0.3303	0.0486	1.07E-11	-0.00132779	0.000756087	0.084
5	32831939	rs12656497	T	0.403652	0.6382	0.0307	5.53E-96	-0.000482899	0.000478421	0.33
5	61940569	rs12657950	C	0.93318	0.455	0.059	1.24E-14	-0.000901894	0.000939237	0.32
5	157938070	rs13358657	A	0.869271	0.388	0.0445	2.80E-18	-0.000307676	0.000696386	0.63
5	122470209	rs1422279	C	0.613392	0.331	0.0309	8.94E-27	0.000700034	0.000484571	0.14
5	53276301	rs1664781	G	0.302619	0.2643	0.0326	5.17E-16	0.000579568	0.000511402	0.23
5	97953719	rs1871190	G	0.679512	0.1954	0.0324	1.63E-09	0.00054384	0.000508388	0.28
5	157474590	rs1957563	C	0.733561	0.3629	0.0342	2.65E-26	-0.000435804	0.000531259	0.44
5	68007803	rs246973	C	0.712901	0.2479	0.0335	1.36E-13	-0.000411227	0.000528002	0.45
5	55861464	rs28650790	C	0.81005	0.2287	0.0387	3.43E-09	0.000837036	0.000597925	0.16
5	141726983	rs2913920	C	0.228986	0.2418	0.0359	1.64E-11	0.000556625	0.000561224	0.32
5	173301427	rs3860770	G	0.722152	-0.2663	0.0333	1.27E-15	0.000266982	0.000526259	0.61
5	361148	rs4957026	A	0.337669	-0.1982	0.0323	8.45E-10	-0.000150487	0.000497424	0.79
5	87389027	rs6452769	G	0.789503	-0.3143	0.0377	7.63E-17	0.00196385	0.000583439	0.00087
5	63831964	rs6870654	T	0.738898	-0.2136	0.0347	7.48E-10	-0.0000485462	0.00053545	0.87

5	127845030	rs6892983	C	0.582479	0.3427	0.0307	6.20E-29	0.0000532538	0.000481562	0.91
5	140086677	rs702395	C	0.564365	0.2318	0.0305	2.96E-14	-0.000241181	0.00047374	0.64
5	66280577	rs73103937	T	0.732255	-0.2051	0.0344	2.49E-09	-0.0004047	0.000536566	0.49
5	96211594	rs76443575	G	0.964022	-0.5233	0.0816	1.43E-10	0.00152063	0.00126258	0.2
5	67678506	rs7703560	A	0.690194	0.2246	0.0333	1.53E-11	-0.000247686	0.000515254	0.63
5	50818437	rs7722243	G	0.491719	-0.2048	0.0302	1.19E-11	0.000467022	0.000472633	0.32
5	15695987	rs7725413	C	0.232201	-0.1985	0.0359	3.22E-08	-0.000807512	0.000558273	0.15
5	122835051	rs9327297	C	0.671138	-0.2747	0.0319	7.22E-18	-0.00114244	0.000500222	0.021
6	161720674	rs10214535	A	0.684153	-0.1806	0.0327	3.33E-08	-0.000552224	0.000512679	0.27
6	126228512	rs10782230	G	0.521833	0.2106	0.0302	3.09E-12	0.00068253	0.000469769	0.14
6	43760327	rs11967262	C	0.513025	0.1715	0.0311	3.50E-08	-0.000848509	0.000472675	0.068
6	30988017	rs12528975	G	0.950173	0.4751	0.0747	2.02E-10	-0.00115502	0.00107721	0.28
6	163737476	rs12661036	T	0.77201	0.2104	0.0374	1.85E-08	0.000365268	0.000571915	0.51
6	7715689	rs1575290	C	0.527568	0.1973	0.0301	5.57E-11	0.000515397	0.000471539	0.27
6	12295987	rs1630736	C	0.543982	-0.1706	0.0309	3.37E-08	-0.000791443	0.000476774	0.11
6	151004770	rs17080102	G	0.926348	-0.8085	0.0594	3.44E-42	-0.00111727	0.000900333	0.22
6	79657391	rs1984195	G	0.510145	0.2409	0.0303	1.86E-15	0.000748509	0.000470823	0.11
6	22056923	rs2294214	A	0.690639	-0.2388	0.0333	7.44E-13	-0.00071944	0.000508428	0.14
6	134209837	rs2327429	T	0.703727	-0.2	0.0338	3.28E-09	-0.000286986	0.000517672	0.59
6	31762843	rs2607015	G	0.58506	0.4465	0.0309	2.51E-47	0.000393081	0.000477642	0.4
6	22392260	rs2744133	A	0.727242	-0.2879	0.0337	1.31E-17	-0.000647553	0.000528012	0.21
6	1613686	rs2745599	A	0.540183	-0.2164	0.0317	8.70E-12	0.000938957	0.000477105	0.054
6	39262535	rs2815063	C	0.864106	0.2755	0.0458	1.80E-09	-0.0000871703	0.000698426	0.87
6	159680744	rs294886	T	0.281629	-0.2318	0.034	9.25E-12	0.000720878	0.000524629	0.15
6	97066242	rs6921291	C	0.813242	0.3575	0.0385	1.61E-20	0.000826576	0.000603308	0.19
6	166180947	rs6932812	C	0.923889	-0.4603	0.0615	7.18E-14	-0.00165202	0.000900639	0.074

6	152160286	rs75559245	A	0.915932	-0.3122	0.0529	3.60E-09	0.00124415	0.000846765	0.14
6	43349215	rs7763558	G	0.668886	0.3363	0.0321	1.11E-25	-0.000827238	0.000501914	0.095
6	147713764	rs7765526	A	0.462221	-0.201	0.0307	5.86E-11	0.000139926	0.000476591	0.81
6	50683009	rs78648104	T	0.915307	0.4287	0.0541	2.30E-15	-0.00289546	0.000843209	0.00067
6	25882678	rs79782817	G	0.902797	0.5324	0.0499	1.42E-26	0.00148546	0.000793075	0.057
6	143200936	rs8180684	C	0.710665	0.2134	0.0335	1.89E-10	-0.000152361	0.000520248	0.74
6	20685486	rs9356744	T	0.684812	0.2026	0.0325	4.55E-10	0.000393724	0.000507664	0.41
6	82235408	rs9361836	C	0.690632	0.2196	0.0324	1.22E-11	-0.000275316	0.000508998	0.54
6	127159982	rs9401913	G	0.568279	0.5202	0.0305	3.17E-65	-0.00115503	0.000474701	0.013
6	109013930	rs9486916	C	0.79197	0.2657	0.0385	5.15E-12	-0.000133987	0.000603762	0.86
6	117522156	rs961764	C	0.431956	0.1909	0.0305	3.87E-10	-0.000837913	0.000475487	0.089
7	151413194	rs10224210	T	0.725327	0.3831	0.034	1.90E-29	-0.000316381	0.000529033	0.56
7	2529623	rs10282122	C	0.330395	-0.302	0.0327	2.57E-20	0.000877065	0.000500197	0.074
7	24735004	rs112509803	G	0.884645	-0.2641	0.0477	3.08E-08	-0.000686804	0.000741819	0.38
7	46008110	rs11977526	G	0.597477	-0.3213	0.0312	7.19E-25	-0.000404494	0.000479787	0.38
7	47548893	rs12668436	T	0.752164	0.2151	0.035	7.96E-10	-0.000128991	0.000544312	0.81
7	155744303	rs1870735	C	0.451641	-0.206	0.0311	3.50E-11	-0.00109301	0.000476816	0.021
7	19039605	rs28688791	T	0.802735	0.3222	0.038	2.27E-17	0.000109028	0.000594721	0.84
7	130432469	rs34072724	G	0.514552	-0.2422	0.0303	1.31E-15	-0.000462175	0.000471588	0.32
7	130973495	rs35680304	C	0.41393	0.2694	0.031	3.61E-18	-0.000369523	0.000483287	0.43
7	18543250	rs3807925	A	0.653141	0.1859	0.0319	5.62E-09	0.00119644	0.000495689	0.012
7	92237426	rs42032	G	0.739787	-0.3231	0.0345	7.59E-21	0.000838951	0.000537832	0.11
7	106412082	rs62481856	G	0.802751	0.7448	0.0376	2.51E-87	0.000174549	0.000590308	0.73
7	90297177	rs67617547	C	0.673205	-0.1799	0.0322	2.31E-08	-0.000542938	0.000502645	0.26
7	27328187	rs6961048	C	0.899337	0.5304	0.0497	1.38E-26	-0.0000307613	0.00078105	0.99

7	1966841	rs6978112	C	0.582366	0.2286	0.0309	1.38E-13	-0.000160511	0.000480056	0.74
7	4669949	rs73049928	A	0.80798	0.2382	0.0392	1.23E-09	0.000237218	0.000600553	0.72
7	149474622	rs73727605	G	0.933392	0.3616	0.0623	6.47E-09	0.0000862811	0.000942125	0.94
7	131321010	rs75672964	C	0.957696	0.5885	0.0839	2.31E-12	0.000367254	0.0011784	0.75
7	77572461	rs848445	T	0.282401	0.2025	0.0339	2.32E-09	0.000320349	0.000522288	0.52
7	28650761	rs977184	T	0.638869	0.184	0.0314	4.63E-09	-0.0000362596	0.000494638	0.98
8	23401534	rs10866828	C	0.733101	0.2476	0.0355	3.07E-12	0.000572068	0.000537749	0.3
8	68920135	rs13253358	C	0.688999	0.2127	0.033	1.15E-10	0.000779453	0.000512944	0.12
8	81386066	rs148401029	C	0.965174	-0.4623	0.0848	4.99E-08	0.00149902	0.00128214	0.25
8	101674751	rs1786345	A	0.556991	-0.2073	0.0307	1.45E-11	-0.00107233	0.000475615	0.031
8	10640065	rs1821002	C	0.401949	-0.3794	0.0307	4.39E-35	-0.000872561	0.000480371	0.084
8	38130025	rs1906672	G	0.767988	0.2966	0.0358	1.18E-16	-0.000455086	0.000556912	0.37
8	76878957	rs2126474	G	0.580295	-0.2601	0.0306	1.90E-17	-0.000684141	0.000476531	0.16
8	64501744	rs2354862	A	0.648642	-0.2507	0.0317	2.60E-15	0.000774787	0.000496551	0.11
8	120358445	rs2470004	C	0.182928	-0.3454	0.0392	1.24E-18	-0.000466158	0.000607472	0.46
8	26000422	rs2951987	G	0.906472	0.287	0.0515	2.51E-08	-0.000343289	0.000813115	0.71
8	95278307	rs34917849	G	0.872427	0.3124	0.0454	5.94E-12	0.00106526	0.000704002	0.14
8	141057641	rs4440615	G	0.374402	-0.2201	0.0312	1.73E-12	-0.000196885	0.000485689	0.67
8	129483956	rs4598218	C	0.397668	0.1911	0.0313	1.03E-09	-0.0000145753	0.00048675	0.93
8	51947549	rs4873492	C	0.822331	0.3431	0.0403	1.69E-17	-0.000416744	0.000619355	0.49
8	141812374	rs4961293	C	0.540441	0.2268	0.0303	7.15E-14	-0.000286362	0.000474343	0.54
8	82828857	rs62512914	A	0.587525	-0.2066	0.0306	1.46E-11	-0.000481948	0.000478286	0.35
8	1710632	rs6558535	T	0.283012	0.216	0.0336	1.29E-10	-0.000019894	0.000522987	0.97
8	25891653	rs6997996	A	0.742348	-0.4215	0.0347	5.95E-34	-0.000337137	0.000539054	0.52
8	135616959	rs7012866	T	0.492259	0.2325	0.0301	1.13E-14	0.00113337	0.000471277	0.015
8	92777060	rs7830689	G	0.572709	0.1766	0.0306	7.87E-09	-0.0000467183	0.000478179	0.94

8	105921209	rs79069610	T	0.953655	0.4005	0.0727	3.61E-08	-0.00079588	0.00112992	0.51
8	77681093	rs9918879	G	0.897856	-0.2984	0.0499	2.23E-09	-0.00179534	0.000779662	0.021
9	77238558	rs10746963	A	0.189233	0.2177	0.0388	2.01E-08	0.000117934	0.000603292	0.8
9	113249071	rs10980408	T	0.963007	0.7606	0.0827	3.68E-20	0.00240658	0.00125619	0.054
9	139520789	rs11145807	A	0.41863	-0.2135	0.0322	3.35E-11	0.00164351	0.000481784	0.00072
9	9350706	rs1332813	T	0.352021	-0.2203	0.0314	2.28E-12	-0.000949203	0.000493161	0.06
9	116689758	rs2900568	C	0.485825	-0.1889	0.03	3.04E-10	0.000417529	0.000471396	0.38
9	128153616	rs3104552	T	0.55416	-0.2449	0.0303	6.34E-16	-0.0000304785	0.000476362	0.9
9	123516572	rs34025993	A	0.420351	-0.223	0.0308	4.48E-13	0.00135597	0.000479917	0.0056
9	34223553	rs4553000	C	0.487896	-0.2035	0.03	1.17E-11	0.00015342	0.000469855	0.75
9	753648	rs60191654	A	0.813532	0.2382	0.0385	6.13E-10	0.000396653	0.000604352	0.5
9	95201540	rs7045409	T	0.62704	-0.1862	0.0313	2.70E-09	-0.000786633	0.00049128	0.096
9	35906471	rs76452347	C	0.800972	-0.2974	0.0397	6.83E-14	-0.000524588	0.000611739	0.38
9	125863350	rs7854147	A	0.866587	-0.3056	0.0461	3.38E-11	-0.00105296	0.000709376	0.12
9	4117713	rs927315	C	0.526914	0.1689	0.0303	2.49E-08	-0.000595278	0.000474553	0.2
9	22942770	rs9886665	T	0.276372	-0.2048	0.0343	2.36E-09	0.0000246289	0.000534749	0.91
10	102553647	rs1006545	G	0.109493	0.6846	0.048	3.75E-46	0.000217766	0.00075338	0.82
10	82136664	rs10749572	G	0.455856	-0.203	0.0302	1.79E-11	0.000831177	0.000473358	0.085
10	94441507	rs111866816	C	0.930869	0.3569	0.0597	2.26E-09	-0.00139664	0.000933171	0.13
10	104959188	rs11191607	G	0.919336	-1.1036	0.0552	6.37E-89	-0.0014296	0.000864402	0.083
10	4124568	rs11252324	G	0.925092	-0.4164	0.0573	3.68E-13	-0.00188188	0.000892904	0.035
10	134459388	rs1133400	A	0.781647	0.2975	0.0376	2.53E-15	0.00108641	0.000568297	0.057
10	122968964	rs11592107	G	0.693279	0.3024	0.0326	1.76E-20	0.000621439	0.000510868	0.22
10	107158054	rs117464403	G	0.981492	0.864	0.1199	5.76E-13	0.00136025	0.00174556	0.45
10	114808902	rs12255372	G	0.714034	0.2358	0.0335	1.94E-12	0.00025167	0.000519135	0.62

10	32289986	rs12264186	C	0.815399	0.2135	0.0387	3.45E-08	-0.000205471	0.000604605	0.73
10	18471794	rs1623474	C	0.663307	0.3827	0.0321	9.08E-33	-0.000485917	0.000499388	0.34
10	75409877	rs2177843	C	0.846138	0.4394	0.0432	2.66E-24	-0.000854755	0.000658666	0.2
10	64564892	rs2236295	G	0.60618	-0.3028	0.0309	1.13E-22	-0.00149264	0.00048558	0.0019
10	95899706	rs2689690	C	0.638691	-0.2702	0.0316	1.22E-17	-0.000328906	0.00049318	0.48
10	48411796	rs34130368	G	0.884167	-0.3016	0.0497	1.29E-09	-0.000561373	0.000736562	0.46
10	28233469	rs3802517	T	0.545579	0.2527	0.0301	4.65E-17	-0.000300572	0.000471727	0.53
10	60365755	rs4245599	A	0.458314	0.1794	0.0305	4.05E-09	0.000948825	0.000475013	0.05
10	45377839	rs4284362	C	0.281996	-0.2256	0.0338	2.48E-11	0.000959604	0.00053145	0.077
10	63499951	rs57946343	T	0.853804	-0.716	0.0426	2.15E-63	0.000536604	0.000666215	0.4
10	115717311	rs60444686	G	0.959858	0.5905	0.0782	4.31E-14	0.00134542	0.00121149	0.25
10	124234880	rs7093894	C	0.844802	0.236	0.0427	3.26E-08	0.00111325	0.000658803	0.1
10	115792787	rs740746	G	0.269199	0.4557	0.0342	1.67E-40	-0.000385196	0.000532225	0.45
10	133773019	rs7912283	G	0.34722	-0.2144	0.0322	2.77E-11	-0.00089622	0.000493929	0.07
11	30192151	rs10501122	T	0.639856	-0.1916	0.0315	1.18E-09	0.000157817	0.000489627	0.76
11	72088806	rs10501410	G	0.928503	0.4122	0.0607	1.12E-11	0.0000763079	0.000925628	0.91
11	130469044	rs10750441	C	0.328743	0.1754	0.0319	3.83E-08	-0.000525022	0.000501041	0.31
11	130273230	rs11222084	A	0.637038	0.3363	0.0316	1.89E-26	0.000707904	0.00048892	0.16
11	45351729	rs11604357	C	0.834615	-0.277	0.0411	1.59E-11	-0.000526488	0.000635991	0.41
11	27273967	rs1382472	G	0.593052	-0.1917	0.0307	4.26E-10	0.000021293	0.00048017	0.99
11	10679441	rs1544861	T	0.324798	-0.1969	0.0318	5.95E-10	0.00060841	0.000504048	0.21
11	16930289	rs177551	C	0.865509	0.373	0.0442	3.20E-17	0.0000706737	0.00068846	0.9
11	22492454	rs17762	G	0.914614	0.4117	0.0571	5.59E-13	0.00111232	0.000883448	0.2
11	16365282	rs2014408	C	0.789893	0.5169	0.0373	1.14E-43	0.000324568	0.000579015	0.57
11	58407740	rs2276153	C	0.761734	-0.3296	0.0351	5.98E-21	-0.000222415	0.000552472	0.7
11	89224477	rs2289124	G	0.830942	-0.308	0.0415	1.16E-13	-0.000593626	0.000634265	0.32

11	117089628	rs236916	G	0.858803	0.3166	0.0446	1.26E-12	-0.000119485	0.000682393	0.81
11	48109948	rs2904315	A	0.323205	0.2081	0.0325	1.52E-10	-0.00018314	0.000508369	0.72
11	10364963	rs2957688	G	0.531521	0.3472	0.0304	3.28E-30	-0.00021119	0.000471997	0.67
11	1888614	rs4980379	C	0.640392	0.5764	0.032	1.55E-72	-0.000000065945	0.000492395	0.98
11	117267884	rs573455	A	0.468546	-0.1994	0.0303	4.68E-11	-0.000529913	0.000470283	0.25
11	100610546	rs604723	T	0.275115	0.655	0.0339	3.54E-83	0.00118369	0.000531638	0.021
11	100699139	rs629864	C	0.346655	-0.1868	0.0319	4.75E-09	0.00003125	0.000498018	0.92
11	65390803	rs66864335	G	0.778906	-0.3963	0.0365	1.84E-27	0.000555467	0.000566522	0.36
11	47676170	rs7107356	A	0.494947	0.4598	0.0301	1.11E-52	0.0000967192	0.000469068	0.85
11	69825414	rs72931748	A	0.912211	-0.3967	0.0529	6.43E-14	0.00180126	0.000833027	0.036
11	69262916	rs7395791	G	0.556021	-0.2162	0.0308	2.23E-12	0.000978564	0.000473901	0.037
11	2114221	rs74048190	T	0.952147	0.4404	0.0757	5.97E-09	0.00197448	0.00112184	0.074
11	67976593	rs74538877	G	0.945551	-0.3855	0.0707	4.96E-08	0.00190263	0.00106486	0.07
11	107086143	rs7926110	T	0.667503	-0.2603	0.0321	5.10E-16	0.0000742468	0.000501676	0.85
11	28512458	rs871004	G	0.65046	0.2336	0.0317	1.72E-13	0.000189761	0.000494812	0.72
12	20000315	rs1010064	A	0.811403	-0.3571	0.0387	2.77E-20	0.000160892	0.000601954	0.8
12	90349999	rs10777213	G	0.47057	-0.1786	0.0299	2.33E-09	0.000260499	0.000470875	0.6
12	122416254	rs1169078	C	0.682349	0.1971	0.0327	1.66E-09	-0.000576116	0.000505461	0.26
12	133086888	rs117206641	C	0.89377	0.3154	0.0499	2.60E-10	0.000570842	0.000767288	0.46
12	49209340	rs150857355	G	0.978485	0.9406	0.1122	5.15E-17	-0.000870324	0.00164718	0.62
12	90060586	rs17249754	G	0.824506	-0.8446	0.0403	1.59E-97	0.0000404054	0.000619179	0.96
12	115342956	rs1896326	G	0.767909	-0.2797	0.0371	4.73E-14	-0.000680272	0.000570269	0.26
12	26457650	rs2129869	A	0.782654	0.2643	0.0361	2.46E-13	-0.000717627	0.000571366	0.22
12	2436837	rs3819532	T	0.395838	0.1875	0.0306	8.93E-10	0.000523859	0.000481049	0.28
12	67782397	rs4143175	T	0.241043	-0.2187	0.0352	5.20E-10	0.000644212	0.000552038	0.24

12	102837863	rs5742643	T	0.247682	0.2233	0.0349	1.57E-10	0.000414115	0.000541033	0.45
12	48144520	rs61917619	G	0.621432	0.1963	0.0311	2.76E-10	-0.00027999	0.000486234	0.56
12	115920472	rs6490019	A	0.379766	0.2897	0.0309	6.89E-21	0.00135895	0.000485467	0.0046
12	50543201	rs7294548	A	0.363469	-0.3765	0.0308	2.31E-34	0.000928036	0.000489719	0.055
12	66376091	rs7306710	T	0.471967	0.2429	0.0303	1.09E-15	-0.00109936	0.000475543	0.019
12	20373541	rs73075659	A	0.660766	-0.3962	0.0321	5.34E-35	-0.00067214	0.000500369	0.16
12	111865049	rs7310615	C	0.464231	-0.585	0.0306	1.80E-81	-0.00201159	0.000481252	0.000028
12	434755	rs78998485	C	0.744233	0.2449	0.0346	1.46E-12	-0.000101075	0.000538756	0.86
12	79685226	rs7963801	T	0.41767	0.2362	0.0311	3.08E-14	0.000649273	0.00048251	0.19
12	79959658	rs7980644	A	0.164245	-0.2641	0.0404	6.27E-11	-0.000463075	0.000637162	0.49
12	27159784	rs9651825	A	0.73018	0.2042	0.034	1.90E-09	0.00111268	0.000531382	0.037
13	73131694	rs17245822	A	0.631113	0.1899	0.0312	1.15E-09	-0.000594127	0.000491635	0.22
13	41893105	rs2065498	T	0.174312	0.2934	0.0403	3.33E-13	-0.000213789	0.000627549	0.69
13	22294117	rs483071	C	0.372863	0.2709	0.0313	4.93E-18	-0.000608373	0.000493253	0.21
13	74223828	rs6562778	A	0.45629	-0.178	0.0304	4.76E-09	-0.000958239	0.000473801	0.04
13	115059765	rs7320104	A	0.843518	0.4141	0.0427	3.08E-22	0.00046624	0.000653056	0.46
13	47180671	rs7491248	G	0.778637	0.2163	0.0362	2.30E-09	-0.00107002	0.000568423	0.058
13	56398286	rs75961402	G	0.846381	0.2659	0.0418	2.00E-10	0.000133439	0.00065252	0.82
13	73826901	rs78474310	A	0.955908	0.4699	0.0734	1.53E-10	0.00114655	0.00114518	0.32
13	27951090	rs9507885	C	0.910133	-0.3208	0.0542	3.24E-09	-0.000416807	0.00083939	0.63
13	51489186	rs9526707	G	0.682968	-0.2039	0.0323	2.74E-10	-0.000109665	0.00050679	0.8
13	113652369	rs9549627	G	0.884226	0.2846	0.05	1.26E-08	-0.0000813863	0.000740705	0.98
14	75074316	rs11159091	G	0.55513	0.1978	0.0303	6.66E-11	-0.000411056	0.000477496	0.39
14	69259406	rs11847049	C	0.784641	0.2272	0.0364	4.33E-10	-0.0015007	0.000571918	0.0082
14	104007555	rs12885878	A	0.231426	0.2291	0.0367	4.31E-10	0.000532476	0.00057326	0.36

14	100133250	rs17562391	C	0.587279	0.1967	0.0306	1.29E-10	0.000335136	0.000477618	0.52
14	53420358	rs35413927	A	0.698197	0.3002	0.0328	5.57E-20	-0.000698436	0.000512931	0.17
14	23861811	rs365990	A	0.626643	-0.225	0.0312	5.53E-13	0.00119616	0.000486427	0.013
14	68018247	rs57140819	C	0.828186	-0.2415	0.0398	1.30E-09	0.000597034	0.000622018	0.35
14	98590629	rs7154723	G	0.622894	0.253	0.0309	2.66E-16	-0.0000629518	0.000485243	0.91
14	50735947	rs72683923	T	0.981092	-0.9587	0.1101	3.11E-18	-0.00114557	0.00172365	0.49
14	39400917	rs7493678	A	0.652749	0.189	0.0316	2.22E-09	-0.00049389	0.000494999	0.31
14	100197940	rs75016974	C	0.859119	-0.2513	0.0439	1.04E-08	0.000210278	0.000678902	0.76
14	35871217	rs8904	G	0.630679	0.3061	0.0314	1.87E-22	0.00118487	0.000487156	0.015
15	75114322	rs11636952	T	0.32751	-0.5313	0.0328	5.19E-59	-0.000782121	0.000511871	0.13
15	86040872	rs2046341	G	0.807517	-0.2542	0.0382	2.84E-11	-0.000685083	0.000597041	0.28
15	96785017	rs2589218	T	0.729487	0.2258	0.0339	2.72E-11	0.0000556505	0.000530753	0.91
15	81006712	rs2627313	C	0.569389	0.3208	0.0303	3.41E-26	0.00101227	0.000478773	0.037
15	63406170	rs2652812	C	0.238917	-0.2516	0.0353	1.02E-12	-0.00116736	0.000554931	0.03
15	66931617	rs28429256	G	0.66811	0.215	0.0325	3.71E-11	0.000147598	0.000506585	0.75
15	41442195	rs28866311	T	0.520402	0.2762	0.0302	5.92E-20	-0.0016127	0.000470654	0.00072
15	50810621	rs3098186	C	0.481773	-0.2422	0.0303	1.31E-15	0.000167173	0.000470956	0.75
15	100087596	rs4606697	G	0.892925	-0.3196	0.0523	9.91E-10	-0.000704383	0.000759738	0.4
15	48939888	rs4775769	T	0.0916519	0.4162	0.0517	8.26E-16	0.0000934754	0.000816978	0.92
15	91429287	rs4932373	A	0.68104	0.635	0.0328	1.69E-83	0.000976116	0.00050476	0.056
15	90010780	rs77032376	C	0.859885	-0.2727	0.043	2.27E-10	-0.000422549	0.00067956	0.53
15	40314967	rs8030856	C	0.602168	0.1764	0.031	1.27E-08	-0.000576466	0.000484386	0.23
16	74171973	rs1012089	C	0.48485	0.192	0.0302	2.05E-10	0.0000391128	0.000473641	0.87
16	4932929	rs1049212	A	0.442207	0.299	0.0302	4.14E-23	-0.000389787	0.000477387	0.42
16	1347717	rs11641374	C	0.409134	-0.1943	0.0309	3.22E-10	0.000149766	0.000481876	0.74

16	2065666	rs12596630	C	0.906016	0.4278	0.0547	5.25E-15	0.00236144	0.000816732	0.0031
16	81510155	rs12926550	G	0.688141	-0.2548	0.0324	3.71E-15	-0.0000086605 9	0.000509602	0.99
16	66781040	rs146550789	T	0.960829	0.4824	0.0778	5.63E-10	0.000300437	0.00121384	0.82
16	60652439	rs2060664	T	0.751008	-0.216	0.0345	3.83E-10	-0.000131223	0.00054249	0.83
16	4138378	rs2283500	A	0.895426	-0.3106	0.0481	1.07E-10	0.00153804	0.000772319	0.048
16	50550137	rs34941092	G	0.851703	-0.3225	0.0425	3.24E-14	0.000780949	0.000661209	0.24
16	86436343	rs3950627	C	0.482174	0.1851	0.0308	1.86E-09	-0.00131821	0.000479528	0.0075
16	51704452	rs4784541	T	0.484459	0.2015	0.0307	5.26E-11	-0.000656622	0.000479634	0.18
16	75432824	rs4888408	G	0.410141	0.3653	0.0307	1.20E-32	0.000713648	0.000478555	0.13
16	70729954	rs62047964	C	0.939799	0.5115	0.0686	8.90E-14	-0.00109328	0.00103686	0.26
16	87984477	rs6540119	A	0.329418	-0.2016	0.0322	3.83E-10	0.0000433151	0.000505455	0.92
16	21088031	rs7186298	C	0.56843	-0.2315	0.0302	1.78E-14	-0.000204939	0.000473075	0.66
16	3578718	rs72778133	T	0.863307	0.2417	0.0443	4.87E-08	-0.00082837	0.000690553	0.22
16	24811207	rs8044992	T	0.715234	-0.2138	0.0331	1.05E-10	-0.000142843	0.000520273	0.8
16	89697625	rs908951	C	0.573	-0.2261	0.0315	7.09E-13	0.0000927059	0.00047885	0.87
17	59475642	rs1000423	C	0.278995	0.4138	0.0346	5.79E-33	0.000777846	0.00052903	0.14
17	76799795	rs1057040	G	0.483215	-0.2228	0.03	1.11E-13	0.0000698843	0.0004705	0.84
17	7171356	rs113086489	C	0.457581	0.3249	0.0307	3.57E-26	-0.000282447	0.000474818	0.58
17	2012094	rs11653927	C	0.624911	-0.2796	0.0308	1.11E-19	-0.000512782	0.000489613	0.31
17	7870642	rs117285318	T	0.926397	-0.4413	0.0589	6.76E-14	0.000303359	0.000902725	0.74
17	60763400	rs12386061	G	0.584753	0.2188	0.0311	1.99E-12	-0.00051562	0.000479627	0.27
17	75316880	rs1436138	A	0.634728	-0.3119	0.0315	4.10E-23	0.0000924655	0.000490236	0.89
17	30032420	rs1551355	C	0.77487	0.2098	0.0356	3.79E-09	0.0000969114	0.000563479	0.82
17	46123932	rs3764400	T	0.871594	-0.3748	0.0445	3.69E-17	0.00027659	0.000703372	0.65
17	7455536	rs4511593	C	0.351683	-0.2881	0.0318	1.31E-19	-0.000246373	0.000493854	0.62

17	61090958	rs62076622	A	0.805147	-0.2363	0.0377	3.66E-10	-0.000444552	0.000594099	0.47
17	62381714	rs6504213	T	0.404619	0.2982	0.0312	1.20E-21	-0.00119326	0.000480044	0.015
17	19922364	rs7211535	A	0.478904	0.1779	0.0304	4.86E-09	-0.0000428636	0.000471373	0.86
17	43155914	rs7213273	G	0.343629	-0.4	0.0315	6.03E-37	-0.000223414	0.000496526	0.61
17	18221799	rs7221807	T	0.568216	0.217	0.0304	9.46E-13	0.0000446155	0.00047522	0.95
17	1372987	rs9303175	T	0.350597	0.2048	0.0327	3.78E-10	0.00132186	0.000496126	0.0081
17	47518378	rs9897429	G	0.485309	0.2645	0.0319	1.12E-16	-0.000479848	0.000479835	0.34
17	30777924	rs9899540	A	0.394685	-0.2011	0.0316	1.97E-10	0.000294615	0.000486528	0.54
18	54578482	rs10048404	C	0.638009	-0.2607	0.0317	1.97E-16	-0.00022823	0.000489608	0.67
18	73034151	rs10460108	A	0.480684	-0.2141	0.0301	1.14E-12	0.000240411	0.000473063	0.61
18	24546824	rs1154214	T	0.396597	0.2031	0.0306	3.20E-11	0.000716616	0.000481168	0.14
18	42596789	rs11874246	C	0.706494	0.2856	0.0328	3.11E-18	-0.0000380638	0.000515876	0.97
18	48132646	rs1437649	G	0.765908	-0.2189	0.0357	8.70E-10	0.000406858	0.000559346	0.47
18	777282	rs34413141	T	0.810696	-0.3531	0.0393	2.59E-19	0.000418208	0.000603791	0.47
18	42179819	rs56407827	C	0.739595	0.3603	0.034	3.07E-26	0.0000899031	0.000538231	0.83
18	22676071	rs62082230	T	0.725075	-0.1884	0.0345	4.74E-08	-0.000369342	0.000533961	0.5
18	51842682	rs665445	C	0.712476	-0.1909	0.0334	1.09E-08	-0.00144684	0.000524166	0.0067
18	43095231	rs7245140	T	0.811897	0.3367	0.0391	7.23E-18	0.000167847	0.000607071	0.79
19	45298461	rs10420519	G	0.964174	-0.4921	0.0887	2.89E-08	-0.00042912	0.001277	0.77
19	46180184	rs11672660	C	0.808402	0.2212	0.0381	6.41E-09	-0.000619026	0.000595898	0.27
19	5006598	rs12610654	A	0.658243	-0.2315	0.032	4.68E-13	0.000899139	0.000496797	0.066
19	2165383	rs141958336	G	0.960002	0.7807	0.078	1.39E-23	0.00138482	0.00119913	0.24
19	32591878	rs1433121	C	0.298285	-0.228	0.0326	2.67E-12	0.000862765	0.000517256	0.093
19	22111366	rs1848994	G	0.717214	0.2012	0.0334	1.70E-09	0.000118757	0.000521929	0.82
19	11508177	rs2291516	G	0.896944	0.3708	0.0505	2.10E-13	0.000368369	0.000774883	0.64
19	31867447	rs28572357	A	0.600194	0.2733	0.0308	7.10E-19	0.000259379	0.000481672	0.62

19	18455444	rs34518929	G	0.730395	-0.22	0.0345	1.81E-10	-0.000671014	0.000532146	0.21
19	49207554	rs571689	C	0.472378	0.228	0.0304	6.38E-14	0.000163697	0.000473811	0.71
19	45766729	rs7255933	G	0.747374	0.2306	0.0345	2.32E-11	0.000368426	0.000542169	0.46
19	30351998	rs73040775	C	0.934855	-0.3387	0.0619	4.46E-08	-0.0000461062	0.000958077	0.96
19	49605705	rs73046792	G	0.842821	-0.3554	0.0426	7.26E-17	0.000100255	0.000646048	0.92
19	10347032	rs8105174	C	0.81562	-0.2374	0.0398	2.45E-09	0.0000691264	0.000605902	0.89
20	19007099	rs17812022	C	0.907329	-0.3613	0.0525	5.91E-12	-0.000944591	0.000811273	0.26
20	10693337	rs2423514	A	0.539599	-0.3011	0.0302	2.06E-23	-0.00121714	0.000472185	0.0096
20	47241618	rs2598	A	0.516995	-0.168	0.0303	2.95E-08	-0.000687008	0.000475199	0.15
20	51788718	rs2801008	T	0.677404	0.1876	0.0324	7.03E-09	0.00061694	0.000506532	0.22
20	57463472	rs6026578	C	0.375188	0.185	0.0316	4.79E-09	0.000269127	0.000488039	0.59
20	57742388	rs6026744	A	0.881488	0.7131	0.0461	5.66E-54	-0.000400313	0.000730616	0.63
20	40266681	rs6029756	G	0.676721	-0.2712	0.033	2.07E-16	-0.0000612612	0.000509273	0.92
20	6327810	rs6054139	G	0.397826	0.2094	0.0306	7.75E-12	0.000647555	0.000480531	0.17
20	30139886	rs6058088	T	0.84065	-0.2832	0.0417	1.11E-11	0.0005639	0.000667158	0.39
20	11168669	rs6078093	G	0.576711	-0.1849	0.0304	1.19E-09	-0.000347033	0.000479402	0.49
20	47410231	rs6090907	G	0.852358	-0.3854	0.0425	1.21E-19	-0.000493055	0.000663637	0.43
20	10967214	rs6108787	T	0.522859	0.4274	0.03	4.70E-46	-0.000473448	0.00047097	0.3
20	31214944	rs79384779	C	0.851674	0.3179	0.0428	1.11E-13	0.00125174	0.00067299	0.059
20	17883531	rs8125763	C	0.532127	0.1761	0.0301	4.90E-09	-0.000565145	0.000473197	0.24
21	44760603	rs12627651	G	0.723132	0.3498	0.0341	1.09E-24	-0.000287416	0.000525665	0.63
21	16556367	rs1882961	C	0.69885	0.2443	0.0326	6.69E-14	-0.000187183	0.000515671	0.7
21	16317933	rs2776037	T	0.417392	0.1851	0.0309	2.09E-09	-0.0000278556	0.000478719	0.97
21	33814378	rs2833834	C	0.718565	0.2177	0.0338	1.19E-10	0.000531428	0.000525382	0.31
21	44838330	rs34487963	C	0.979599	-0.8819	0.1244	1.35E-12	-0.00237376	0.00168219	0.16
21	44966069	rs7278003	T	0.438511	0.1876	0.0304	6.78E-10	0.00185146	0.000474208	0.0000

										99
22	30135079	rs113264678	C	0.954442	0.4063	0.0727	2.29E-08	-0.0000185122	0.0011256	0.99
22	29453193	rs12321	G	0.562214	-0.2292	0.0303	3.90E-14	-0.000267163	0.000473988	0.59
22	50228044	rs148140538	C	0.914278	-0.3252	0.0562	7.19E-09	0.0012179	0.00084129	0.15
22	19976406	rs2238787	G	0.716137	0.2552	0.0332	1.51E-14	-0.000271447	0.000520815	0.63
22	50727921	rs28578714	T	0.608161	-0.2066	0.0327	2.65E-10	0.0000150788	0.000481904	0.93
22	32001037	rs8142376	C	0.511568	0.1676	0.03	2.31E-08	-0.0000635975	0.000470089	0.93

Abbreviations: SBP: Systolic blood pressure; AR: Allergic rhinitis; EA: effect allele; EAF: effect allele frequency; SE: standard error; SNP: single-nucleotide polymorphism.

Suppl-Table 3 Associations Between Genetically Determined MR analysis of exposures with outcomes, IEU OpenGWAS 2018 (N=462,434).

**Datasets**			**β**	**SE**	**OR (95% CI)**	**P**
Exposure (Smoking)	Outcome	The forward MR				
ukb-b-223	ieu-b-38	MR Egger	11.686	8.159	118862 (0.013-1012)	0.162
		Weighted median	0.065	1.332	1.068 (0.078-14.542)	0.961
		IVW	-4.069	2.329	0.017 (0.001- 1.641)	0.081
		Simple mode	1.894	2.436	6.644 (0.056-787.049)	0.443
		Weighted mode	0.854	1.725	2.348 (0.079-68.999)	0.624
			**β**	**SE**	**OR (95% CI)**	**P**
Exposure(Smoking)	Outcome	The reverse MR				
ukb-b-223	ebi-a-GCST90038664	MR Egger	-0.054	0.056	0.948(0.849-1.057)	0.349
		Weighted median	-0.019	0.019	0.981(0.946-1.018)	0.313
		IVW	-0.026	0.015	0.974(0.946-1.005)	0.099
		Simple mode	-0.016	0.031	0.984(0.927-1.045)	0.611
		Weighted mode	-0.012	0.028	0.987(0.934-1.043)	0.671

Abbreviations: IVW: Inverse variance weighted; CI: confidence interval; OR: odds ratio; SE: standard error; P: P-value

Supplementary Table 4

rsid	trait	chr	pos	p	OR or BET	95% CI (TE	MAPPED_GE	PUBMEDID	FIRST AUTH	JOURNAL	Sample	STUDY ACC	STUDY
rs365990	Heart rate	14	23392602	5e-45	0.564	MYH6	23583979	den Hoed	Nat Genet	85,787 Eu	85,787 Eu	GCST00196	Identifica
rs365990	Electrocar	14	23392602	9e-11	5.25	MYH6	20062063	Holm H	Nat Genet	Up to 12,	Up to 12,	GCST00056	Several co
rs1225896	Blood pres	10	18439030	2e-16	0.431	CACNB2	21909110	Wain LV	Nat Genet	74,064 Eu	74,064 Eu	GCST00123	Genome-wi
rs1724975	Blood pres	12	89666809	1e-17	0.557	ATP2B1	21909110	Wain LV	Nat Genet	74,064 Eu	74,064 Eu	GCST00123	Genome-wi
rs1310732	Blood pres	4	102267552	1e-10	0.633	SLC39A8	21909110	Wain LV	Nat Genet	74,064 Eu	74,064 Eu	GCST00123	Genome-wi
rs1760876	Blood pres	17	46935905	2e-07	0.364	GOSR2	21909110	Wain LV	Nat Genet	74,064 Eu	74,064 Eu	GCST00123	Genome-wi
rs1122208	Blood pres	11	130403335	2e-11	0.337	ZBTB44-DT	21909110	Wain LV	Nat Genet	74,064 Eu	74,064 Eu	GCST00123	Genome-wi
rs1724975	Blood pres	12	89666809	6e-10	0.392	ATP2B1	21909110	Wain LV	Nat Genet	74,064 Eu	74,064 Eu	GCST00123	Genome-wi
rs1760876	Blood pres	17	46935905	6e-15	0.534	GOSR2	21909110	Wain LV	Nat Genet	74,064 Eu	74,064 Eu	GCST00123	Genome-wi
rs1006969	Breast can	5	1279675	5e-12	1.15	TERT	23535733	Garcia-Cl	Nat Genet	4,193 Eur	4,193 Eur	GCST00193	Genome-wi
rs1724975	Biomedical	12	89666809	1e-07	1.06	ATP2B1	19396169	Cho YS	Nat Genet	8,842 Kor	8,842 Kor	GCST00038	A large-sc
rs1724975	Biomedical	12	89666809	3e-06	0.63	ATP2B1	19396169	Cho YS	Nat Genet	8,842 Kor	8,842 Kor	GCST00038	A large-sc
rs1310732	HDL choles	4	102267552	7e-11	0.84	SLC39A8	20686565	Teslovich	Nature	99,900 Eu	99,900 Eu	GCST00075	Biological
rs1052501	Multiple r	3	41883906	2e-08	1.32	ULK4	22120009	Broderick	Nat Genet	1,675 Eur	1,675 Eur	GCST00133	Common var
rs1197752	Insulin-li	7	45968511	3e-101		IGFBP3 -	21216879	Kaplan RC	Hum Mol G	10,280 Eu	10,280 Eu	GCST00093	A genome-w
rs1724975	Blood pres	12	89666809	8e-20	1.17	ATP2B1	21572416	Kato N	Nat Genet	19,608 Ea	19,608 Ea	GCST00107	Meta-analy
rs1724975	Blood pres	12	89666809	2e-13	0.58	ATP2B1	21572416	Kato N	Nat Genet	19,608 Ea	19,608 Ea	GCST00107	Meta-analy
rs1006969	Breast can	5	1279675	7e-09	1.06	TERT	23535729	Michailid	Nat Genet	10,052 Eu	10,052 Eu	GCST00193	Large-scal
rs9356744	Body mass	6	20685255	2e-11	3.39	CDKAL1	22344219	Wen W	Nat Genet	22,762 Ea	22,762 Ea	GCST00141	Meta-analy
rs1724975	Blood pres	12	89666809	7e-15	0.8	ATP2B1	24001895	Kelly TN	Hypertens	22,275 Ea	22,275 Ea	GCST00216	Genome-wi
rs961764	Height	6	117200993	1e-11	0.024	RNA5SP214	20881960	Lango All	Nature	133,653 E	133,653 E	GCST00081	Hundreds o
rs1310732	Systolic b	4	102267552	3e-14	0.981	SLC39A8	21909115	Ehret GB	Nature	69,395 Eu	69,395 Eu	GCST00122	Genetic va
rs1310732	Diastolic	4	102267552	2e-17	0.684	SLC39A8	21909115	Ehret GB	Nature	69,395 Eu	69,395 Eu	GCST00122	Genetic va
rs1760876	Systolic b	17	46935905	1e-10	0.556	GOSR2	21909115	Ehret GB	Nature	69,395 Eu	69,395 Eu	GCST00122	Genetic va
rs1310732	Hypertensi	4	102267552	5e-07	0.105	SLC39A8	21909115	Ehret GB	Nature	69,395 Eu	69,395 Eu	GCST00123	Genetic va
rs1310732	Body mass	4	102267552	2e-13	0.19	SLC39A8	20935630	Speliotes	Nat Genet	Up to 123,	Up to 123,	GCST00083	Associatio
rs3764400	Body mass	17	48046570	4e-07		NFE2L1-DT	20935630	Speliotes	Nat Genet	Up to 123,	Up to 123,	GCST00083	Associatio
rs1006969	Breast can	5	1279675	1e-10	1.18	TERT	22037553	Haiman CA	Nat Genet	1,004 Afr	1,004 Afr	GCST00129	A common v
rs1310732	HDL choles	4	102267552	1e-15	0.071	SLC39A8	24097068	Willer CJ	Nat Genet	94,595 Eu	94,595 Eu	GCST00222	Discovery
rs1006969	Epithelial	5	1279675	9e-09	1.14	TERT	25581431	Kuchenbae	Nat Genet	4,368 Eur	4,368 Eur	GCST00274	Identifica
rs9356744	Fasting pl	6	20685255	9e-10	0.057	CDKAL1	25187374	Hwang JY	Diabetes	24,740 Ea	24,740 Ea	GCST00258	Genome-wi
rs1760876	QRS durati	17	46935905	5e-10	0.53	GOSR2	21076409	Sotoodehn	Nat Genet	39,717 Eu	39,717 Eu	GCST00087	Common var
rs1044822	Metabolite	2	229764422	8e-06	0.2213622	TRIP12	23823483	Rhee EP	Cell Metal	2,076 Eur	2,076 Eur	GCST00939	A genome-w
rs1724975	Diastolic	12	89666809	2e-10	0.52	ATP2B1	25249183	Lu X	Hum Mol G	11,816 Ha	11,816 Ha	GCST00263	Genome-wi
rs1724975	Hypertensi	12	89666809	2e-08		ATP2B1	25249183	Lu X	Hum Mol G	5,065 Han	5,065 Han	GCST00262	Genome-wi
rs1724975	Systolic b	12	89666809	4e-12	1.03	ATP2B1	25249183	Lu X	Hum Mol G	11,816 Ha	11,816 Ha	GCST00263	Genome-wi
rs1006969	Non-gliobl	5	1279675	8e-07	1.25	TERT	26424050	Kinnersle	Nat Commu	2,364 Nor	2,364 Nor	GCST00322	Genome-wi
rs1006969	Glioma	5	1279675	4e-20	1.4	TERT	26424050	Kinnersle	Nat Commu	1,783 Nor	1,783 Nor	GCST00322	Genome-wi
rs1310732	NT-proBNP	4	102267552	6e-10	0.216	SLC39A8	26908625	Johansson	Hum Mol G	at least	at least	GCST00329	Genome-wi
rs1022421	Hemoglobin	7	151716108	3e-12		PRKAG2	28017375	van Rooij	Am J Hum	up to 40,	up to 40,	GCST00400	Genome-wi
rs1122208	Diastolic	11	130403335	1e-12	0.175	ZBTB44-DT	27841878	Hoffmann	Nat Genet	295,529 E	295,529 E	GCST00709	Genome-wi
rs1197752	Diastolic	7	45968511	4e-12	0.166	IGFBP3 -	27841878	Hoffmann	Nat Genet	295,529 E	295,529 E	GCST00709	Genome-wi
rs1250959	Diastolic	4	80261400	2e-61	0.427	PRDM8 - F	27841878	Hoffmann	Nat Genet	295,529 E	295,529 E	GCST00709	Genome-wi
rs740746	Systolic b	10	114033028	5e-13	0.486	NHLRC2 -	27618452	Ehret GB	Nat Genet	up to 201,	up to 201,	GCST00625	The geneti
rs7213273	Systolic b	17	45078546	5e-10	0.413	NMT1	27618452	Ehret GB	Nat Genet	up to 201,	up to 201,	GCST00625	The geneti
rs1760876	Systolic b	17	46935905	2e-15	0.658	GOSR2	27618452	Ehret GB	Nat Genet	up to 201,	up to 201,	GCST00625	The geneti
rs6271	Systolic b	9	133657152	5e-11	0.5911	DBH-AS1,	27618452	Ehret GB	Nat Genet	up to 201,	up to 201,	GCST00625	The geneti
rs1262765	Systolic b	21	43340723	3e-14	0.3905	LINC00322	27618452	Ehret GB	Nat Genet	up to 201,	up to 201,	GCST00625	The geneti
rs2493134	Systolic b	1	230713613	1e-12	0.413	AGT	27618452	Ehret GB	Nat Genet	up to 201,	up to 201,	GCST00625	The geneti
rs7213273	Diastolic	17	45078546	7e-06	0.185	NMT1	27618452	Ehret GB	Nat Genet	up to 201,	up to 201,	GCST00625	The geneti
rs1701095	Systolic b	4	85798012	2e-09	0.498	ARHGAP24	27618452	Ehret GB	Nat Genet	up to 201,	up to 201,	GCST00625	The geneti
rs1310732	Systolic b	4	102267552	5e-11	0.837	SLC39A8	27618452	Ehret GB	Nat Genet	up to 201,	up to 201,	GCST00625	The geneti
rs1265649	Systolic b	5	32831833	4e-16	0.487	NPR3 - LI	27618452	Ehret GB	Nat Genet	up to 201,	up to 201,	GCST00625	The geneti
rs3735533	Systolic b	7	27206274	6e-14	0.798	HOTTIP	27618452	Ehret GB	Nat Genet	up to 201,	up to 201,	GCST00625	The geneti
rs1022421	Hematocrit	7	151716108	1e-08		PRKAG2	28017375	van Rooij	Am J Hum	up to 40,	up to 40,	GCST00400	Genome-wi
rs1290696	Diastolic	15	94768842	1e-10	0.16	LINC02852	27841878	Hoffmann	Nat Genet	295,529 E	295,529 E	GCST00709	Genome-wi
rs1310732	Diastolic	4	102267552	5e-33	0.55	SLC39A8	27841878	Hoffmann	Nat Genet	295,529 E	295,529 E	GCST00709	Genome-wi
rs1342046	Diastolic	2	37290423	1e-06	0.131	PRKD3	27841878	Hoffmann	Nat Genet	295,529 E	295,529 E	GCST00709	Genome-wi
rs1708010	Diastolic	6	150683634	1e-18	0.395	PLEKHG1	27841878	Hoffmann	Nat Genet	295,529 E	295,529 E	GCST00709	Genome-wi
rs7107356	Neurotici	11	47654618	1e-07	0.0180817	MTCH2 - A	27089181	Okbay A	Nat Genet	170,911 E	170,911 E	GCST00377	Genetic va
rs1125098	Schizophre	7	24695385	7e-07	1.09	PALS2 - G	26198764	Goes FS	Am J Med	592 Ashke	592 Ashke	GCST00304	Genome-wi
rs1122208	Pulse pres	11	130403335	4e-06	0.4993	ZBTB44-DT	26390057	Kato N	Nat Genet	31,516 Ea	31,516 Ea	GCST00327	Trans-ance
rs1724975	Systolic b	12	89666809	8e-20	1.0728	ATP2B1	26390057	Kato N	Nat Genet	31,516 Ea	31,516 Ea	GCST00327	Trans-ance
rs604723	Mean arter	11	100739815	2e-12	0.4417	ARHGAP42	26390057	Kato N	Nat Genet	31,516 Ea	31,516 Ea	GCST00327	Trans-ance
rs1724975	Mean arter	12	89666809	2e-16	0.9618	ATP2B1	26390057	Kato N	Nat Genet	31,516 Ea	31,516 Ea	GCST00327	Trans-ance
rs1724975	Pulse pres	12	89666809	3e-09	0.6156	ATP2B1	26390057	Kato N	Nat Genet	31,516 Ea	31,516 Ea	GCST00327	Trans-ance
rs1724975	Systolic b	12	89666809	3e-17	1.3936	ATP2B1	26390057	Kato N	Nat Genet	31,516 Ea	31,516 Ea	GCST00327	Trans-ance
rs1724975	Diastolic	12	89666809	9e-15	0.5308	ATP2B1	26390057	Kato N	Nat Genet	31,516 Ea	31,516 Ea	GCST00327	Trans-ance
rs1724975	Diastolic	12	89666809	5e-13	0.7397	ATP2B1	26390057	Kato N	Nat Genet	31,516 Ea	31,516 Ea	GCST00327	Trans-ance
rs1310732	Childhood	4	102267552	4e-07	0.081	SLC39A8	26604143	Felix JF	Hum Mol G	34,744 Eu	34,744 Eu	GCST00317	Genome-wi
rs1310732	Childhood	4	102267552	1e-08		SLC39A8	26604143	Felix JF	Hum Mol G	34,744 Eu	34,744 Eu	GCST00317	Genome-wi
rs3918226	Coronary a	7	150993088	2e-09	1.14	NOS3	26343387	Nikpay M	Nat Genet	42,096 Eu	42,096 Eu	GCST00311	A comprehe
rs7310615	Fibrinogen	12	111427245	2e-13	0.0069	SH2B3	26561523	de Vries	Hum Mol G	120,246 E	120,246 E	GCST00319	A meta-ana
rs1122208	Pulse pres	11	130403335	8e-69	0.478	ZBTB44-DT	27841878	Hoffmann	Nat Genet	295,529 E	295,529 E	GCST00709	Genome-wi
rs1197752	Pulse pres	7	45968511	9e-50	0.389	IGFBP3 -	27841878	Hoffmann	Nat Genet	295,529 E	295,529 E	GCST00709	Genome-wi
rs1250959	Pulse pres	4	80261400	3e-16	0.23	PRDM8 - F	27841878	Hoffmann	Nat Genet	295,529 E	295,529 E	GCST00709	Genome-wi
rs1760876	Pulse pres	17	46935905	3e-40	0.506	GOSR2	27841878	Hoffmann	Nat Genet	295,529 E	295,529 E	GCST00709	Genome-wi
rs1342046	Pulse pres	2	37290423	5e-08	0.159	PRKD3	27841878	Hoffmann	Nat Genet	295,529 E	295,529 E	GCST00709	Genome-wi
rs2498323	Pulse pres	4	3449382	1e-09	0.29	HGFAC	27841878	Hoffmann	Nat Genet	295,529 E	295,529 E	GCST00709	Genome-wi
rs2643826	Pulse pres	3	27521497	2e-15	0.206	RNU1-96P -	27841878	Hoffmann	Nat Genet	295,529 E	295,529 E	GCST00709	Genome-wi
rs268263	Pulse pres	2	164097664	2e-23	0.289	FIGN - PR	27841878	Hoffmann	Nat Genet	295,529 E	295,529 E	GCST00709	Genome-wi
rs7107356	Systolic b	11	47654618	4e-16	0.309	MTCH2 - A	27841878	Hoffmann	Nat Genet	295,529 E	295,529 E	GCST00709	Genome-wi
rs365990	Resting he	14	23392602	4e-10	0.7840639	MYH6	28270201	Nagy R	Genome Me	19,920 Br	19,920 Br	GCST00421	Explorato
rs1022421	Urea level	7	151716108	6e-09	0.0730428	PRKAG2	28270201	Nagy R	Genome Me	19,293 Br	19,293 Br	GCST00421	Explorato
rs1197752	Systolic b	7	45968511	3e-10	0.248	IGFBP3 -	27841878	Hoffmann	Nat Genet	295,529 E	295,529 E	GCST00709	Genome-wi
rs1250959	Systolic b	4	80261400	9e-55	0.657	PRDM8 - F	27841878	Hoffmann	Nat Genet	295,529 E	295,529 E	GCST00709	Genome-wi
rs8904	Systolic b	14	35402011	4e-11	0.259	NFKBIA	27841878	Hoffmann	Nat Genet	295,529 E	295,529 E	GCST00709	Genome-wi
rs1122208	Systolic b	11	130403335	6e-15	0.317	ZBTB44-DT	27841878	Hoffmann	Nat Genet	295,529 E	295,529 E	GCST00709	Genome-wi
rs1310732	Systolic b	4	102267552	2e-21	0.719	SLC39A8	27841878	Hoffmann	Nat Genet	295,529 E	295,529 E	GCST00709	Genome-wi
rs1342046	Systolic b	2	37290423	1e-10	0.282	PRKD3	27841878	Hoffmann	Nat Genet	295,529 E	295,529 E	GCST00709	Genome-wi
rs1708010	Systolic b	6	150683634	5e-16	0.593	PLEKHG1	27841878	Hoffmann	Nat Genet	295,529 E	295,529 E	GCST00709	Genome-wi
rs1760876	Systolic b	17	46935905	2e-27	0.616	GOSR2	27841878	Hoffmann	Nat Genet	295,529 E	295,529 E	GCST00709	Genome-wi
rs2643826	Systolic b	3	27521497	3e-22	0.376	RNU1-96P -	27841878	Hoffmann	Nat Genet	295,529 E	295,529 E	GCST00709	Genome-wi
rs268263	Systolic b	2	164097664	1e-32	0.517	FIGN - PR	27841878	Hoffmann	Nat Genet	295,529 E	295,529 E	GCST00709	Genome-wi
rs7255933	White bloo	19	45263471	4e-40	0.0543059	MARK4	27863252	Astle WJ	Cell	172,435 E	172,435 E	GCST00461	The Allel
rs3918226	Systolic b	7	150993088	2e-09	0.474	NOS3	27841878	Hoffmann	Nat Genet	295,529 E	295,529 E	GCST00709	Genome-wi
rs6108787	Systolic b	20	10986566	3e-17	0.322	LINC01752	27841878	Hoffmann	Nat Genet	295,529 E	295,529 E	GCST00709	Genome-wi
rs1708010	Diastolic	6	150683634	3e-07	0.394	PLEKHG1	27841878	Hoffmann	Nat Genet	80,792 Eu	80,792 Eu	GCST00709	Genome-wi
rs1708010	Diastolic	6	150683634	1e-06	0.331	PLEKHG1	27841878	Hoffmann	Nat Genet	80,792 Eu	80,792 Eu	GCST00709	Genome-wi
rs2643826	Diastolic	3	27521497	2e-06	0.171	RNU1-96P -	27841878	Hoffmann	Nat Genet	80,792 Eu	80,792 Eu	GCST00709	Genome-wi
rs268263	Diastolic	2	164097664	7e-09	0.232	FIGN - PR	27841878	Hoffmann	Nat Genet	80,792 Eu	80,792 Eu	GCST00709	Genome-wi
rs268263	Diastolic	2	164097664	4e-08	0.244	FIGN - PR	27841878	Hoffmann	Nat Genet	80,792 Eu	80,792 Eu	GCST00709	Genome-wi
[illegible]													
rs2236295	Gout	10	62805132	9e-13	0.9637725	ADO	39406924	Major TJ	Nat Genet	100,671 E	100,671 E	GCST90455	A genome-w
rs7268392	Gout	14	50269229	1e-10	1.152692	L2HGDH	39406924	Major TJ	Nat Genet	100,671 E	100,671 E	GCST90455	A genome-w
rs1078223	Gout	6	125907366	5e-20	1.0466557	NCOA7	39406924	Major TJ	Nat Genet	100,671 E	100,671 E	GCST90455	A genome-w
rs7310615	Edge-level	12	111427245	9e-12	6.8246737	SH2B3	36800424	Sha Z	Sci Adv	30,810 Br	30,810 Br	GCST90165	Genetic ar
rs1006545	Cardioresp	10	100793890	4e-08	0.062	PAX2	34753499	Hanscombe	Genome Me	34,419 Eu	34,419 Eu	GCST90093	The geneti
rs1006545	Cardioresp	10	100793890	6e-12	0.012	PAX2	34753499	Hanscombe	Genome Me	34,419 Eu	34,419 Eu	GCST90093	The geneti
rs1080433	Diastolic	2	226321033	2e-13	0.0125434	NYAP2 - M	39537608	Koskeridi	Nat Commu	at least	at least	GCST90449	Multi-tra
rs2643826	Diastolic	3	27521497	1e-25	0.0177878	RNU1-96P -	39537608	Koskeridi	Nat Commu	at least	at least	GCST90449	Multi-tra
rs3772219	Diastolic	3	56737223	6e-20	0.0166032	ARHGEF3	39537608	Koskeridi	Nat Commu	at least	at least	GCST90449	Multi-tra
rs1094104	Diastolic	5	33194645	7e-11	0.0121595	LINC02120	39537608	Koskeridi	Nat Commu	at least	at least	GCST90449	Multi-tra
rs1664781	Diastolic	5	53980471	4e-11	0.0121368	ARL15	39537608	Koskeridi	Nat Commu	at least	at least	GCST90449	Multi-tra
rs2607015	Waist-to-h	6	31795066	5e-12	0.0196764	VARS1	34021172	Christako	Sci Rep	219,872 B	219,872 B	GCST90020	GWAS of a
rs1006969	Diastolic	5	1279675	7e-14	0.0147928	TERT	39537608	Koskeridi	Nat Commu	at least	at least	GCST90449	Multi-tra
rs1265649	Diastolic	5	32831833	6e-65	0.0294016	NPR3 - LI	39537608	Koskeridi	Nat Commu	at least	at least	GCST90449	Multi-tra
rs2498323	Serum tota	4	3449382	5e-23	0.0412	HGFAC	33462484	Sinnott-A	Nat Genet	313,032 E	313,032 E	GCST90019	Genetics o
rs42032	Serum tota	7	92608112	5e-09	0.0166	CDK6	33462484	Sinnott-A	Nat Genet	313,032 E	313,032 E	GCST90019	Genetics o
rs1006969	Sex hormon	5	1279675	2e-09	0.017	TERT	33462484	Sinnott-A	Nat Genet	310,323 E	310,323 E	GCST90019	Genetics o
rs1871190	Diastolic	5	98618015	2e-08	0.0101016	CTBP2P4 -	39537608	Koskeridi	Nat Commu	at least	at least	GCST90449	Multi-tra
rs6892983	Diastolic	5	128509337	5e-28	0.0189604	FBN2	39537608	Koskeridi	Nat Commu	at least	at least	GCST90449	Multi-tra
rs1196021	Diastolic	5	158390626	7e-41	0.0235666	LINC02227	39537608	Koskeridi	Nat Commu	at least	at least	GCST90449	Multi-tra
rs2744133	Diastolic	6	22392031	4e-13	0.0137466	CASC15	39537608	Koskeridi	Nat Commu	at least	at least	GCST90449	Multi-tra
rs1006969	Serum tota	5	1279675	3e-13	0.0206	TERT	33462484	Sinnott-A	Nat Genet	313,032 E	313,032 E	GCST90019	Genetics o
rs3735533	Diastolic	7	27206274	2e-46	0.0462283	HOTTIP	39537608	Koskeridi	Nat Commu	at least	at least	GCST90449	Multi-tra
rs1906672	Diastolic	8	38272507	2e-11	0.0134095	NSD3, DDH	39537608	Koskeridi	Nat Commu	at least	at least	GCST90449	Multi-tra
rs4873492	Diastolic	8	51034989	2e-09	0.0133999	SNTG1 - P	39537608	Koskeridi	Nat Commu	at least	at least	GCST90449	Multi-tra
rs1098040	Diastolic	9	110486791	2e-14	0.0349697	SVEP1	39537608	Koskeridi	Nat Commu	at least	at least	GCST90449	Multi-tra
rs6271	Diastolic	9	133657152	8e-32	0.0382224	DBH-AS1,	39537608	Koskeridi	Nat Commu	at least	at least	GCST90449	Multi-tra
rs1114580	Diastolic	9	136626337	6e-16	0.0141041	LINC01451	39537608	Koskeridi	Nat Commu	at least	at least	GCST90449	Multi-tra
rs1125232	Diastolic	10	4082376	5e-12	0.0219647	MIR6078 -	39537608	Koskeridi	Nat Commu	at least	at least	GCST90449	Multi-tra
rs2815063	Systolic b	6	39294759	9e-09	0.0145229	KCNK5 - K	39537608	Koskeridi	Nat Commu	at least	at least	GCST90449	Multi-tra
rs7763558	Systolic b	6	43381477	7e-24	0.0183156	ZNF318 -	39537608	Koskeridi	Nat Commu	at least	at least	GCST90449	Multi-tra
rs7864810	Systolic b	6	50715296	1e-14	0.0227739	TFAP2D	39537608	Koskeridi	Nat Commu	at least	at least	GCST90449	Multi-tra
rs1984195	Systolic b	6	78947674	3e-15	0.0134994	PHIP	39537608	Koskeridi	Nat Commu	at least	at least	GCST90449	Multi-tra
rs6921291	Systolic b	6	96618366	6e-19	0.0192661	FHL5	39537608	Koskeridi	Nat Commu	at least	at least	GCST90449	Multi-tra
rs9486916	Systolic b	6	108692727	4e-11	0.014161	FOXO3 - L	39537608	Koskeridi	Nat Commu	at least	at least	GCST90449	Multi-tra
rs1078223	Systolic b	6	125907366	2e-11	0.011432	NCOA7	39537608	Koskeridi	Nat Commu	at least	at least	GCST90449	Multi-tra
rs9401913	Systolic b	6	126838837	1e-62	0.0286587	RPS4XP9 -	39537608	Koskeridi	Nat Commu	at least	at least	GCST90449	Multi-tra
rs2327429	Systolic b	6	133888699	8e-09	0.0108215	TARID	39537608	Koskeridi	Nat Commu	at least	at least	GCST90449	Multi-tra
rs7765526	Systolic b	6	147392628	2e-10	0.010899	STXBP5 -	39537608	Koskeridi	Nat Commu	at least	at least	GCST90449	Multi-tra
rs7555924	Systolic b	6	151839151	2e-08	0.0164591	ESR1	39537608	Koskeridi	Nat Commu	at least	at least	GCST90449	Multi-tra
rs1266103	Systolic b	6	163316444	4e-08	0.0112489	PACRG-AS1	39537608	Koskeridi	Nat Commu	at least	at least	GCST90449	Multi-tra
rs1028212	Systolic b	7	2489989	1e-19	0.016432	GRIFIN -	39537608	Koskeridi	Nat Commu	at least	at least	GCST90449	Multi-tra
rs1310732	A body sha	4	102267552	2e-11	0.0366395	SLC39A8	34021172	Christako	Sci Rep	219,872 B	219,872 B	GCST90020	GWAS of a
rs6248185	Systolic b	7	106771636	1e-73	0.0384994	CCDC71L -	39537608	Koskeridi	Nat Commu	at least	at least	GCST90449	Multi-tra
rs3407272	Systolic b	7	130747710	5e-15	0.0133286	KLF14 - L	39537608	Koskeridi	Nat Commu	at least	at least	GCST90449	Multi-tra
rs7372760	Systolic b	7	149777533	5e-08	0.0188278	SSPOP	39537608	Koskeridi	Nat Commu	at least	at least	GCST90449	Multi-tra
rs3918226	Systolic b	7	150993088	1e-31	0.0366818	NOS3	39537608	Koskeridi	Nat Commu	at least	at least	GCST90449	Multi-tra
rs1022421	Systolic b	7	151716108	5e-28	0.0208413	PRKAG2	39537608	Koskeridi	Nat Commu	at least	at least	GCST90449	Multi-tra
rs5786676	Brain rsFM	10	94263320	1e-43	0.0998702	PLCE1	35393594	Zhao B	Nat Genet	34,691 Br	34,691 Br	GCST90131	Common var
rs5786676	Brain rsFM	10	94263320	5e-44	0.101434	PLCE1	35393594	Zhao B	Nat Genet	34,691 Br	34,691 Br	GCST90131	Common var
rs5786676	Brain rsFM	10	94263320	4e-43	0.103987	PLCE1	35393594	Zhao B	Nat Genet	34,691 Br	34,691 Br	GCST90131	Common var
rs5786676	Brain rsFM	10	94263320	2e-38	0.0966482	PLCE1	35393594	Zhao B	Nat Genet	34,691 Br	34,691 Br	GCST90131	Common var
rs7012866	Systolic b	8	134604716	5e-14	0.0128419	ZFAT	39537608	Koskeridi	Nat Commu	at least	at least	GCST90449	Multi-tra
rs4961293	Systolic b	8	140802275	2e-13	0.0125522	PTK2	39537608	Koskeridi	Nat Commu	at least	at least	GCST90449	Multi-tra
rs6019165	Systolic b	9	753648	2e-09	0.0129979	KANK1 - D	39537608	Koskeridi	Nat Commu	at least	at least	GCST90449	Multi-tra
rs1332813	Systolic b	9	9350706	8e-12	0.0121411	PTPRD	39537608	Koskeridi	Nat Commu	at least	at least	GCST90449	Multi-tra
rs9886665	Systolic b	9	22942771	8e-09	0.0110981	LINC01239	39537608	Koskeridi	Nat Commu	at least	at least	GCST90449	Multi-tra
rs4553000	Systolic b	9	34223555	1e-10	0.0109502	UBAP1	39537608	Koskeridi	Nat Commu	at least	at least	GCST90449	Multi-tra
rs1098040	Systolic b	9	110486791	5e-19	0.0405341	SVEP1	39537608	Koskeridi	Nat Commu	at least	at least	GCST90449	Multi-tra
rs3413036	Systolic b	10	47327566	2e-09	0.015838	GDF2	39537608	Koskeridi	Nat Commu	at least	at least	GCST90449	Multi-tra
rs4245599	Systolic b	10	58605995	4e-08	0.0094027	BICC1	39537608	Koskeridi	Nat Commu	at least	at least	GCST90449	Multi-tra
rs2236295	Systolic b	10	62805132	3e-22	0.0168578	ADO	39537608	Koskeridi	Nat Commu	at least	at least	GCST90449	Multi-tra
rs2177843	Systolic b	10	73650119	1e-22	0.0232764	SYNPO2L-A	39537608	Koskeridi	Nat Commu	at least	at least	GCST90449	Multi-tra
rs7134677	Systolic b	12	54047714	5e-29	0.0207344	HOXC4	39537608	Koskeridi	Nat Commu	at least	at least	GCST90449	Multi-tra
rs7306710	Systolic b	12	65982311	4e-14	0.0128225	HMGA2 - M	39537608	Koskeridi	Nat Commu	at least	at least	GCST90449	Multi-tra
rs4143175	Systolic b	12	67388617	2e-09	0.0119838	MRPL40P1 -	39537608	Koskeridi	Nat Commu	at least	at least	GCST90449	Multi-tra
rs7963801	Systolic b	12	79291446	2e-13	0.0126068	SYT1	39537608	Koskeridi	Nat Commu	at least	at least	GCST90449	Multi-tra
rs573455	Systolic b	11	117397168	6e-09	0.0099504	CEP164	39537608	Koskeridi	Nat Commu	at least	at least	GCST90449	Multi-tra
rs7307565	Systolic b	12	20220607	5e-34	0.021848	LINC02468	39537608	Koskeridi	Nat Commu	at least	at least	GCST90449	Multi-tra
rs4932373	Systolic b	15	90886057	5e-80	0.0347224	FES	39537608	Koskeridi	Nat Commu	at least	at least	GCST90449	Multi-tra
rs1290696	Systolic b	15	94768842	2e-16	0.0149111	LINC02852	39537608	Koskeridi	Nat Commu	at least	at least	GCST90449	Multi-tra
rs1164137	Systolic b	16	1297716	2e-09	0.010383	UBE2I	39537608	Koskeridi	Nat Commu	at least	at least	GCST90449	Multi-tra
rs2498323	Sex hormon	4	3449382	1e-28	0.0465	HGFAC	33462484	Sinnott-A	Nat Genet	310,323 E	310,323 E	GCST90019	Genetics o
rs1111788	Diastolic	1	217563075	3e-19	0.0179435	GPATCH2	39537608	Koskeridi	Nat Commu	at least	at least	GCST90449	Multi-tra
rs1484010	Diastolic	8	80473831	6e-10	0.0283826	RNU6-1213P	39537608	Koskeridi	Nat Commu	at least	at least	GCST90449	Multi-tra
rs3578370	Diastolic	8	104954030	7e-13	0.0199138	ZFPM2	39537608	Koskeridi	Nat Commu	at least	at least	GCST90449	Multi-tra
rs7012866	Diastolic	8	134604716	3e-13	0.0123241	ZFAT	39537608	Koskeridi	Nat Commu	at least	at least	GCST90449	Multi-tra
rs7154723	Systolic b	14	98124292	1e-14	0.0134438	LINC01550	39537608	Koskeridi	Nat Commu	at least	at least	GCST90449	Multi-tra
rs2886631	Systolic b	15	41149997	2e-19	0.0153215	INO80 - C	39537608	Koskeridi	Nat Commu	at least	at least	GCST90449	Multi-tra
rs4775769	Systolic b	15	48647691	4e-13	0.0210111	FBN1-DT	39537608	Koskeridi	Nat Commu	at least	at least	GCST90449	Multi-tra
rs3098186	Systolic b	15	50518424	5e-14	0.012766	USP50	39537608	Koskeridi	Nat Commu	at least	at least	GCST90449	Multi-tra
rs2652812	Systolic b	15	63113971	7e-12	0.0135105	TPM1 - LA	39537608	Koskeridi	Nat Commu	at least	at least	GCST90449	Multi-tra
rs1163695	Systolic b	15	74821981	2e-58	0.0297733	LMAN1L	39537608	Koskeridi	Nat Commu	at least	at least	GCST90449	Multi-tra
rs6562778	Systolic b	13	73649691	2e-08	0.009587	LINC00393	39537608	Koskeridi	Nat Commu	at least	at least	GCST90449	Multi-tra
rs7320104	Systolic b	13	114294290	1e-20	0.0221355	UPF3A	39537608	Koskeridi	Nat Commu	at least	at least	GCST90449	Multi-tra
rs365990	Systolic b	14	23392602	2e-10	0.0112058	MYH6	39537608	Koskeridi	Nat Commu	at least	at least	GCST90449	Multi-tra
rs8904	Systolic b	14	35402011	2e-20	0.0163272	NFKBIA	39537608	Koskeridi	Nat Commu	at least	at least	GCST90449	Multi-tra
rs7268392	Systolic b	14	50269229	3e-17	0.0496684	L2HGDH	39537608	Koskeridi	Nat Commu	at least	at least	GCST90449	Multi-tra
rs3541392	Systolic b	14	52953640	8e-19	0.016294	FERMT2 -	39537608	Koskeridi	Nat Commu	at least	at least	GCST90449	Multi-tra
rs1115909	Systolic b	14	74607613	5e-10	0.0106498	LTBP2	39537608	Koskeridi	Nat Commu	at least	at least	GCST90449	Multi-tra
rs1310732	Waist-to-h	4	102267552	8e-13	0.0387088	SLC39A8	34021172	Christako	Sci Rep	219,872 B	219,872 B	GCST90020	GWAS of a
rs9880058	Systolic b	3	134230522	1e-22	0.0170269	RYK	39537608	Koskeridi	Nat Commu	at least	at least	GCST90449	Multi-tra
rs7953936	Systolic b	3	154962660	1e-14	0.0218962	RPL9P15 -	39537608	Koskeridi	Nat Commu	at least	at least	GCST90449	Multi-tra
rs3980686	Systolic b	3	168979814	2e-23	0.0273693	LINC02082	39537608	Koskeridi	Nat Commu	at least	at least	GCST90449	Multi-tra
rs1290784	Systolic b	3	169379112	4e-41	0.023006	MECOM	39537608	Koskeridi	Nat Commu	at least	at least	GCST90449	Multi-tra
rs262986	Systolic b	3	183717925	8e-14	0.0127314	YEATS2	39537608	Koskeridi	Nat Commu	at least	at least	GCST90449	Multi-tra
rs9869437	Systolic b	3	196501489	1e-09	0.0108861	RNF168	39537608	Koskeridi	Nat Commu	at least	at least	GCST90449	Multi-tra
rs1290933	Systolic b	4	2666490	2e-16	0.0151193	FAM193A	39537608	Koskeridi	Nat Commu	at least	at least	GCST90449	Multi-tra
rs6788984	Systolic b	3	41065682	3e-11	0.0160875	RPS27P4 -	39537608	Koskeridi	Nat Commu	at least	at least	GCST90449	Multi-tra
rs6771917	Systolic b	3	48066952	8e-26	0.0207186	MAP4	39537608	Koskeridi	Nat Commu	at least	at least	GCST90449	Multi-tra
rs7615099	Systolic b	3	53109885	2e-08	0.0101401	RFT1	39537608	Koskeridi	Nat Commu	at least	at least	GCST90449	Multi-tra
rs3772219	Systolic b	3	56737223	8e-17	0.0152458	ARHGEF3	39537608	Koskeridi	Nat Commu	at least	at least	GCST90449	Multi-tra
rs1375564	Systolic b	3	85607161	3e-15	0.0139945	CADM2	39537608	Koskeridi	Nat Commu	at least	at least	GCST90449	Multi-tra

Supplementary Table 5

rsid	trait	chr	pos	p	OR or BET	95% CI (T	MAPPED_GEN	PUBMEDID	FIRST AUTH	JOURNAL	Sample	STUDY ACC	STUDY
rs1898671	Allergic r	5	111072304	5.00E-06	1.15	TSLP	22036096	Ramasamy A	J Allergy	3,933 Eur	3,933 Eur	GCST00131	A genome-w
rs9807989	Asthma	2	102354740	6.00E-08	1.33	IL18R1	22561531	Wan YI	Thorax	933 Europe	933 Europe	GCST00150	Genome-wid
rs2212434	Inflammato	11	76570549	3.00E-12	1.29	EMSY - LIN	25574825	Baurecht H	Am J Hum G	2,079 Eur	2,079 Eur	GCST00274	Genome-wid
rs3421065	Eosinophil	17	4632019	2.00E-42	0.168308	ALOX15	27863252	Astle WJ	Cell	172,275 E	172,275 E	GCST00460	The Alleli
rs3429028	Eosinophil	2	241759225	5.00E-24	0.0412363	D2HGDH	27863252	Astle WJ	Cell	172,275 E	172,275 E	GCST00460	The Alleli
rs3429028	Eosinophil	2	241759225	2.00E-25	0.0424853	D2HGDH	27863252	Astle WJ	Cell	172,378 E	172,378 E	GCST00460	The Alleli
rs3421065	Eosinophil	17	4632019	6.00E-40	0.1630987	ALOX15	27863252	Astle WJ	Cell	172,378 E	172,378 E	GCST00460	The Alleli
rs5637502	Asthma	15	67156025	2.00E-21	1.1148272	SMAD3	27182965	Pickrell J	Nat Genet	28,399 Eu	28,399 Eu	GCST00398	Detection
rs3429028	Asthma	2	241759225	2.00E-15	1.107	D2HGDH	27182965	Pickrell J	Nat Genet	28,399 Eu	28,399 Eu	GCST00398	Detection
rs5743618	Asthma	4	38797027	4.00E-11	1.082	TLR1	27182965	Pickrell J	Nat Genet	28,399 Eu	28,399 Eu	GCST00398	Detection
rs1164451	Allergy	16	11183501	2.00E-16	1.0706638	CLEC16A -	27182965	Pickrell J	Nat Genet	66,973 Eu	66,973 Eu	GCST00399	Detection
rs2074585	Basophil c	15	90466252	4.00E-49		IQGAP1	32888493	Chen MH	Cell	577,663 A	577,663 A	GCST90002	Trans-ethn
rs3429028	Eosinophil	2	241759225	2.00E-70	0.0448912	D2HGDH	32888494	Vuckovic D	Cell	408,112 B	408,112 B	GCST90002	The Polyge
rs3429028	Eosinophil	2	241759225	5.00E-91	0.045886	D2HGDH	32888493	Chen MH	Cell	474,237 E	474,237 E	GCST90002	Trans-ethn
rs3446393	Basophil p	5	35850047	1.00E-10	0.0158994	SPEF2 - II	32888494	Vuckovic D	Cell	408,112 B	408,112 B	GCST90002	The Polyge
rs2074585	Basophil p	15	90466252	2.00E-73	0.040229	IQGAP1	32888494	Vuckovic D	Cell	408,112 B	408,112 B	GCST90002	The Polyge
rs2074585	Basophil c	15	90466252	6.00E-59	0.0359545	IQGAP1	32888494	Vuckovic D	Cell	408,112 B	408,112 B	GCST90002	The Polyge
rs9807989	Eosinophil	2	102354740	9.00E-297	0.07986	IL18R1	32888493	Chen MH	Cell	474,237 E	474,237 E	GCST90002	Trans-ethn
rs1888909	Eosinophil	9	6197392	0	0.095262	GTF3AP1 -	32888493	Chen MH	Cell	474,237 E	474,237 E	GCST90002	Trans-ethn
rs3429028	Eosinophil	2	241759225	6.00E-94		D2HGDH	32888493	Chen MH	Cell	583,850 A	583,850 A	GCST90002	Trans-ethn
rs1888909	Eosinophil	9	6197392	4.00E-229	0.0871512	GTF3AP1 -	32888494	Vuckovic D	Cell	408,112 B	408,112 B	GCST90002	The Polyge
rs9807989	Eosinophil	2	102354740	8.00E-290		IL18R1	30595370	Kichaev G	Am J Hum G	approximat	approximat	GCST00706	Leveraging
rs1236569	Multiple s	11	118872577	2.00E-19	1.1237	Y_RNA - CX	31604244	Internatio	Science	14,802 Eu	14,802 Eu	GCST00959	Multiple s
rs3429028	Eosinophil	2	241759225	4.00E-69	0.0445144	D2HGDH	32888494	Vuckovic D	Cell	408,112 B	408,112 B	GCST90002	The Polyge
rs1888909	Eosinophil	9	6197392	4.00E-255	0.0897564	GTF3AP1 -	32888494	Vuckovic D	Cell	408,112 B	408,112 B	GCST90002	The Polyge
rs3421065	Eosinophil	17	4632019	2.00E-117	0.1784548	ALOX15	32888494	Vuckovic D	Cell	408,112 B	408,112 B	GCST90002	The Polyge
rs9807989	Eosinophil	2	102354740	5.00E-48	0.0464455	IL18R1	32888494	Vuckovic D	Cell	408,112 B	408,112 B	GCST90002	The Polyge
rs1180134	Asthma	19	33235671	9.00E-28		SLC7A10 -	32296059	Han Y	Nat Commun	84,492 Eu	84,492 Eu	GCST01004	Genome-wid
rs2070901	Asthma	1	161215268	7.00E-16		FCER1G	32296059	Han Y	Nat Commun	84,492 Eu	84,492 Eu	GCST01004	Genome-wid
rs5743618	Asthma	4	38797027	4.00E-42		TLR1	32296059	Han Y	Nat Commun	84,492 Eu	84,492 Eu	GCST01004	Genome-wid
rs3429028	Asthma	2	241759225	2.00E-55		D2HGDH	32296059	Han Y	Nat Commun	84,492 Eu	84,492 Eu	GCST01004	Genome-wid
rs3816470	Lymphocyte	17	39829548	8.00E-10	0.033246	IKZF3	32888493	Chen MH	Cell	89,266 Eas	89,266 Eas	GCST90002	Trans-ethn
rs1236569	Asthma	11	118872577	2.00E-18		Y_RNA - CX	32296059	Han Y	Nat Commun	84,492 Eu	84,492 Eu	GCST01004	Genome-wid
rs2197415	Asthma	10	9020893	2.00E-64		LINC02676	32296059	Han Y	Nat Commun	84,492 Eu	84,492 Eu	GCST01004	Genome-wid
rs2070901	Atopic ast	1	161215268	6.00E-09		FCER1G	31669095	Zhu Z	J Allergy	23,982 Eu	23,982 Eu	GCST00985	Shared Gen
rs1236569	Atopic ast	11	118872577	4.00E-11		Y_RNA - CX	31669095	Zhu Z	J Allergy	23,982 Eu	23,982 Eu	GCST00985	Shared Gen
rs3429028	Atopic ast	2	241759225	2.00E-41		D2HGDH	31669095	Zhu Z	J Allergy	23,982 Eu	23,982 Eu	GCST00985	Shared Gen
rs5743618	Allergic r	4	38797027	4.00E-27	1.1111112	TLR1	30013184	Waage J	Nat Genet	59,762 Eu	59,762 Eu	GCST00640	Genome-wid
rs1164451	Allergic r	16	11183501	2.00E-17	1.0752687	CLEC16A -	30013184	Waage J	Nat Genet	59,762 Eu	59,762 Eu	GCST00640	Genome-wid
rs6470578	Allergic r	8	127797311	4.00E-08	1.05	PVT1	30013184	Waage J	Nat Genet	59,762 Eu	59,762 Eu	GCST00640	Genome-wid
rs891058	Allergic s	2	8302417	1.00E-08	1.1363636	LINC00299	30013184	Waage J	Nat Genet	8,040 Eur	8,040 Eur	GCST00640	Genome-wid
rs6470578	Allergic s	8	127797311	9.00E-06	1.11	PVT1	30013184	Waage J	Nat Genet	8,040 Eur	8,040 Eur	GCST00640	Genome-wid
rs3429028	Nonatopic	2	241759225	2.00E-21		D2HGDH	31669095	Zhu Z	J Allergy	28,862 Eu	28,862 Eu	GCST00984	Shared Gen
rs9889953	Atopic ast	17	40598548	2.00E-12		CCR7 - SMA	31669095	Zhu Z	J Allergy	23,982 Eu	23,982 Eu	GCST00985	Shared Gen
rs1180134	Atopic ast	19	33235671	2.00E-14		SLC7A10 -	31669095	Zhu Z	J Allergy	23,982 Eu	23,982 Eu	GCST00985	Shared Gen
rs2070901	Asthma	1	161215268	4.00E-12	1.0477369	FCER1G	32296059	Han Y	Nat Commun	64,538 Eu	64,538 Eu	GCST01004	Genome-wid
rs891058	Asthma	2	8302417	1.00E-24	1.0702096	LINC00299	32296059	Han Y	Nat Commun	64,538 Eu	64,538 Eu	GCST01004	Genome-wid
rs3429028	Asthma	2	241759225	2.00E-70	1.128748	D2HGDH	32296059	Han Y	Nat Commun	64,538 Eu	64,538 Eu	GCST01004	Genome-wid
rs5743618	Asthma	4	38797027	7.00E-43	1.1047432	TLR1	32296059	Han Y	Nat Commun	64,538 Eu	64,538 Eu	GCST01004	Genome-wid
rs5637502	Asthma	15	67156025	5.00E-57	1.1204897	SMAD3	32296059	Han Y	Nat Commun	64,538 Eu	64,538 Eu	GCST01004	Genome-wid
rs2197415	Asthma	10	9020893	6.00E-60	1.1044852	LINC02676	32296059	Han Y	Nat Commun	64,538 Eu	64,538 Eu	GCST01004	Genome-wid
rs1236569	Asthma	11	118872577	2.00E-17	1.0718168	Y_RNA - CX	32296059	Han Y	Nat Commun	64,538 Eu	64,538 Eu	GCST01004	Genome-wid
rs1888909	Chronic rh	9	6197392	6.00E-07	1.12	GTF3AP1 -	30643255	Kristjanss	Nat Genet	251 Europe	251 Europe	GCST00744	A loss-of-
rs3421065	Chronic rh	17	4632019	1.00E-08	1.5625	ALOX15	30643255	Kristjanss	Nat Genet	251 Europe	251 Europe	GCST00744	A loss-of-
rs3421065	Nasal poly	17	4632019	8.00E-27	3.125	ALOX15	30643255	Kristjanss	Nat Genet	86 Europea	86 Europea	GCST00743	A loss-of-
rs1888909	Nasal poly	9	6197392	7.00E-32	1.34	GTF3AP1 -	30643255	Kristjanss	Nat Genet	86 Europea	86 Europea	GCST00743	A loss-of-
rs2070901	Asthma	1	161215268	2.00E-08	1.03959	FCER1G	31619474	Zhu Z	Eur Respir	46,802 Eu	46,802 Eu	GCST00891	Shared Gen
rs1236569	Asthma	11	118872577	9.00E-09	1.0567931	Y_RNA - CX	31619474	Zhu Z	Eur Respir	46,802 Eu	46,802 Eu	GCST00891	Shared Gen
rs3429028	Asthma	2	241759225	4.00E-42	1.1011212	D2HGDH	31619474	Zhu Z	Eur Respir	46,802 Eu	46,802 Eu	GCST00891	Shared Gen
rs2074585	Male-patte	15	90466252	1.00E-37	0.0395814	IQGAP1	30573740	Yap CX	Nat Commun	205,327 E	205,327 E	GCST00702	Dissection
rs2212434	Food aller	11	76570549	9.00E-11	1.29	EMSY - LIN	29051540	Marenholz	Nat Commun	497 Europe	497 Europe	GCST00497	Genome-wid
rs2070901	Aggressive	1	161215268	4.00E-06	1.29	FCER1G	28449029	Munz M	Hum Mol Ge	851 Europe	851 Europe	GCST00430	A genome-w
rs3421065	Eosinophil	17	4632019	8.00E-140	0.175565	ALOX15	32888493	Chen MH	Cell	474,237 E	474,237 E	GCST90002	Trans-ethn
rs3429028	Eosinophil	2	241759225	3.00E-93		D2HGDH	30595370	Kichaev G	Am J Hum G	approximat	approximat	GCST00706	Leveraging
rs1888909	Eosinophil	9	6197392	0		GTF3AP1 -	32888493	Chen MH	Cell	583,850 A	583,850 A	GCST90002	Trans-ethn
rs1236569	Hay fever	11	118872577	3.00E-10	1.05241	Y_RNA - CX	31361310	Johansson	Hum Mol Ge	84,034 Eu	84,034 Eu	GCST00971	Genome-wid
rs3421065	Allergic d	17	4632019	2.00E-10	1.1309658	ALOX15	31361310	Johansson	Hum Mol Ge	106,772 E	106,772 E	GCST00971	Genome-wid
rs1236569	Allergic d	11	118872577	1.00E-09	1.0459157	Y_RNA - CX	31361310	Johansson	Hum Mol Ge	106,772 E	106,772 E	GCST00971	Genome-wid
rs891058	Asthma	2	8302417	2.00E-19		LINC00299	31361310	Johansson	Hum Mol Ge	41,926 Eu	41,926 Eu	GCST00972	Genome-wid
rs3429028	Asthma	2	241759225	1.00E-44		D2HGDH	31361310	Johansson	Hum Mol Ge	41,926 Eu	41,926 Eu	GCST00972	Genome-wid
rs9807989	Eosinophil	2	102354740	6.00E-305		IL18R1	32888493	Chen MH	Cell	583,850 A	583,850 A	GCST90002	Trans-ethn
rs2070901	Hay fever	1	161215268	7.00E-13	1.049	FCER1G	31361310	Johansson	Hum Mol Ge	84,034 Eu	84,034 Eu	GCST00971	Genome-wid
rs763342	Hay fever	3	187921587	4.00E-16	1.052	BCL6-AS1 -	31361310	Johansson	Hum Mol Ge	84,034 Eu	84,034 Eu	GCST00971	Genome-wid
rs3421065	Eosinophil	17	4632019	1.00E-137		ALOX15	32888493	Chen MH	Cell	583,850 A	583,850 A	GCST90002	Trans-ethn
rs2197415	Asthma	10	9020893	4.00E-43		LINC02676	31361310	Johansson	Hum Mol Ge	41,926 Eu	41,926 Eu	GCST00972	Genome-wid
rs1180134	Eosinophil	19	33235671	2.00E-16	0.033602	SLC7A10 -	32888493	Chen MH	Cell	474,237 E	474,237 E	GCST90002	Trans-ethn
rs3429028	Eczema	2	241759225	3.00E-09	1.1244799	D2HGDH	31361310	Johansson	Hum Mol Ge	7,884 Eur	7,884 Eur	GCST00971	Genome-wid
rs3421065	Hay fever	17	4632019	9.00E-10	1.1385632	ALOX15	31361310	Johansson	Hum Mol Ge	84,034 Eu	84,034 Eu	GCST00971	Genome-wid
rs3421065	Allergic r	17	4632019	2.00E-09	1.2914891	ALOX15	31361310	Johansson	Hum Mol Ge	18,915 Eu	18,915 Eu	GCST00971	Genome-wid
rs3429028	Allergic r	2	241759225	3.00E-24	1.1422044	D2HGDH	31361310	Johansson	Hum Mol Ge	18,915 Eu	18,915 Eu	GCST00971	Genome-wid
rs3446393	Allergic r	5	35850047	8.00E-19	1.1184431	SPEF2 - II	31361310	Johansson	Hum Mol Ge	18,915 Eu	18,915 Eu	GCST00971	Genome-wid
rs891058	Allergic r	2	8302417	3.00E-16	1.1071745	LINC00299	31361310	Johansson	Hum Mol Ge	18,915 Eu	18,915 Eu	GCST00971	Genome-wid
rs6094570	Allergic r	20	47053702	5.00E-11	1.0893246	EYA2	31361310	Johansson	Hum Mol Ge	18,915 Eu	18,915 Eu	GCST00971	Genome-wid
rs2988277	Allergic r	1	167462115	3.00E-10	1.0750376	CD247	31361310	Johansson	Hum Mol Ge	18,915 Eu	18,915 Eu	GCST00971	Genome-wid
rs2070901	Allergic r	1	161215268	4.00E-10	1.081	FCER1G	31361310	Johansson	Hum Mol Ge	18,915 Eu	18,915 Eu	GCST00971	Genome-wid
rs3429028	Hay fever	2	241759225	2.00E-51	1.1095084	D2HGDH	31361310	Johansson	Hum Mol Ge	84,034 Eu	84,034 Eu	GCST00971	Genome-wid
rs891058	Hay fever	2	8302417	2.00E-27	1.0738832	LINC00299	31361310	Johansson	Hum Mol Ge	84,034 Eu	84,034 Eu	GCST00971	Genome-wid
rs3429028	Allergic d	2	241759225	6.00E-58	1.1064395	D2HGDH	31361310	Johansson	Hum Mol Ge	106,772 E	106,772 E	GCST00971	Genome-wid
rs891058	Allergic d	2	8302417	3.00E-29	1.069862	LINC00299	31361310	Johansson	Hum Mol Ge	106,772 E	106,772 E	GCST00971	Genome-wid
rs2070901	Allergic d	1	161215268	6.00E-12	1.043	FCER1G	31361310	Johansson	Hum Mol Ge	106,772 E	106,772 E	GCST00971	Genome-wid
rs3421065	Eosinophil	17	4632019	2.00E-117	0.1793041	ALOX15	32888494	Vuckovic D	Cell	408,112 B	408,112 B	GCST90002	The Polyge
rs151234	Mean plate	16	28494339	3.00E-108		CLN3	32888493	Chen MH	Cell	484,042 A	484,042 A	GCST90002	Trans-ethn
rs891058	Respirator	2	8302417	4.00E-18		LINC00299	30595370	Kichaev G	Am J Hum G	approximat	approximat	GCST00707	Leveraging
rs3429028	Respirator	2	241759225	1.00E-39		D2HGDH	30595370	Kichaev G	Am J Hum G	approximat	approximat	GCST00707	Leveraging
rs5743618	Respirator	4	38797027	1.00E-19		TLR1	30595370	Kichaev G	Am J Hum G	approximat	approximat	GCST00707	Leveraging
rs151234	Platelet c	16	28494339	2.00E-111		CLN3	32888493	Chen MH	Cell	721,201 A	721,201 A	GCST90002	Trans-ethn
rs3429028	Asthma (ad	2	241759225	8.00E-20		D2HGDH	31669095	Zhu Z	J Allergy	33,418 Eu	33,418 Eu	GCST00984	Shared Gen
rs3429028	Lung funct	2	241759225	6.00E-09		D2HGDH	30595370	Kichaev G	Am J Hum G	approximat	approximat	GCST00708	Leveraging
rs151234	Platelet c	16	28494339	4.00E-110	0.061846	CLN3	32888493	Chen MH	Cell	542,827 E	542,827 E	GCST90002	Trans-ethn
rs3429028	Eczema	2	241759225	1.00E-58		D2HGDH	30595370	Kichaev G	Am J Hum G	approximat	approximat	GCST00707	Leveraging
rs5743618	Eczema	4	38797027	4.00E-114		TLR1	30595370	Kichaev G	Am J Hum G	approximat	approximat	GCST00707	Leveraging
rs1180134	Eczema	19	33235671	1.00E-19		SLC7A10 -	30595370	Kichaev G	Am J Hum G	approximat	approximat	GCST00707	Leveraging
rs5637502	Eczema	15	67156025	6.00E-32		SMAD3	30595370	Kichaev G	Am J Hum G	approximat	approximat	GCST00707	Leveraging
rs2074585	Eczema	15	90466252	3.00E-22		IQGAP1	30595370	Kichaev G	Am J Hum G	approximat	approximat	GCST00707	Leveraging
rs1236569	Eczema	11	118872577	4.00E-14		Y_RNA - CX	30595370	Kichaev G	Am J Hum G	approximat	approximat	GCST00707	Leveraging
rs2070901	Eczema	1	161215268	1.00E-14		FCER1G	30595370	Kichaev G	Am J Hum G	approximat	approximat	GCST00707	Leveraging
rs891058	Eczema	2	8302417	2.00E-33		LINC00299	30595370	Kichaev G	Am J Hum G	approximat	approximat	GCST00707	Leveraging
rs2197415	Respirator	10	9020893	1.00E-45		LINC02676	30595370	Kichaev G	Am J Hum G	approximat	approximat	GCST00707	Leveraging
rs1236569	Respirator	11	118872577	2.00E-11		Y_RNA - CX	30595370	Kichaev G	Am J Hum G	approximat	approximat	GCST00707	Leveraging
rs3429028	Allergic d	2	241759225	3.00E-13	0.036	D2HGDH	32603359	Ferreira M	PLoS Genet	477,968 E	477,968 E	GCST01098	Age-of-ons
rs3429028	Allergic d	2	241759225	3.00E-13	0.036	D2HGDH	32603359	Ferreira M	PLoS Genet	117,130 E	117,130 E	GCST01098	Age-of-ons
rs2074585	Red cell d	15	90466252	9.00E-13		IQGAP1	30595370	Kichaev G	Am J Hum G	approximat	approximat	GCST00707	Leveraging
rs151234	Mean plate	16	28494339	1.00E-110	0.063986	CLN3	32888493	Chen MH	Cell	460,935 E	460,935 E	GCST90002	Trans-ethn
rs3421065	Neutrophil	17	4632019	3.00E-32	0.1466409	ALOX15	27863252	Astle WJ	Cell	170,672 E	170,672 E	GCST00462	The Alleli
rs891058	Neutrophil	2	8302417	2.00E-17	0.0334707	LINC00299	27863252	Astle WJ	Cell	170,672 E	170,672 E	GCST00462	The Alleli
rs3429028	Neutrophil	2	241759225	3.00E-18	0.0356241	D2HGDH	27863252	Astle WJ	Cell	170,672 E	170,672 E	GCST00462	The Alleli
rs3421065	Eosinophil	17	4632019	3.00E-36	0.1557669	ALOX15	27863252	Astle WJ	Cell	170,536 E	170,536 E	GCST00461	The Alleli
rs2074585	White bloo	15	90466252	2.00E-16	0.0288119	IQGAP1	27863252	Astle WJ	Cell	171,846 E	171,846 E	GCST00461	The Alleli
rs3429028	Eosinophil	2	241759225	3.00E-21	0.0387168	D2HGDH	27863252	Astle WJ	Cell	170,536 E	170,536 E	GCST00461	The Alleli
rs2074585	Basophil p	15	90466252	1.00E-13	0.0259891	IQGAP1	27863252	Astle WJ	Cell	170,223 E	170,223 E	GCST00463	The Alleli
rs151234	Platelet c	16	28494339	1.00E-10	0.0348671	CLN3	27863252	Astle WJ	Cell	164,433 E	164,433 E	GCST00461	The Alleli
rs3421065	Sum eosino	17	4632019	2.00E-39	0.1622121	ALOX15	27863252	Astle WJ	Cell	171,771 E	171,771 E	GCST00462	The Alleli
rs3429028	Sum eosino	2	241759225	5.00E-21	0.0383891	D2HGDH	27863252	Astle WJ	Cell	171,771 E	171,771 E	GCST00462	The Alleli
rs2074585	Basophil p	15	90466252	2.00E-20	0.0321217	IQGAP1	27863252	Astle WJ	Cell	171,996 E	171,996 E	GCST00463	The Alleli
rs1236569	Allergic d	11	118872577	5.00E-18	1.061	Y_RNA - CX	29083406	Ferreira M	Nat Genet	180,129 E	180,129 E	GCST00503	Shared gen
rs5637502	Allergic d	15	67156025	8.00E-32	1.073	SMAD3	29083406	Ferreira M	Nat Genet	180,129 E	180,129 E	GCST00503	Shared gen
rs1164451	Allergic d	16	11183501	6.00E-38	1.072	CLEC16A -	29083406	Ferreira M	Nat Genet	180,129 E	180,129 E	GCST00503	Shared gen
rs2070901	Allergic d	1	161215268	1.00E-11	1.039	FCER1G	29083406	Ferreira M	Nat Genet	180,129 E	180,129 E	GCST00503	Shared gen
rs2988277	Allergic d	1	167462115	4.00E-14	1.04	CD247	29083406	Ferreira M	Nat Genet	180,129 E	180,129 E	GCST00503	Shared gen
rs3429028	Allergic d	2	241759225	4.00E-33	1.082	D2HGDH	29083406	Ferreira M	Nat Genet	180,129 E	180,129 E	GCST00503	Shared gen
rs5743618	Allergic d	4	38797027	3.00E-58	1.1	TLR1	29083406	Ferreira M	Nat Genet	180,129 E	180,129 E	GCST00503	Shared gen
rs2212434	Atopic der	11	76570549	5.00E-13	1.09	EMSY - LIN	26482879	Paternoste	Nat Genet	18,900 Eu	18,900 Eu	GCST00318	Multi-ance
rs3429028	Allergic d	2	241759225	2.00E-15	1.098901	D2HGDH	29785011	Zhu Z	Nat Genet	25,685 Eu	25,685 Eu	GCST00756	A genome-w
rs3429028	Asthma or	2	241759225	5.00E-17	1.0752687	D2HGDH	29785011	Zhu Z	Nat Genet	14,085 Eu	14,085 Eu	GCST00756	A genome-w
rs2074585	Balding ty	15	90466252	3.00E-21		IQGAP1	30595370	Kichaev G	Am J Hum G	approximat	approximat	GCST00703	Leveraging
rs3429028	Asthma (ch	2	241759225	2.00E-34		D2HGDH	31669095	Zhu Z	J Allergy	13,435 Eu	13,435 Eu	GCST00984	Shared Gen
rs2070901	Asthma (ch	1	161215268	3.00E-09		FCER1G	31669095	Zhu Z	J Allergy	13,435 Eu	13,435 Eu	GCST00984	Shared Gen
rs1236569	Asthma (ch	11	118872577	1.00E-14		Y_RNA - CX	31669095	Zhu Z	J Allergy	13,435 Eu	13,435 Eu	GCST00984	Shared Gen
rs3429028	Asthma (mo	2	241759225	2.00E-07	0.8129	D2HGDH	35368043	Chang D	Sci Rep	3,181 Eur	3,181 Eur	GCST90105	A whole ge
rs3441553	Medication	12	56050848	5.00E-17	0.0798495	RPS26 - EF	31015401	Wu Y	Nat Commun	28,880 Eu	28,880 Eu	GCST00794	Genome-wid
rs3429028	Medication	2	241759225	5.00E-28	0.1134818	D2HGDH	31015401	Wu Y	Nat Commun	28,880 Eu	28,880 Eu	GCST00794	Genome-wid
rs3429028	Asthma (ad	2	241759225	2.00E-11	1.081	D2HGDH	31036433	Pividori M	Lancet Res	21,564 Br	21,564 Br	GCST00799	Shared and
rs1236569	Asthma (ag	11	118872577	4.00E-14	1.396	Y_RNA - CX	31036433	Pividori M	Lancet Res	37,846 Br	37,846 Br	GCST00799	Shared and
rs5743618	Asthma (ag	4	38797027	5.00E-22	1.578	TLR1	31036433	Pividori M	Lancet Res	37,846 Br	37,846 Br	GCST00799	Shared and
rs3429028	Asthma (ch	2	241759225	2.00E-20	1.177	D2HGDH	31036433	Pividori M	Lancet Res	9,433 Bri	9,433 Bri	GCST00799	Shared and
rs5743618	Asthma (ch	4	38797027	3.00E-32	1.249	TLR1	31036433	Pividori M	Lancet Res	9,433 Bri	9,433 Bri	GCST00799	Shared and
rs1236569	Asthma (ch	11	118872577	2.00E-13	1.167	Y_RNA - CX	31036433	Pividori M	Lancet Res	9,433 Bri	9,433 Bri	GCST00799	Shared and
rs3429028	Medication	2	241759225	2.00E-23	0.1275955	D2HGDH	31015401	Wu Y	Nat Commun	17,352 Eu	17,352 Eu	GCST00794	Genome-wid
rs1898671	Medication	5	111072304	8.00E-17	0.0975384	TSLP	31015401	Wu Y	Nat Commun	17,352 Eu	17,352 Eu	GCST00794	Genome-wid
rs3429028	Medication	2	241759225	6.00E-09	0.0833708	D2HGDH	31015401	Wu Y	Nat Commun	13,984 Eu	13,984 Eu	GCST00794	Genome-wid
rs5743618	Asthma	4	38797027	4.00E-17	1.0640788	TLR1	31959851	Olafsdotti	Nat Commun	16,247 Eu	16,247 Eu	GCST00979	Eighty-eig
rs1236569	Asthma	11	118872577	4.00E-11	1.058537	Y_RNA - CX	31959851	Olafsdotti	Nat Commun	16,247 Eu	16,247 Eu	GCST00979	Eighty-eig
rs3429028	Asthma	2	241759225	5.00E-36	1.0953622	D2HGDH	31959851	Olafsdotti	Nat Commun	16,247 Eu	16,247 Eu	GCST00979	Eighty-eig
rs2988277	CD3 on CD2	1	167462115	2.00E-71	0.4749	CD247	32929287	Orru V	Nat Genet	2,919 Sar	2,919 Sar	GCST90001	Complex ge
rs2070901	CD45 on gr	1	161215268	6.00E-09	0.173	FCER1G	32929287	Orru V	Nat Genet	3,112 Sar	3,112 Sar	GCST90001	Complex ge
rs3429028	Asthma (mo	2	241759225	2.00E-23	1.1904762	D2HGDH	30552067	Shrine N	Lancet Res	5,135 Eur	5,135 Eur	GCST00691	Moderate-t
rs3816470	Asthma and	17	39829548	3.00E-39		IKZF3	35126453	Zhou Y	Front Gene	19,954 Eu	19,954 Eu	GCST90103	Shared Gen
rs2212434	Ulcerative	11	76570549	2.00E-06	0.041	EMSY - LIN	33686288	Peyrot WJ	Nat Genet	6,968 ulc	6,968 ulc	GCST90016	Identifyin
rs2070901	CD300E/LIL	1	161215268	3.00E-92	0.155694	FCER1G	38412862	Suhre K	Cell Genom	43,509 Eu	43,509 Eu	GCST90313	Genetic as
rs3429028	Atopic der	2	241759225	2.00E-16	0.91	D2HGDH	34116867	Tanaka N	J Allergy	2,597 Japa	2,597 Japa	GCST90086	Eight nove
rs2070901	CLEC4D/MMP	1	161215268	8.00E-249	0.263504	FCER1G	38412862	Suhre K	Cell Genom	43,509 Eu	43,509 Eu	GCST90314	Genetic as
rs2070901	CLEC4D/OSM	1	161215268	7.00E-197	0.232366	FCER1G	38412862	Suhre K	Cell Genom	43,509 Eu	43,509 Eu	GCST90314	Genetic as
rs2070901	CLEC4D/PGL	1	161215268	6.00E-239	0.256973	FCER1G	38412862	Suhre K	Cell Genom	43,509 Eu	43,509 Eu	GCST90314	Genetic as
rs2070901	CLEC4D/RET	1	161215268	1.00E-221	0.247019	FCER1G	38412862	Suhre K	Cell Genom	43,509 Eu	43,509 Eu	GCST90314	Genetic as
rs2070901	CLEC4D/TGF	1	161215268	5.00E-208	0.234204	FCER1G	38412862	Suhre K	Cell Genom	43,509 Eu	43,509 Eu	GCST90314	Genetic as
rs2070901	CLEC4D/HGF	1	161215268	3.00E-226	0.249018	FCER1G	38412862	Suhre K	Cell Genom	43,509 Eu	43,509 Eu	GCST90314	Genetic as
rs2070901	CLEC4D/LCN	1	161215268	6.00E-254	0.270531	FCER1G	38412862	Suhre K	Cell Genom	43,509 Eu	43,509 Eu	GCST90314	Genetic as
rs1236569	Asthma	11	118872577	1.00E-09	0.0535351	Y_RNA - CX	34103634	Valette K	Commun Bio	56,167 Wh	56,167 Wh	GCST90014	Prioritiza
rs5637502	Asthma	15	67156025	3.00E-41	0.103455	SMAD3	34103634	Valette K	Commun Bio	56,167 Wh	56,167 Wh	GCST90014	Prioritiza
rs3429028	Asthma	2	241759225	1.00E-39	0.0988716	D2HGDH	34103634	Valette K	Commun Bio	56,167 Wh	56,167 Wh	GCST90014	Prioritiza
rs5743618	Asthma	4	38797027	3.00E-17	0.0664448	TLR1	34103634	Valette K	Commun Bio	56,167 Wh	56,167 Wh	GCST90014	Prioritiza
rs1888909	Eosinophil	9	6197392	9.00E-154		GTF3AP1 -	34469753	Kachuri L	Am J Hum G	234,763 E	234,763 E	GCST90056	Genetic de
rs3421065	Eosinophil	17	4632019	2.00E-49		ALOX15	34469753	Kachuri L	Am J Hum G	234,763 E	234,763 E	GCST90056	Genetic de
rs3429028	Eosinophil	2	241759225	4.00E-37		D2HGDH	34469753	Kachuri L	Am J Hum G	234,763 E	234,763 E	GCST90056	Genetic de
rs891058	Eczema	2	8302417	3.00E-09	0.93	LINC00299	34785669	Grosche S	Nat Commun	20,016 Eu	20,016 Eu	GCST90044	Rare varia
rs3429028	Eczema	2	241759225	6.00E-09	0.93	D2HGDH	34785669	Grosche S	Nat Commun	20,016 Eu	20,016 Eu	GCST90044	Rare varia
rs3446393	Eczema	5	35850047	1.00E-11	0.92	SPEF2 - II	34785669	Grosche S	Nat Commun	20,016 Eu	20,016 Eu	GCST90044	Rare varia
rs151234	Mean plate	16	28494339	1.00E-73	0.0609062	CLN3	32888494	Vuckovic D	Cell	408,112 B	408,112 B	GCST90002	The Polyge
rs2074585	Male-patte	15	90466252	2.00E-11	0.041139	IQGAP1	28196072	Hagenaars	PLoS Genet	52,874 Br	52,874 Br	GCST00666	Genetic pr
rs5743618	Composite	4	38797027	4.00E-09	0.08	TLR1	28628107	Jonsson S	Nat Genet	14,183 Ic	14,183 Ic	GCST00857	Identifica
rs151234	Inflammato	16	28494339	3.00E-12		CLN3	38965376	Carey CE	Nat Hum Be	337,045 E	337,045 E	GCST90309	Principled
rs3446393	Primary bi	5	35850047	3.00E-08	0.303275	SPEF2 - II	34033851	Cordell HJ	J Hepatol	2,495 East	2,495 East	GCST90061	An interna
rs891058	Primary bi	2	8302417	5.00E-07	0.12017	LINC00299	34033851	Cordell HJ	J Hepatol	8,021 Eur	8,021 Eur	GCST90061	An interna
rs1236569	Respirator	11	118872577	8.00E-14		Y_RNA - CX	38965376	Carey CE	Nat Hum Be	360,406 E	360,406 E	GCST90309	Principled
rs2070901	Respirator	1	161215268	1.00E-12		FCER1G	38965376	Carey CE	Nat Hum Be	360,406 E	360,406 E	GCST90309	Principled
rs5637502	FEV1/FVC r	15	67156025	7.00E-11		SMAD3	38102678	Jiang M	Respir Res	400,102 E	400,102 E	GCST90454	Genetic an
rs2074585	Red cell d	15	90466252	5.00E-12	0.0153069	IQGAP1	32888494	Vuckovic D	Cell	408,112 B	408,112 B	GCST90002	The Polyge
rs151234	Platelet c	16	28494339	1.00E-72	0.0605752	CLN3	32888494	Vuckovic D	Cell	408,112 B	408,112 B	GCST90002	The Polyge
rs2074585	Total test	15	90466252	9.00E-09	0.0160303	IQGAP1	32042192	Ruth KS	Nat Med	230,454 E	230,454 E	GCST90012	Using huma
rs3429028	Asthma (ad	2	241759225	2.00E-28	1.097	D2HGDH	30929738	Ferreira M	Am J Hum G	26,582 Eu	26,582 Eu	GCST00779	Genetic Ar
rs1898671	Asthma (ad	5	111072304	1.00E-22	1.074	TSLP	30929738	Ferreira M	Am J Hum G	26,582 Eu	26,582 Eu	GCST00779	Genetic Ar
rs1898671	Asthma ons	5	111072304	8.00E-10	1.064	TSLP	30929738	Ferreira M	Am J Hum G	13,962 Eu	13,962 Eu	GCST00779	Genetic Ar
rs3429028	Asthma ons	2	241759225	2.00E-08	1.071	D2HGDH	30929738	Ferreira M	Am J Hum G	13,962 Eu	13,962 Eu	GCST00779	Genetic Ar
rs2070901	Asthma ons	1	161215268	1.00E-06	1.054	FCER1G	30929738	Ferreira M	Am J Hum G	13,962 Eu	13,962 Eu	GCST00779	Genetic Ar
rs1236569	Asthma	11	118872577	7.00E-12	1.064	Y_RNA - CX	30929738	Ferreira M	Am J Hum G	40,544 Eu	40,544 Eu	GCST00779	Genetic Ar
rs1236569	Asthma ons	11	118872577	4.00E-12	1.1	Y_RNA - CX	30929738	Ferreira M	Am J Hum G	13,962 Eu	13,962 Eu	GCST00779	Genetic Ar
rs5743618	Asthma ons	4	38797027	3.00E-21	1.114	TLR1	30929738	Ferreira M	Am J Hum G	13,962 Eu	13,962 Eu	GCST00779	Genetic Ar
rs3429028	Asthma	2	241759225	9.00E-52	1.124	D2HGDH	30929738	Ferreira M	Am J Hum G	40,544 Eu	40,544 Eu	GCST00779	Genetic Ar
rs5743618	Asthma	4	38797027	2.00E-31	1.097	TLR1	30929738	Ferreira M	Am J Hum G	40,544 Eu	40,544 Eu	GCST00779	Genetic Ar
rs1898671	Asthma	5	111072304	1.00E-38	1.097	TSLP	30929738	Ferreira M	Am J Hum G	40,544 Eu	40,544 Eu	GCST00779	Genetic Ar
rs2070901	Asthma	1	161215268	9.00E-12	1.054	FCER1G	30929738	Ferreira M	Am J Hum G	40,544 Eu	40,544 Eu	GCST00779	Genetic Ar
rs2070901	Asthma (ch	1	161215268	3.00E-32	1.1	FCER1G	30929738	Ferreira M	Am J Hum G	13,962 Eu	13,962 Eu	GCST00780	Genetic Ar
rs1236569	Asthma (ch	11	118872577	4.00E-30	1.119	Y_RNA - CX	30929738	Ferreira M	Am J Hum G	13,962 Eu	13,962 Eu	GCST00780	Genetic Ar
rs5743618	Asthma (ch	4	38797027	3.00E-74	1.165	TLR1	30929738	Ferreira M	Am J Hum G	13,962 Eu	13,962 Eu	GCST00780	Genetic Ar
rs3429028	Asthma (ch	2	241759225	4.00E-69	1.168	D2HGDH	30929738	Ferreira M	Am J Hum G	13,962 Eu	13,962 Eu	GCST00780	Genetic Ar
rs3421065	Eosinophil	17	4632019	2.00E-41	0.17	ALOX15	35935937	Hoglund J	Front Immu	365,954 B	365,954 B	GCST90134	Gene-Based
rs3429028	Eosinophil	2	241759225	6.00E-15	0.043	D2HGDH	35935937	Hoglund J	Front Immu	365,954 B	365,954 B	GCST90134	Gene-Based
rs1888909	Eosinophil	9	6197392	7.00E-125	0.1	GTF3AP1 -	35935937	Hoglund J	Front Immu	365,954 B	365,954 B	GCST90134	Gene-Based
rs1898671	Eosinophil	5	111072304	2.00E-29	0.043	TSLP	35935937	Hoglund J	Front Immu	365,954 B	365,954 B	GCST90134	Gene-Based
rs5743618	Birth year	4	38797027	3.00E-19		TLR1	35290122	Wu Y	Proc Natl	330,340 E	330,340 E	GCST90095	GWAS on bi
rs2197415	Medication	10	9020893	1.00E-34	0.1048	LINC02676	34594039	Sakaue S	Nat Genet	28,880 Eu	28,880 Eu	GCST90018	A cross-po
rs1898671	Medication	5	111072304	8.00E-20	0.0999	TSLP	34594039	Sakaue S	Nat Genet	17,352 Eu	17,352 Eu	GCST90019	A cross-po
rs3429028	Medication	2	241759225	3.00E-26	0.1125	D2HGDH	34594039	Sakaue S	Nat Genet	17,352 Eu	17,352 Eu	GCST90019	A cross-po
rs3429028	Medication	2	241759225	6.00E-11	0.0781	D2HGDH	34594039	Sakaue S	Nat Genet	13,984 Eu	13,984 Eu	GCST90019	A cross-po
rs2070901	Medication	1	161215268	3.00E-09	0.0531	FCER1G	34594039	Sakaue S	Nat Genet	28,880 Eu	28,880 Eu	GCST90018	A cross-po
rs3429028	Medication	2	241759225	7.00E-33	0.1161	D2HGDH	34594039	Sakaue S	Nat Genet	28,880 Eu	28,880 Eu	GCST90018	A cross-po
rs891058	Medication	2	8302417	2.00E-12	0.0634	LINC00299	34594039	Sakaue S	Nat Genet	28,880 Eu	28,880 Eu	GCST90018	A cross-po
rs3429028	Asthma-COP	2	241759225	4.00E-11	0.89	D2HGDH	35104449	John C	Chest	8,068 Eur	8,068 Eur	GCST90102	Genetic as
rs3421065	eosinophil	17	4632019	2.00E-32	0.104	ALOX15	39024449	Verma A	Science	98,055 Af	98,055 Af	GCST90479	Diversity
rs3429028	eosinophil	2	241759225	3.00E-25	0.03145	D2HGDH	39024449	Verma A	Science	98,055 Af	98,055 Af	GCST90479	Diversity
rs1888909	eosinophil	9	6197392	8.00E-69	0.05199	GTF3AP1 -	39024449	Verma A	Science	98,055 Af	98,055 Af	GCST90479	Diversity
rs3429028	Asthma in	2	241759225	6.00E-10	0.8895852	D2HGDH	36344522	Jia G	Nat Commun	44,383 Eu	44,383 Eu	GCST90244	Discerning
rs3429028	Asthma in	2	241759225	6.00E-12	0.87459	D2HGDH	36344522	Jia G	Nat Commun	44,383 Eu	44,383 Eu	GCST90244	Discerning
rs3429028	Asthma in	2	241759225	1.00E-33	0.902127	D2HGDH	36344522	Jia G	Nat Commun	44,383 Eu	44,383 Eu	GCST90244	Discerning
rs1236569	Asthma in	11	118872577	4.00E-09	0.9427068	Y_RNA - CX	36344522	Jia G	Nat Commun	44,383 Eu	44,383 Eu	GCST90244	Discerning
rs2070901	Asthma	1	161215268	1.00E-17	0.040705	FCER1G	36777996	Zhou W	Cell Genom	139 Middle	139 Middle	GCST90399	Global Bio
rs3429028	Asthma	2	241759225	7.00E-70	0.090878	D2HGDH	36777996	Zhou W	Cell Genom	139 Middle	139 Middle	GCST90399	Global Bio
rs5743618	Asthma	4	38797027	4.00E-21	0.054144	TLR1	36777996	Zhou W	Cell Genom	139 Middle	139 Middle	GCST90399	Global Bio
rs3429028	Age of ons	2	241759225	9.00E-27	0.081912	D2HGDH	36778051	Tsuo K	Cell Genom	56,744 Eu	56,744 Eu	GCST90255	Multi-ance
rs9889953	Atopic der	17	40598548	3.00E-10	0.0528	CCR7 - SMA	38663478	Pasanen A	J Invest D	35,156 Eu	35,156 Eu	GCST90297	Identifyin
rs2212434	ICD10 K50,	11	76570549	4.00E-09	1.12	EMSY - LIN	37965154	Jiang Y	iScience	116,382 E	116,382 E	GCST90432	A cross-di
rs1888909	Takes medi	9	6197392	5.00E-13	0.07927	GTF3AP1 -	39024449	Verma A	Science	4,269 Afr	4,269 Afr	GCST90479	Diversity
rs3429028	Asthma	2	241759225	2.00E-12	0.0689	D2HGDH	39024449	Verma A	Science	5,678 Afr	5,678 Afr	GCST90479	Diversity
rs1888909	Asthma	9	6197392	5.00E-24	0.09777	GTF3AP1 -	39024449	Verma A	Science	5,678 Afr	5,678 Afr	GCST90479	Diversity
rs5637502	Asthma	15	67156025	2.00E-18	0.09207	SMAD3	39024449	Verma A	Science	5,678 Afr	5,678 Afr	GCST90479	Diversity
rs3421065	eosinophil	17	4632019	2.00E-45	0.1468	ALOX15	39024449	Verma A	Science	90,448 Af	90,448 Af	GCST90479	Diversity
rs5637502	eosinophil	15	67156025	1.00E-22	0.04054	SMAD3	39024449	Verma A	Science	90,448 Af	90,448 Af	GCST90479	Diversity
rs2197415	eosinophil	10	9020893	5.00E-28	0.0358	LINC02676	39024449	Verma A	Science	90,448 Af	90,448 Af	GCST90479	Diversity
rs1888909	eosinophil	9	6197392	2.00E-126	0.08301	GTF3AP1 -	39024449	Verma A	Science	90,448 Af	90,448 Af	GCST90479	Diversity
rs3429028	eosinophil	2	241759225	2.00E-37	0.0428	D2HGDH	39024449	Verma A	Science	98,055 Af	98,055 Af	GCST90479	Diversity
rs2212434	Basal cel	11	76570549	1.00E-14	0.06577	EMSY - LIN	39024449	Verma A	Science	31,142 Eu	31,142 Eu	GCST90475	Diversity
rs3421065	eosinophil	17	4632019	3.00E-53	0.1469	ALOX15	39024449	Verma A	Science	98,055 Af	98,055 Af	GCST90479	Diversity
rs1888909	eosinophil	9	6197392	3.00E-130	0.07953	GTF3AP1 -	39024449	Verma A	Science	98,055 Af	98,055 Af	GCST90479	Diversity
rs2197415	eosinophil	10	9020893	1.00E-31	0.03566	LINC02676	39024449	Verma A	Science	98,055 Af	98,055 Af	GCST90479	Diversity
rs3429028	eosinophil	2	241759225	8.00E-36	0.04454	D2HGDH	39024449	Verma A	Science	90,448 Af	90,448 Af	GCST90479	Diversity
rs3429028	eosinophil	2	241759225	6.00E-33	0.04134	D2HGDH	39024449	Verma A	Science	90,403 Af	90,403 Af	GCST90479	Diversity
rs5637502	eosinophil	15	67156025	3.00E-19	0.03598	SMAD3	39024449	Verma A	Science	90,403 Af	90,403 Af	GCST90479	Diversity
rs3421065	eosinophil	17	4632019	2.00E-38	0.1311	ALOX15	39024449	Verma A	Science	90,403 Af	90,403 Af	GCST90479	Diversity
rs1888909	eosinophil	9	6197392	1.00E-116	0.07738	GTF3AP1 -	39024449	Verma A	Science	90,403 Af	90,403 Af	GCST90479	Diversity
rs2197415	eosinophil	10	9020893	1.00E-28	0.03515	LINC02676	39024449	Verma A	Science	90,403 Af	90,403 Af	GCST90479	Diversity
rs3421065	eosinophil	17	4632019	8.00E-28	0.09696	ALOX15	39024449	Verma A	Science	90,479 Af	90,479 Af	GCST90479	Diversity
rs1888909	eosinophil	9	6197392	6.00E-69	0.05105	GTF3AP1 -	39024449	Verma A	Science	90,479 Af	90,479 Af	GCST90479	Diversity
rs3429028	eosinophil	2	241759225	9.00E-20	0.02715	D2HGDH	39024449	Verma A	Science	90,479 Af	90,479 Af	GCST90479	Diversity
rs2197415	eosinophil	10	9020893	4.00E-32	0.03764	LINC02676	39024449	Verma A	Science	98,055 Af	98,055 Af	GCST90479	Diversity
rs3429028	eosinophil	2	241759225	3.00E-44	0.04875	D2HGDH	39024449	Verma A	Science	98,055 Af	98,055 Af	GCST90479	Diversity
rs3421065	eosinophil	17	4632019	3.00E-62	0.1657	ALOX15	39024449	Verma A	Science	98,055 Af	98,055 Af	GCST90479	Diversity
rs1888909	eosinophil	9	6197392	1.00E-147	0.08835	GTF3AP1 -	39024449	Verma A	Science	98,055 Af	98,055 Af	GCST90479	Diversity
rs1888909	Eosinophil	9	6197392	3.00E-09	0.0762913	GTF3AP1 -	34594039	Sakaue S	Nat Genet	93,063 Eas	93,063 Eas	GCST90018	A cross-po
rs2988277	Hypothyroi	1	167462115	2.00E-08	0.0593	CD247	34594039	Sakaue S	Nat Genet	30,155 Eu	30,155 Eu	GCST90018	A cross-po
rs1888909	Nasal poly	9	6197392	1.00E-39	0.3161	GTF3AP1 -	34594039	Sakaue S	Nat Genet	5,093 Eur	5,093 Eur	GCST90018	A cross-po
rs3421065	Nasal poly	17	4632019	2.00E-15	0.6967	ALOX15	34594039	Sakaue S	Nat Genet	5,093 Eur	5,093 Eur	GCST90018	A cross-po
rs3429028	Pediatric	2	241759225	7.00E-23	0.105	D2HGDH	34594039	Sakaue S	Nat Genet	27,712 Eu	27,712 Eu	GCST90018	A cross-po
rs5743618	Pediatric	4	38797027	2.00E-10	0.0717	TLR1	34594039	Sakaue S	Nat Genet	27,712 Eu	27,712 Eu	GCST90018	A cross-po
rs2197415	Pediatric	10	9020893	8.00E-29	0.1046	LINC02676	34594039	Sakaue S	Nat Genet	27,712 Eu	27,712 Eu	GCST90018	A cross-po
rs5743618	Asthma	4	38797027	3.00E-11	0.0664	TLR1	34594039	Sakaue S	Nat Genet	38,369 Eu	38,369 Eu	GCST90018	A cross-po
rs2070901	Asthma	1	161215268	4.00E-10	0.0469	FCER1G	34594039	Sakaue S	Nat Genet	38,369 Eu	38,369 Eu	GCST90018	A cross-po
rs3429028	Asthma	2	241759225	6.00E-37	0.108	D2HGDH	34594039	Sakaue S	Nat Genet	38,369 Eu	38,369 Eu	GCST90018	A cross-po
rs5637502	Inflammato	15	67156025	2.00E-23	0.1168	SMAD3	37156999	Liu Z	Nat Genet	14,393 Eas	14,393 Eas	GCST90292	Genetic ar
rs5637502	Crohn's di	15	67156025	1.00E-20	0.1551	SMAD3	37156999	Liu Z	Nat Genet	7,372 East	7,372 East	GCST90446	Genetic ar
rs5637502	Coronary h	15	67156025	5.00E-08		SMAD3	37947095	Guo Y	J Am Heart	38,687 Eu	38,687 Eu	GCST90446	Genome-Wid
rs3429028	Respirator	2	241759225	5.00E-77		D2HGDH	38965376	Carey CE	Nat Hum Be	360,406 E	360,406 E	GCST90309	Principled
rs5743618	Respirator	4	38797027	4.00E-58		TLR1	38965376	Carey CE	Nat Hum Be	360,406 E	360,406 E	GCST90309	Principled
rs2197415	Respirator	10	9020893	4.00E-49		LINC02676	38965376	Carey CE	Nat Hum Be	360,406 E	360,406 E	GCST90309	Principled
rs891058	Respirator	2	8302417	7.00E-33		LINC00299	38965376	Carey CE	Nat Hum Be	360,406 E	360,406 E	GCST90309	Principled
rs5637502	Ulcerative	15	67156025	2.00E-10	0.0931	SMAD3	37156999	Liu Z	Nat Genet	6,862 East	6,862 East	GCST90446	Genetic ar
rs9807989	Eosinophil	2	102354740	4.00E-190	0.054	IL18R1	34594039	Sakaue S	Nat Genet	349,856 E	349,856 E	GCST90018	A cross-po
rs3429028	Eosinophil	2	241759225	2.00E-60	0.0316	D2HGDH	34594039	Sakaue S	Nat Genet	349,856 E	349,856 E	GCST90018	A cross-po
rs3429028	Asthma	2	241759225	2.00E-15	0.06989	D2HGDH	39024449	Verma A	Science	36,823 Eu	36,823 Eu	GCST90476	Diversity
rs1888909	Allergic r	9	6197392	9.00E-18	0.05207	GTF3AP1 -	39024449	Verma A	Science	92,310 Eu	92,310 Eu	GCST90476	Diversity
rs5637502	Coronary a	15	67156025	3.00E-29	0.0587	SMAD3	39024449	Verma A	Science	124,302 E	124,302 E	GCST90475	Diversity
rs5743618	Rosacea (F	4	38797027	1.00E-16	0.1137	TLR1	39024449	Verma A	Science	14,815 Eu	14,815 Eu	GCST90476	Diversity
rs5743618	high densi	4	38797027	3.00E-12	0.02297	TLR1	39024449	Verma A	Science	404,121 E	404,121 E	GCST90475	Diversity
rs151234	platelet c	16	28494339	1.00E-63	0.06787	CLN3	39024449	Verma A	Science	407,168 E	407,168 E	GCST90476	Diversity
rs3429028	eosinophil	2	241759225	5.00E-25	0.03349	D2HGDH	39024449	Verma A	Science	335,542 E	335,542 E	GCST90475	Diversity
rs3421065	eosinophil	17	4632019	1.00E-25	0.1381	ALOX15	39024449	Verma A	Science	47,410 His	47,410 His	GCST90475	Diversity
rs3429028	eosinophil	2	241759225	4.00E-14	0.04354	D2HGDH	39024449	Verma A	Science	98,055 Af	98,055 Af	GCST90475	Diversity
rs1888909	eosinophil	9	6197392	7.00E-168	0.09279	GTF3AP1 -	39024449	Verma A	Science	335,542 E	335,542 E	GCST90475	Diversity
rs3421065	eosinophil	17	4632019	2.00E-54	0.1615	ALOX15	39024449	Verma A	Science	335,542 E	335,542 E	GCST90475	Diversity
rs3429028	eosinophil	2	241759225	4.00E-43	0.04586	D2HGDH	39024449	Verma A	Science	335,542 E	335,542 E	GCST90475	Diversity
rs1888909	eosinophil	9	6197392	3.00E-207	0.106	GTF3AP1 -	39024449	Verma A	Science	335,542 E	335,542 E	GCST90475	Diversity
rs3429028	eosinophil	2	241759225	2.00E-55	0.05367	D2HGDH	39024449	Verma A	Science	335,542 E	335,542 E	GCST90475	Diversity
rs3429028	eosinophil	2	241759225	2.00E-22	0.03153	D2HGDH	39024449	Verma A	Science	310,849 E	310,849 E	GCST90475	Diversity
rs3429028	eosinophil	2	241759225	1.00E-12	0.04067	D2HGDH	39024449	Verma A	Science	98,055 Af	98,055 Af	GCST90475	Diversity
rs3421065	eosinophil	17	4632019	1.00E-26	0.1423	ALOX15	39024449	Verma A	Science	47,410 His	47,410 His	GCST90475	Diversity
rs3429028	Asthma	2	241759225	3.00E-11	0.06975	D2HGDH	39024449	Verma A	Science	26,657 Eu	26,657 Eu	GCST90475	Diversity
rs3421065	eosinophil	17	4632019	2.00E-43	0.1503	ALOX15	39024449	Verma A	Science	310,588 E	310,588 E	GCST90475	Diversity
rs3429028	eosinophil	2	241759225	1.00E-44	0.04866	D2HGDH	39024449	Verma A	Science	310,588 E	310,588 E	GCST90475	Diversity
rs1888909	eosinophil	9	6197392	4.00E-143	0.08919	GTF3AP1 -	39024449	Verma A	Science	310,588 E	310,588 E	GCST90475	Diversity
rs3421065	eosinophil	17	4632019	1.00E-17	0.1219	ALOX15	39024449	Verma A	Science	42,397 His	42,397 His	GCST90475	Diversity
rs3421065	eosinophil	17	4632019	4.00E-17	0.1181	ALOX15	39024449	Verma A	Science	42,380 His	42,380 His	GCST90475	Diversity
rs3421065	eosinophil	17	4632019	3.00E-58	0.1785	ALOX15	39024449	Verma A	Science	310,757 E	310,757 E	GCST90475	Diversity
rs3429028	eosinophil	2	241759225	8.00E-51	0.05314	D2HGDH	39024449	Verma A	Science	310,757 E	310,757 E	GCST90475	Diversity
rs1494018	Total test	2	197626617	3.00E-09	0.0201597	RFTN2	36653534	Leinonen J	Commun Med	174,850 B	174,850 B	GCST90239	Genetic an
rs891058	Atopic der	2	8302417	3.00E-18	0.97	LINC00299	37794016	Budu-Aggre	Nat Commun	60,653 Eu	60,653 Eu	GCST90244	European a
rs151234	Platelet l	16	28494339	1.00E-17	0.0901256	CLN3	37596262	Akbari P	Nat Commun	37,935 Eu	37,935 Eu	GCST90281	A genome-w
rs151234	Platelet s	16	28494339	3.00E-15	0.0979563	CLN3	37596262	Akbari P	Nat Commun	29,675 Eu	29,675 Eu	GCST90281	A genome-w
rs5743618	White bloo	4	38797027	8.00E-103		TLR1	38049662	Homilius M	Nat Genet	1,400 Eur	1,400 Eur	GCST90257	Perturbati
rs151234	High fluor	16	28494339	6.00E-33	0.128122	CLN3	37596262	Akbari P	Nat Commun	36,829 Eu	36,829 Eu	GCST90281	A genome-w
rs151234	Immature p	16	28494339	2.00E-20	0.0994912	CLN3	37596262	Akbari P	Nat Commun	36,618 Eu	36,618 Eu	GCST90281	A genome-w
rs151234	Platelet f	16	28494339	2.00E-17	0.10184	CLN3	37596262	Akbari P	Nat Commun	29,662 Eu	29,662 Eu	GCST90281	A genome-w
rs151234	Platelet s	16	28494339	2.00E-18	0.105604	CLN3	37596262	Akbari P	Nat Commun	29,678 Eu	29,678 Eu	GCST90281	A genome-w
rs151234	Immature p	16	28494339	3.00E-28	0.118148	CLN3	37596262	Akbari P	Nat Commun	36,615 Eu	36,615 Eu	GCST90281	A genome-w
rs2070901	Age of ons	1	161215268	2.00E-08	0.066391	FCER1G	36778051	Tsuo K	Cell Genom	20,964 Eu	20,964 Eu	GCST90255	Multi-ance
rs3429028	Age of ons	2	241759225	1.00E-34	0.15369	D2HGDH	36778051	Tsuo K	Cell Genom	20,964 Eu	20,964 Eu	GCST90255	Multi-ance
rs5743618	Turnip lik	4	38797027	7.00E-10	0.0261	TLR1	35585065	May-Wilson	Nat Commun	155,376 E	155,376 E	GCST90094	Large-scal
rs2074585	Testostero	15	90466252	2.00E-07	0.0170713	IQGAP1	35192695	Haas CB	Endocrinol	182,648 E	182,648 E	GCST90104	Cross-ance
rs5743618	Age of ons	4	38797027	5.00E-36	0.1707	TLR1	36778051	Tsuo K	Cell Genom	20,964 Eu	20,964 Eu	GCST90255	Multi-ance
rs763342	Hemoglobin	3	187921587	1.00E-06	0.01919	BCL6-AS1 -	35964923	Timoteo VJ	J Nutr Bio	46,904 Eu	46,904 Eu	GCST90258	Common and
rs3816470	Sinonasal	17	39829548	3.00E-07	0.95	IKZF3	36653354	Saarentaus	Nat Commun	25,235 Fi	25,235 Fi	GCST90269	Inflammato
rs1164451	Vasomotor	16	11183501	1.00E-08	0.99	CLEC16A -	36653354	Saarentaus	Nat Commun	at least 8	at least 8	GCST90269	Inflammato
rs1164451	Chronic ir	16	11183501	1.00E-08	0.93	CLEC16A -	36653354	Saarentaus	Nat Commun	19,901 Fi	19,901 Fi	GCST90269	Inflammato
rs3816470	Chronic ir	17	39829548	2.00E-08	0.94	IKZF3	36653354	Saarentaus	Nat Commun	19,901 Fi	19,901 Fi	GCST90269	Inflammato
rs5743618	Toll-like	4	38797027	5.00E-14	0.113	TLR1	34648354	Pietzner M	Science	10,708 Eu	10,708 Eu	GCST90249	Mapping th
rs2212434	Basal cel	11	76570549	2.00E-11	0.06599	EMSY - LIN	39024449	Verma A	Science	768 Hispan	768 Hispan	GCST90479	Diversity
rs1236569	Non-Hodgki	11	118872577	2.00E-11	0.1556	Y_RNA - CX	39024449	Verma A	Science	1,038 Afr	1,038 Afr	GCST90479	Diversity
rs1236569	Cancer of	11	118872577	4.00E-11	0.1488	Y_RNA - CX	39024449	Verma A	Science	1,108 Afr	1,108 Afr	GCST90479	Diversity
rs151234	mean plate	16	28494339	5.00E-44	0.07997	CLN3	39024449	Verma A	Science	97,007 Af	97,007 Af	GCST90479	Diversity
rs151234	mean plate	16	28494339	3.00E-42	0.07687	CLN3	39024449	Verma A	Science	97,007 Af	97,007 Af	GCST90479	Diversity
rs151234	mean plate	16	28494339	3.00E-38	0.07127	CLN3	39024449	Verma A	Science	97,007 Af	97,007 Af	GCST90479	Diversity
rs2074585	Testostero	15	90466252	2.00E-07	5.164	IQGAP1	35192695	Haas CB	Endocrinol	4,229 Afr	4,229 Afr	GCST90104	Cross-ance
rs5743618	Rosacea (F	4	38797027	6.00E-17	0.1164	TLR1	39024449	Verma A	Science	410 Africa	410 Africa	GCST90480	Diversity
rs151234	platelet c	16	28494339	4.00E-38	0.06334	CLN3	39024449	Verma A	Science	114,799 A	114,799 A	GCST90480	Diversity
rs1888909	Biological	9	6197392	9.00E-13	0.07644	GTF3AP1 -	39024449	Verma A	Science	5,022 Afr	5,022 Afr	GCST90480	Diversity
rs1888909	Nasal poly	9	6197392	2.00E-28	0.2467	GTF3AP1 -	39024449	Verma A	Science	905 Africa	905 Africa	GCST90480	Diversity
rs3429028	Asthma	2	241759225	2.00E-22	0.0786	D2HGDH	39024449	Verma A	Science	12,093 Af	12,093 Af	GCST90480	Diversity
rs1888909	Asthma	9	6197392	5.00E-31	0.08906	GTF3AP1 -	39024449	Verma A	Science	12,093 Af	12,093 Af	GCST90480	Diversity
rs151234	platelet c	16	28494339	7.00E-32	0.05942	CLN3	39024449	Verma A	Science	114,580 A	114,580 A	GCST90480	Diversity
rs151234	platelet c	16	28494339	3.00E-41	0.07067	CLN3	39024449	Verma A	Science	114,731 A	114,731 A	GCST90480	Diversity
rs6094570	Height	20	47053702	2.00E-08	0.0042	EYA2	36224396	Yengo L	Nature	5,314,291	5,314,291	GCST90245	A saturate

Supplementary Table 6

	SNP	chr. outcor	pos. outcor	effect_al	other_all	eaf. outcor	beta. outc	se. outcom	pval. outc	outcome	mr_keep. o	pval_orig	id. outcome
102	rs1310732	4	103188709	C	T	0.92814	-0.004779	0.0009113	1.60E-07	outcome	TRUE	reported	FVRUio
394	rs7864810	6	50683009	T	C	0.915307	-0.002895	0.0008432	0.00067	outcome	TRUE	reported	FVRUio
284	rs5998083	1	115827266	G	T	0.981267	-0.002533	0.0017372	0.13	outcome	TRUE	reported	FVRUio
225	rs3448796	21	44838330	C	A	0.979599	-0.002374	0.0016822	0.16	outcome	TRUE	reported	FVRUio
360	rs7310615	12	111865049	C	G	0.464231	-0.002012	0.0004813	2.80E-05	outcome	TRUE	reported	FVRUio
44	rs1125232	10	4124568	G	T	0.925092	-0.001882	0.0008929	0.035	outcome	TRUE	reported	FVRUio
436	rs9918879	8	77681093	G	T	0.897856	-0.001795	0.0007797	0.021	outcome	TRUE	reported	FVRUio
277	rs5594433	2	145726621	A	G	0.76984	-0.001731	0.0005612	0.0022	outcome	TRUE	reported	FVRUio
330	rs6932812	6	166180947	C	G	0.923889	-0.001652	0.0009006	0.074	outcome	TRUE	reported	FVRUio
211	rs2886631	15	41442195	T	G	0.520402	-0.001613	0.0004707	0.00072	outcome	TRUE	reported	FVRUio
382	rs7671927	1	156129796	C	T	0.873915	-0.001607	0.000718	0.025	outcome	TRUE	reported	FVRUio
61	rs1184704	14	69259406	C	G	0.784641	-0.001501	0.0005719	0.0082	outcome	TRUE	reported	FVRUio
166	rs2236295	10	64564892	G	T	0.60618	-0.001493	0.0004856	0.0019	outcome	TRUE	reported	FVRUio
317	rs665445	18	51842682	C	A	0.712476	-0.001447	0.0005242	0.0067	outcome	TRUE	reported	FVRUio
39	rs1119160	10	104959188	G	T	0.919336	-0.00143	0.0008644	0.083	outcome	TRUE	reported	FVRUio
38	rs1118668	10	94441507	C	T	0.930869	-0.001397	0.0009332	0.13	outcome	TRUE	reported	FVRUio
73	rs1215339	5	179411477	G	A	0.889049	-0.001328	0.0007561	0.084	outcome	TRUE	reported	FVRUio
243	rs3950627	16	86436343	C	A	0.482174	-0.001318	0.0004795	0.0075	outcome	TRUE	reported	FVRUio
72	rs1213705	1	28707612	C	T	0.689976	-0.001255	0.0005099	0.016	outcome	TRUE	reported	FVRUio
431	rs9876694	3	141152017	C	T	0.935221	-0.001244	0.0009734	0.2	outcome	TRUE	reported	FVRUio
180	rs2423514	20	10693337	A	G	0.539599	-0.001217	0.0004722	0.0096	outcome	TRUE	reported	FVRUio
312	rs6504213	17	62381714	T	C	0.404619	-0.001193	0.00048	0.015	outcome	TRUE	reported	FVRUio
194	rs2652812	15	63406170	C	T	0.238917	-0.001167	0.0005549	0.03	outcome	TRUE	reported	FVRUio
421	rs9401913	6	127159982	G	A	0.568279	-0.001155	0.0004747	0.013	outcome	TRUE	reported	FVRUio
81	rs1252897	6	30988017	G	A	0.950173	-0.001155	0.0010772	0.28	outcome	TRUE	reported	FVRUio
347	rs7268392	14	50735947	T	C	0.981092	-0.001146	0.0017237	0.49	outcome	TRUE	reported	FVRUio
418	rs9327297	5	122835051	C	G	0.671138	-0.001142	0.0005002	0.021	outcome	TRUE	reported	FVRUio
132	rs1708010	6	151004770	G	C	0.926348	-0.001117	0.0009003	0.22	outcome	TRUE	reported	FVRUio
357	rs7306710	12	66376091	T	C	0.471967	-0.001099	0.0004755	0.019	outcome	TRUE	reported	FVRUio
300	rs6204796	16	70729954	C	T	0.939799	-0.001093	0.0010369	0.26	outcome	TRUE	reported	FVRUio
147	rs1870735	7	155744303	C	G	0.451641	-0.001093	0.0004768	0.021	outcome	TRUE	reported	FVRUio
97	rs1290933	4	2668217	C	A	0.320046	-0.001082	0.0005077	0.033	outcome	TRUE	reported	FVRUio
143	rs1786345	8	101674751	A	C	0.556991	-0.001072	0.0004756	0.031	outcome	TRUE	reported	FVRUio
369	rs7491248	13	47180671	G	A	0.778637	-0.00107	0.0005684	0.058	outcome	TRUE	reported	FVRUio
393	rs7854147	9	125863350	A	G	0.866587	-0.001053	0.0007094	0.12	outcome	TRUE	reported	FVRUio
165	rs2232460	1	6659505	G	A	0.66458	-0.001048	0.0004976	0.036	outcome	TRUE	reported	FVRUio
68	rs1204292	1	197297417	T	C	0.538043	-0.000991	0.0004742	0.035	outcome	TRUE	reported	FVRUio
315	rs6562778	13	74223828	A	G	0.45629	-0.000958	0.0004738	0.04	outcome	TRUE	reported	FVRUio
105	rs1332813	9	9350706	T	C	0.352021	-0.000949	0.0004932	0.06	outcome	TRUE	reported	FVRUio
142	rs1781202	20	19007099	C	T	0.907329	-0.000945	0.0008113	0.26	outcome	TRUE	reported	FVRUio
88	rs1265795	5	61940569	C	T	0.93318	-0.000902	0.0009392	0.32	outcome	TRUE	reported	FVRUio
398	rs7912283	10	133773019	G	A	0.34722	-0.000896	0.0004939	0.07	outcome	TRUE	reported	FVRUio
63	rs1192550	3	14943965	G	A	0.426906	-0.000885	0.0004753	0.058	outcome	TRUE	reported	FVRUio
131	rs1703518	4	157678511	T	G	0.852965	-0.000876	0.0006642	0.2	outcome	TRUE	reported	FVRUio
145	rs1821002	8	10640065	C	G	0.401949	-0.000873	0.0004804	0.084	outcome	TRUE	reported	FVRUio
122	rs1508573	12	49209340	G	C	0.978485	-0.00087	0.0016472	0.62	outcome	TRUE	reported	FVRUio
164	rs2177843	10	75409877	C	T	0.846138	-0.000855	0.0006587	0.2	outcome	TRUE	reported	FVRUio
65	rs1196726	6	43760327	C	G	0.513025	-0.000849	0.0004727	0.068	outcome	TRUE	reported	FVRUio
426	rs961764	6	117522156	C	G	0.431956	-0.000838	0.0004755	0.089	outcome	TRUE	reported	FVRUio
349	rs7277813	16	3578718	T	C	0.863307	-0.000828	0.0006906	0.22	outcome	TRUE	reported	FVRUio
387	rs7763558	6	43349215	G	A	0.668886	-0.000827	0.0005019	0.095	outcome	TRUE	reported	FVRUio
386	rs7725413	5	15695987	C	T	0.232201	-0.000808	0.0005583	0.15	outcome	TRUE	reported	FVRUio
397	rs7906961	8	105921209	T	C	0.953655	-0.000796	0.0011299	0.51	outcome	TRUE	reported	FVRUio
128	rs1630736	6	12295987	C	T	0.543982	-0.000791	0.0004768	0.11	outcome	TRUE	reported	FVRUio
336	rs7045409	9	95201540	T	A	0.62704	-0.000787	0.0004913	0.096	outcome	TRUE	reported	FVRUio
53	rs1163695	15	75114322	T	C	0.32751	-0.000782	0.0005119	0.13	outcome	TRUE	reported	FVRUio
275	rs5020545	4	77414988	C	T	0.564266	-0.000748	0.0004772	0.12	outcome	TRUE	reported	FVRUio
172	rs2291434	4	38387244	G	T	0.467921	-0.000748	0.0004716	0.11	outcome	TRUE	reported	FVRUio
174	rs2294214	6	22056923	A	C	0.690639	-0.000719	0.0005084	0.14	outcome	TRUE	reported	FVRUio
162	rs2129869	12	26457650	A	T	0.782654	-0.000718	0.0005714	0.22	outcome	TRUE	reported	FVRUio
256	rs4606697	15	100087596	G	A	0.892925	-0.000704	0.0007597	0.4	outcome	TRUE	reported	FVRUio
231	rs3541392	14	53420358	A	G	0.698197	-0.000698	0.0005129	0.17	outcome	TRUE	reported	FVRUio
188	rs2598	20	47241618	A	G	0.516995	-0.000687	0.0004752	0.15	outcome	TRUE	reported	FVRUio
43	rs1125098	7	24735004	G	C	0.884645	-0.000687	0.0007418	0.38	outcome	TRUE	reported	FVRUio
157	rs2046341	15	86040872	G	A	0.807517	-0.000685	0.000597	0.28	outcome	TRUE	reported	FVRUio
161	rs2126474	8	76878957	G	T	0.580295	-0.000684	0.0004765	0.16	outcome	TRUE	reported	FVRUio
152	rs1896326	12	115342956	G	A	0.767909	-0.00068	0.0005703	0.26	outcome	TRUE	reported	FVRUio
358	rs7307565	12	20373541	A	G	0.660766	-0.000672	0.0005004	0.16	outcome	TRUE	reported	FVRUio
226	rs3451892	19	18455444	G	A	0.730395	-0.000671	0.0005321	0.21	outcome	TRUE	reported	FVRUio
261	rs4784541	16	51704452	T	C	0.484459	-0.000657	0.0004796	0.18	outcome	TRUE	reported	FVRUio
198	rs2744133	6	22392260	A	G	0.727242	-0.000648	0.000528	0.21	outcome	TRUE	reported	FVRUio
227	rs3453575	4	2246927	C	T	0.959133	-0.000647	0.001195	0.55	outcome	TRUE	reported	FVRUio
339	rs7142155	2	177053298	G	C	0.7176	-0.000641	0.0005222	0.2	outcome	TRUE	reported	FVRUio
258	rs4690774	4	144085327	A	T	0.521206	-0.000629	0.0004722	0.17	outcome	TRUE	reported	FVRUio
56	rs1167266	19	46180184	C	T	0.808402	-0.000619	0.0005959	0.27	outcome	TRUE	reported	FVRUio
254	rs4577304	2	73403040	T	C	0.514474	-0.000612	0.0004734	0.19	outcome	TRUE	reported	FVRUio
271	rs4955575	3	169534538	A	C	0.748361	-0.00061	0.0005422	0.28	outcome	TRUE	reported	FVRUio
324	rs6788907	3	158212823	G	A	0.735814	-0.000609	0.000532	0.25	outcome	TRUE	reported	FVRUio
262	rs483071	13	22294117	C	T	0.372863	-0.000608	0.0004933	0.21	outcome	TRUE	reported	FVRUio
416	rs927315	9	4117713	C	T	0.526914	-0.000595	0.0004746	0.2	outcome	TRUE	reported	FVRUio
133	rs1724582	13	73131694	A	C	0.631113	-0.000594	0.0004916	0.22	outcome	TRUE	reported	FVRUio
171	rs2289124	11	89224477	G	A	0.830942	-0.000594	0.0006343	0.32	outcome	TRUE	reported	FVRUio
70	rs1209384	1	43765089	A	G	0.380785	-0.000593	0.0004854	0.24	outcome	TRUE	reported	FVRUio
405	rs8030856	15	40314967	C	G	0.602168	-0.000576	0.0004844	0.23	outcome	TRUE	reported	FVRUio
57	rs1169078	12	122416254	C	G	0.682349	-0.000576	0.0005055	0.26	outcome	TRUE	reported	FVRUio
408	rs8125763	20	17883531	C	A	0.532127	-0.000565	0.0004732	0.24	outcome	TRUE	reported	FVRUio
100	rs1308120	3	185322643	G	A	0.649976	-0.000563	0.0004931	0.26	outcome	TRUE	reported	FVRUio
223	rs3413036	10	48411796	G	T	0.884167	-0.000561	0.0007366	0.46	outcome	TRUE	reported	FVRUio
109	rs1375564	3	85656311	C	T	0.371727	-0.000556	0.0004936	0.25	outcome	TRUE	reported	FVRUio
99	rs1301677	2	55779476	C	T	0.24727	-0.000556	0.0005476	0.3	outcome	TRUE	reported	FVRUio
11	rs1021453	6	161720674	A	G	0.684153	-0.000552	0.0005127	0.27	outcome	TRUE	reported	FVRUio
323	rs6761754	7	90297177	C	G	0.673205	-0.000543	0.0005026	0.26	outcome	TRUE	reported	FVRUio
281	rs573455	11	117267884	A	G	0.468546	-0.00053	0.0004703	0.25	outcome	TRUE	reported	FVRUio
52	rs1160435	11	45351729	C	A	0.834615	-0.000526	0.000636	0.41	outcome	TRUE	reported	FVRUio
24	rs1075044	11	130469044	C	T	0.328743	-0.000525	0.000501	0.31	outcome	TRUE	reported	FVRUio
381	rs7645234	9	35906471	C	T	0.800972	-0.000525	0.0006117	0.38	outcome	TRUE	reported	FVRUio
86	rs1264359	4	156639846	A	G	0.637865	-0.000518	0.0004877	0.26	outcome	TRUE	reported	FVRUio
77	rs1238606	17	60763400	G	C	0.584753	-0.000516	0.0004796	0.27	outcome	TRUE	reported	FVRUio
55	rs1165392	17	2012094	C	T	0.624911	-0.000513	0.0004896	0.31	outcome	TRUE	reported	FVRUio
325	rs6788984	3	41107173	A	G	0.852482	-0.000507	0.0006691	0.46	outcome	TRUE	reported	FVRUio
370	rs7493678	14	39400917	A	T	0.652749	-0.000494	0.000495	0.31	outcome	TRUE	reported	FVRUio
294	rs6090907	20	47410231	G	A	0.852358	-0.000493	0.0006636	0.43	outcome	TRUE	reported	FVRUio
127	rs1623474	10	18471794	C	T	0.663307	-0.000486	0.0004994	0.34	outcome	TRUE	reported	FVRUio
87	rs1265649	5	32831939	T	C	0.403652	-0.000483	0.0004784	0.33	outcome	TRUE	reported	FVRUio
306	rs6251291	8	82828857	A	G	0.587525	-0.000482	0.0004783	0.35	outcome	TRUE	reported	FVRUio
434	rs9897429	17	47518378	G	A	0.485309	-0.00048	0.0004798	0.34	outcome	TRUE	reported	FVRUio
297	rs6108787	20	10967214	T	G	0.522859	-0.000473	0.000471	0.3	outcome	TRUE	reported	FVRUio
69	rs1206337	1	59621911	G	A	0.61908	-0.000472	0.0004885	0.34	outcome	TRUE	reported	FVRUio
182	rs2470004	8	120358445	C	T	0.182928	-0.000466	0.0006075	0.46	outcome	TRUE	reported	FVRUio
404	rs7980644	12	79959658	A	G	0.164245	-0.000463	0.0006372	0.49	outcome	TRUE	reported	FVRUio
221	rs3407272	7	130432469	G	A	0.514552	-0.000462	0.0004716	0.32	outcome	TRUE	reported	FVRUio
153	rs1906672	8	38130025	G	A	0.767988	-0.000455	0.0005569	0.37	outcome	TRUE	reported	FVRUio
301	rs6207662	17	61090958	A	G	0.805147	-0.000445	0.0005941	0.47	outcome	TRUE	reported	FVRUio
154	rs1957563	5	157474590	C	T	0.733561	-0.000436	0.0005313	0.44	outcome	TRUE	reported	FVRUio
14	rs1042051	19	45298461	G	T	0.964174	-0.000429	0.001277	0.77	outcome	TRUE	reported	FVRUio
383	rs7703237	15	90010780	C	T	0.859885	-0.000423	0.0006796	0.53	outcome	TRUE	reported	FVRUio
423	rs9507885	13	27951090	C	T	0.910133	-0.000417	0.0008394	0.63	outcome	TRUE	reported	FVRUio
264	rs4873492	8	51947549	C	T	0.822331	-0.000417	0.0006194	0.49	outcome	TRUE	reported	FVRUio
181	rs246973	5	68007803	C	T	0.712901	-0.000411	0.000528	0.45	outcome	TRUE	reported	FVRUio
37	rs1115909	14	75074316	G	A	0.55513	-0.000411	0.0004775	0.39	outcome	TRUE	reported	FVRUio
29	rs1086403	1	207208335	C	T	0.599411	-0.000408	0.0004799	0.4	outcome	TRUE	reported	FVRUio
265	rs488834	1	10767902	C	T	0.241479	-0.000406	0.0005508	0.44	outcome	TRUE	reported	FVRUio
359	rs7310393	5	66280577	T	C	0.732255	-0.000405	0.0005366	0.49	outcome	TRUE	reported	FVRUio
66	rs1197752	7	46008110	G	A	0.597477	-0.000404	0.0004798	0.38	outcome	TRUE	reported	FVRUio
287	rs6026744	20	57742388	A	T	0.881488	-0.0004	0.0007306	0.63	outcome	TRUE	reported	FVRUio
17	rs1049212	16	4932929	A	G	0.442207	-0.00039	0.0004774	0.42	outcome	TRUE	reported	FVRUio
28	rs1080433	2	227185749	T	C	0.574475	-0.000388	0.00048	0.41	outcome	TRUE	reported	FVRUio
365	rs740746	10	115792787	G	A	0.269199	-0.000385	0.0005322	0.45	outcome	TRUE	reported	FVRUio
31	rs1091412	1	180865798	T	C	0.614208	-0.000376	0.0004835	0.45	outcome	TRUE	reported	FVRUio
241	rs3845811	2	208521512	C	G	0.57498	-0.00037	0.0004769	0.42	outcome	TRUE	reported	FVRUio
150	rs1889785	1	16348729	G	A	0.538269	-0.00037	0.000472	0.4	outcome	TRUE	reported	FVRUio
232	rs3568030	7	130973495	C	T	0.41393	-0.00037	0.0004833	0.43	outcome	TRUE	reported	FVRUio
302	rs6208223	18	22676071	T	A	0.725075	-0.000369	0.000534	0.5	outcome	TRUE	reported	FVRUio
296	rs6099198	4	54801228	T	G	0.881773	-0.000357	0.0007466	0.63	outcome	TRUE	reported	FVRUio
151	rs1892675	3	20073193	T	A	0.987947	-0.000353	0.0021708	0.85	outcome	TRUE	reported	FVRUio
4	rs1004876	2	174977976	T	G	0.522059	-0.000353	0.0004738	0.44	outcome	TRUE	reported	FVRUio
293	rs6078093	20	11168669	G	A	0.576711	-0.000347	0.0004794	0.49	outcome	TRUE	reported	FVRUio
216	rs2951987	8	26000422	G	T	0.906472	-0.000343	0.0008131	0.71	outcome	TRUE	reported	FVRUio
80	rs1251198	4	46595623	T	G	0.819804	-0.000342	0.000616	0.58	outcome	TRUE	reported	FVRUio
333	rs6997996	8	25891653	A	G	0.742348	-0.000337	0.0005391	0.52	outcome	TRUE	reported	FVRUio
48	rs1152620	2	43196694	A	T	0.914885	-0.000332	0.0008484	0.67	outcome	TRUE	reported	FVRUio
196	rs2689690	10	95899706	C	T	0.638691	-0.000329	0.0004932	0.48	outcome	TRUE	reported	FVRUio
401	rs7953936	3	154680449	T	C	0.89873	-0.00032	0.0007778	0.66	outcome	TRUE	reported	FVRUio
12	rs1022421	7	151413194	T	C	0.725327	-0.000316	0.000529	0.56	outcome	TRUE	reported	FVRUio
106	rs1335865	5	157938070	A	G	0.869271	-0.000308	0.0006964	0.63	outcome	TRUE	reported	FVRUio
237	rs3802517	10	28233469	T	A	0.545579	-0.000301	0.0004717	0.53	outcome	TRUE	reported	FVRUio
308	rs6438857	3	124557643	T	C	0.57805	-0.000297	0.0004772	0.51	outcome	TRUE	reported	FVRUio
15	rs1044822	2	230629138	C	T	0.849145	-0.000293	0.0006586	0.63	outcome	TRUE	reported	FVRUio
84	rs1262765	21	44760603	G	A	0.723132	-0.000287	0.0005257	0.63	outcome	TRUE	reported	FVRUio
175	rs2327429	6	134209837	T	C	0.703727	-0.000287	0.0005177	0.59	outcome	TRUE	reported	FVRUio
273	rs4961293	8	141812374	C	T	0.540441	-0.000286	0.0004743	0.54	outcome	TRUE	reported	FVRUio
45	rs1130864	17	7171356	C	T	0.457581	-0.000282	0.0004748	0.58	outcome	TRUE	reported	FVRUio
299	rs6191761	12	48144520	G	C	0.621432	-0.00028	0.0004862	0.56	outcome	TRUE	reported	FVRUio
420	rs9361836	6	82235408	C	T	0.690632	-0.000275	0.000509	0.54	outcome	TRUE	reported	FVRUio
190	rs2610990	4	18008232	A	G	0.259232	-0.000275	0.000536	0.59	outcome	TRUE	reported	FVRUio
167	rs2238787	22	19976406	G	A	0.716137	-0.000271	0.0005208	0.63	outcome	TRUE	reported	FVRUio
76	rs12321	22	29453193	G	C	0.562214	-0.000267	0.000474	0.59	outcome	TRUE	reported	FVRUio
42	rs1124131	5	114428167	C	T	0.689106	-0.000257	0.0005083	0.62	outcome	TRUE	reported	FVRUio
384	rs7703560	5	67678506	A	G	0.690194	-0.000248	0.0005153	0.63	outcome	TRUE	reported	FVRUio
252	rs4511593	17	7455536	C	T	0.351683	-0.000246	0.0004939	0.62	outcome	TRUE	reported	FVRUio
335	rs702395	5	140086677	C	T	0.564365	-0.000241	0.0004737	0.64	outcome	TRUE	reported	FVRUio
40	rs1121002	1	41865293	A	G	0.638119	-0.000228	0.0004903	0.63	outcome	TRUE	reported	FVRUio
3	rs1004840	18	54578482	C	T	0.638009	-0.000228	0.0004896	0.67	outcome	TRUE	reported	FVRUio
343	rs7213273	17	43155914	G	A	0.343629	-0.000223	0.0004965	0.61	outcome	TRUE	reported	FVRUio
169	rs2276153	11	58407740	C	G	0.761734	-0.000222	0.0005525	0.7	outcome	TRUE	reported	FVRUio
295	rs6090907	4	83830244	G	C	0.735294	-0.000222	0.000534	0.72	outcome	TRUE	reported	FVRUio
159	rs2065498	13	41893105	T	G	0.174312	-0.000214	0.0006275	0.69	outcome	TRUE	reported	FVRUio
217	rs2957688	11	10364963	G	A	0.531521	-0.000211	0.000472	0.67	outcome	TRUE	reported	FVRUio
245	rs404100	1	25366987	C	T	0.556986	-0.000211	0.0004735	0.65	outcome	TRUE	reported	FVRUio
75	rs1226418	10	32289986	C	T	0.815399	-0.000205	0.0006046	0.73	outcome	TRUE	reported	FVRUio
341	rs7186298	16	21088031	C	T	0.56843	-0.000205	0.0004731	0.66	outcome	TRUE	reported	FVRUio
316	rs658780	1	78555928	T	G	0.746763	-0.000202	0.0005431	0.67	outcome	TRUE	reported	FVRUio
185	rs2498323	4	3451109	G	A	0.901659	-0.000198	0.0007884	0.78	outcome	TRUE	reported	FVRUio
250	rs4440615	8	141057641	G	A	0.374402	-0.000197	0.0004857	0.67	outcome	TRUE	reported	FVRUio
309	rs6445583	3	53562894	G	A	0.254131	-0.000196	0.0005399	0.68	outcome	TRUE	reported	FVRUio
149	rs1882961	21	16556367	C	T	0.69885	-0.000187	0.0005157	0.7	outcome	TRUE	reported	FVRUio
78	rs1246460	2	43397614	G	A	0.376439	-0.000186	0.0004913	0.76	outcome	TRUE	reported	FVRUio
213	rs2904315	11	48109948	A	G	0.323205	-0.000183	0.0005084	0.72	outcome	TRUE	reported	FVRUio
144	rs1814951	4	111408718	G	A	0.118241	-0.000171	0.0007315	0.83	outcome	TRUE	reported	FVRUio
138	rs1761048	2	177540601	T	A	0.432802	-0.000169	0.0004782	0.73	outcome	TRUE	reported	FVRUio
332	rs6978112	7	1966841	C	T	0.582366	-0.000161	0.0004801	0.74	outcome	TRUE	reported	FVRUio
183	rs2493134	1	230849359	T	C	0.581316	-0.000155	0.0004816	0.75	outcome	TRUE	reported	FVRUio
410	rs8180684	6	143200936	C	T	0.710665	-0.000152	0.0005202	0.74	outcome	TRUE	reported	FVRUio
272	rs4957026	5	361148	A	G	0.337669	-0.00015	0.0004974	0.79	outcome	TRUE	reported	FVRUio
432	rs9880098	3	133949366	G	A	0.622195	-0.000143	0.0004864	0.78	outcome	TRUE	reported	FVRUio
406	rs8044992	16	24811207	T	C	0.715234	-0.000143	0.0005203	0.8	outcome	TRUE	reported	FVRUio
372	rs7514579	1	94051350	A	C	0.765539	-0.000142	0.0005609	0.77	outcome	TRUE	reported	FVRUio
422	rs9486916	6	109013930	C	T	0.79197	-0.000134	0.0006038	0.86	outcome	TRUE	reported	FVRUio
158	rs2060664	16	60652439	T	C	0.751008	-0.000131	0.0005425	0.83	outcome	TRUE	reported	FVRUio
90	rs1266843	7	47548893	T	C	0.752164	-0.000129	0.0005443	0.81	outcome	TRUE	reported	FVRUio
251	rs4499560	3	70920485	A	T	0.32263	-0.000121	0.0005093	0.8	outcome	TRUE	reported	FVRUio
178	rs236916	11	117089628	G	A	0.858803	-0.000119	0.0006824	0.81	outcome	TRUE	reported	FVRUio
222	rs3407986	1	27407850	C	T	0.743035	-0.000118	0.0005423	0.79	outcome	TRUE	reported	FVRUio
424	rs9526707	13	51489186	G	A	0.682968	-0.00011	0.0005068	0.8	outcome	TRUE	reported	FVRUio
96	rs1290784	3	169096900	C	T	0.55331	-0.000105	0.0004733	0.84	outcome	TRUE	reported	FVRUio
259	rs4691666	4	156398688	C	T	0.471824	-0.000101	0.0004721	0.89	outcome	TRUE	reported	FVRUio
396	rs7899848	12	434755	C	G	0.744233	-0.000101	0.0005388	0.86	outcome	TRUE	reported	FVRUio
202	rs2815063	6	39262535	C	A	0.864106	-8.72E-05	0.0006984	0.87	outcome	TRUE	reported	FVRUio
425	rs9549627	13	113652369	G	A	0.884226	-8.14E-05	0.0007407	0.98	outcome	TRUE	reported	FVRUio
366	rs743395	3	37598382	C	T	0.626369	-7.34E-05	0.0004948	0.86	outcome	TRUE	reported	FVRUio
32	rs1094104	5	33194751	T	G	0.712595	-6.63E-05	0.0005192	0.88	outcome	TRUE	reported	FVRUio
257	rs4651224	1	184585182	C	T	0.547085	-6.39E-05	0.0004785	0.89	outcome	TRUE	reported	FVRUio
409	rs8142376	22	32001037	C	T	0.511568	-6.36E-05	0.0004701	0.93	outcome	TRUE	reported	FVRUio
340	rs7154723	14	98590629	G	A	0.622894	-6.30E-05	0.0004852	0.91	outcome	TRUE	reported	FVRUio
288	rs6029756	20	40266681	G	A	0.676721	-6.13E-05	0.0005093	0.92	outcome	TRUE	reported	FVRUio
179	rs2384063	2	25187115	C	T	0.239096	-5.32E-05	0.0005502	0.93	outcome	TRUE	reported	FVRUio
327	rs6870654	5	63831964	T	C	0.738898	-4.85E-05	0.0005355	0.87	outcome	TRUE	reported	FVRUio
391	rs7830689	8	92777060	G	A	0.572709	-4.67E-05	0.0004782	0.94	outcome	TRUE	reported	FVRUio
354	rs7304077	19	30351998	C	T	0.934855	-4.61E-05	0.0009581	0.96	outcome	TRUE	reported	FVRUio
389	rs778124	1	56606206	G	A	0.62461	-4.49E-05	0.0004856	0.91	outcome	TRUE	reported	FVRUio
342	rs7211535	17	19922364	A	G	0.478904	-4.29E-05	0.0004714	0.86	outcome	TRUE	reported	FVRUio
62	rs1187424	18	42596789	C	T	0.706494	-3.81E-05	0.0005159	0.97	outcome	TRUE	reported	FVRUio
428	rs977184	7	28650761	T	C	0.638869	-3.63E-05	0.0004946	0.98	outcome	TRUE	reported	FVRUio
331	rs6961048	7	27328187	C	G	0.899337	-3.08E-05	0.0007811	0.99	outcome	TRUE	reported	FVRUio
219	rs3104552	9	128153616	T	C	0.55416	-3.05E-05	0.0004764	0.9	outcome	TRUE	reported	FVRUio
200	rs2776037	21	16317933	T	C	0.417392	-2.79E-05	0.0004787	0.97	outcome	TRUE	reported	FVRUio
112	rs1408945	1	42364877	G	T	0.581156	-2.62E-05	0.0004786	0.97	outcome	TRUE	reported	FVRUio
314	rs6558535	8	1710632	T	C	0.283012	-1.99E-05	0.000523	0.97	outcome	TRUE	reported	FVRUio
46	rs1132646	22	30135079	C	T	0.954442	-1.85E-05	0.0011256	0.99	outcome	TRUE	reported	FVRUio
255	rs4598218	8	129483956	C	T	0.397668	-1.46E-05	0.0004868	0.93	outcome	TRUE	reported	FVRUio
321	rs6732123	2	69534650	G	C	0.577305	-1.4E-05	0.0004783	0.96	outcome	TRUE	reported	FVRUio
94	rs1275985	2	26911745	C	T	0.393111	-1.14E-05	0.0004851	1	outcome	TRUE	reported	FVRUio
98	rs1292655	16	81510155	G	A	0.688141	-8.66E-06	0.0005096	0.99	outcome	TRUE	reported	FVRUio
274	rs4980379	11	1888614	C	T	0.640392	-6.59E-08	0.0004924	0.98	outcome	TRUE	reported	FVRUio
206	rs2857871	22	50727921	T	C	0.608161	1.50788E-	0.0004819	0.93	outcome	TRUE	reported	FVRUio
110	rs1382472	11	27273967	G	A	0.593052	2.129E-05	0.0004802	0.99	outcome	TRUE	reported	FVRUio
433	rs9886665	9	22942770	T	C	0.276372	2.46289E-	0.0005347	0.91	outcome	TRUE	reported	FVRUio
307	rs629864	11	100699139	C	T	0.346655	3.125E-05	0.000498	0.92	outcome	TRUE	reported	FVRUio
8	rs1012089	16	74171973	C	G	0.48485	3.91128E-	0.0004736	0.87	outcome	TRUE	reported	FVRUio
134	rs1724975	12	90060586	G	A	0.824506	4.04054E-	0.0006192	0.96	outcome	TRUE	reported	FVRUio
192	rs262986	3	183435713	G	A	0.525042	4.26337E-	0.0004734	0.94	outcome	TRUE	reported	FVRUio
313	rs6540119	16	87984477	A	T	0.329418	4.33151E-	0.0005055	0.92	outcome	TRUE	reported	FVRUio
344	rs7221807	17	18221799	T	C	0.568216	4.46155E-	0.0004752	0.95	outcome	TRUE	reported	FVRUio
9	rs1018800	2	66773469	C	T	0.614465	4.95999E-	0.0004834	0.93	outcome	TRUE	reported	FVRUio
328	rs6892983	5	127845030	C	A	0.582479	5.32538E-	0.0004816	0.91	outcome	TRUE	reported	FVRUio
187	rs2589218	15	96785017	T	C	0.729487	5.56505E-	0.0005308	0.91	outcome	TRUE	reported	FVRUio
140	rs1776025	2	19744462	T	C	0.581208	6.86197E-	0.000477	0.88	outcome	TRUE	reported	FVRUio
407	rs8105174	19	10347032	C	T	0.81562	6.91264E-	0.0006059	0.89	outcome	TRUE	reported	FVRUio
21	rs1057040	17	76799795	G	A	0.483215	6.98843E-	0.0004705	0.84	outcome	TRUE	reported	FVRUio
139	rs177551	11	16930289	C	A	0.865509	7.06737E-	0.0006885	0.9	outcome	TRUE	reported	FVRUio
399	rs7926110	11	107086143	T	G	0.667503	7.42468E-	0.0005017	0.85	outcome	TRUE	reported	FVRUio
276	rs5573219	2	162278233	G	T	0.908115	7.45445E-	0.0008173	0.91	outcome	TRUE	reported	FVRUio
19	rs1050141	11	72088806	G	A	0.928503	7.63079E-	0.0009256	0.91	outcome	TRUE	reported	FVRUio
362	rs7372760	7	149474622	G	A	0.933392	8.62811E-	0.0009421	0.94	outcome	TRUE	reported	FVRUio
278	rs5640782	18	42179819	C	T	0.739595	8.99031E-	0.0005382	0.83	outcome	TRUE	reported	FVRUio
116	rs1436138	17	75316880	A	G	0.634728	9.24655E-	0.0004902	0.89	outcome	TRUE	reported	FVRUio
415	rs908951	16	89697625	C	T	0.573	9.27059E-	0.0004789	0.87	outcome	TRUE	reported	FVRUio
260	rs4775769	15	48939888	T	G	0.0916519	9.34754E-	0.000817	0.92	outcome	TRUE	reported	FVRUio
338	rs7107356	11	47676170	A	G	0.494947	9.67192E-	0.0004691	0.85	outcome	TRUE	reported	FVRUio
124	rs1551355	17	30032420	C	T	0.77487	9.69114E-	0.0005635	0.82	outcome	TRUE	reported	FVRUio
355	rs7304679	19	49605705	G	A	0.842821	0.0001003	0.000646	0.92	outcome	TRUE	reported	FVRUio
210	rs2868879	7	19039605	T	C	0.802735	0.000109	0.0005947	0.84	outcome	TRUE	reported	FVRUio
320	rs6731373	2	68503044	G	A	0.646512	0.0001161	0.0004915	0.78	outcome	TRUE	reported	FVRUio
22	rs1074696	9	77238558	A	G	0.189233	0.0001179	0.0006033	0.8	outcome	TRUE	reported	FVRUio
146	rs1848994	19	22111366	G	A	0.717214	0.0001188	0.0005219	0.82	outcome	TRUE	reported	FVRUio
168	rs2249105	2	65287896	A	G	0.631967	0.0001202	0.000486	0.78	outcome	TRUE	reported	FVRUio
412	rs869396	4	169688000	C	A	0.529528	0.0001277	0.0004748	0.8	outcome	TRUE	reported	FVRUio
377	rs7596140	13	56398286	G	A	0.846381	0.0001334	0.0006525	0.82	outcome	TRUE	reported	FVRUio
388	rs7765526	6	147713764	A	G	0.462221	0.0001399	0.0004766	0.81	outcome	TRUE	reported	FVRUio
204	rs2842925	15	66931617	G	A	0.66811	0.0001476	0.0005066	0.75	outcome	TRUE	reported	FVRUio
54	rs1164137	16	1347717	C	A	0.409134	0.0001498	0.0004819	0.74	outcome	TRUE	reported	FVRUio
121	rs1493132	4	108861082	T	C	0.655236	0.000152	0.0004948	0.77	outcome	TRUE	reported	FVRUio
253	rs4553000	9	34223553	C	T	0.487896	0.0001534	0.0004699	0.75	outcome	TRUE	reported	FVRUio
18	rs1050112	11	30192151	T	C	0.639856	0.0001578	0.0004896	0.76	outcome	TRUE	reported	FVRUio
7	rs1010064	12	20000315	A	C	0.811403	0.0001609	0.000602	0.8	outcome	TRUE	reported	FVRUio
280	rs571689	19	49207554	C	T	0.472378	0.0001637	0.0004738	0.71	outcome	TRUE	reported	FVRUio
64	rs1196021	5	157817634	T	C	0.624788	0.0001649	0.0004854	0.79	outcome	TRUE	reported	FVRUio
218	rs3098186	15	50810621	C	T	0.481773	0.0001672	0.000471	0.75	outcome	TRUE	reported	FVRUio
345	rs7245140	18	43095231	T	C	0.811897	0.0001678	0.0006071	0.79	outcome	TRUE	reported	FVRUio
305	rs6248185	7	106412082	G	A	0.802751	0.0001745	0.0005903	0.73	outcome	TRUE	reported	FVRUio
50	rs1158516	1	150572037	T	A	0.433082	0.0001883	0.0004785	0.68	outcome	TRUE	reported	FVRUio
379	rs7618284	3	66422246	G	C	0.651076	0.0001895	0.0005059	0.73	outcome	TRUE	reported	FVRUio
413	rs871004	11	28512458	G	A	0.65046	0.0001898	0.0004948	0.72	outcome	TRUE	reported	FVRUio
395	rs786923	1	89242954	C	T	0.372888	0.0001907	0.0004884	0.75	outcome	TRUE	reported	FVRUio
240	rs3828282	2	218779144	C	G	0.441672	0.0002092	0.0004777	0.68	outcome	TRUE	reported	FVRUio
371	rs7501697	14	100197940	C	T	0.859119	0.0002103	0.0006789	0.76	outcome	TRUE	reported	FVRUio
5	rs1006545	10	102553647	G	T	0.109493	0.0002178	0.0007534	0.82	outcome	TRUE	reported	FVRUio
356	rs7304992	7	4669949	A	G	0.80798	0.0002372	0.0006006	0.72	outcome	TRUE	reported	FVRUio
16	rs1046010	18	73034151	A	G	0.480684	0.0002404	0.0004731	0.61	outcome	TRUE	reported	FVRUio
303	rs6217047	2	146989797	T	C	0.603697	0.0002405	0.0004898	0.64	outcome	TRUE	reported	FVRUio
74	rs1225537	10	114808902	G	T	0.714034	0.0002517	0.0005191	0.62	outcome	TRUE	reported	FVRUio
205	rs2857235	19	31867447	A	C	0.600194	0.0002594	0.0004817	0.62	outcome	TRUE	reported	FVRUio
26	rs1077721	12	90349999	G	A	0.47057	0.0002605	0.0004709	0.6	outcome	TRUE	reported	FVRUio
242	rs3860770	5	173301427	G	A	0.722152	0.000267	0.0005263	0.61	outcome	TRUE	reported	FVRUio
429	rs9848170	3	11495983	G	C	0.405959	0.0002671	0.0004795	0.58	outcome	TRUE	reported	FVRUio
322	rs6737318	2	114083120	A	G	0.780908	0.0002688	0.00057	0.7	outcome	TRUE	reported	FVRUio
286	rs6026578	20	57463472	C	G	0.375188	0.0002691	0.000488	0.59	outcome	TRUE	reported	FVRUio
263	rs4834792	4	120555696	T	A	0.519145	0.0002765	0.0004714	0.51	outcome	TRUE	reported	FVRUio
235	rs3764400	17	46123932	T	C	0.871594	0.0002766	0.0007034	0.65	outcome	TRUE	reported	FVRUio
326	rs6811555	3	27704702	A	G	0.982055	0.000286	0.0017788	0.87	outcome	TRUE	reported	FVRUio
101	rs1310726	4	63768826	G	A	0.627858	0.0002883	0.0004871	0.61	outcome	TRUE	reported	FVRUio
435	rs9899540	17	30777924	A	T	0.394685	0.0002946	0.0004865	0.54	outcome	TRUE	reported	FVRUio
118	rs1465507	16	66781040	T	C	0.960829	0.0003004	0.0012138	0.82	outcome	TRUE	reported	FVRUio
59	rs1172853	17	7870642	T	C	0.926397	0.0003034	0.0009027	0.74	outcome	TRUE	reported	FVRUio
304	rs6230974	4	48713862	G	A	0.539591	0.0003047	0.0004719	0.49	outcome	TRUE	reported	FVRUio
411	rs848445	7	77572461	T	C	0.282401	0.0003203	0.0005223	0.52	outcome	TRUE	reported	FVRUio
156	rs2014408	11	16365282	C	T	0.789893	0.0003246	0.000579	0.57	outcome	TRUE	reported	FVRUio
186	rs2580350	2	121996007	G	A	0.4444	0.0003342	0.0004766	0.45	outcome	TRUE	reported	FVRUio
137	rs1756239	14	100133250	C	T	0.587279	0.0003351	0.0004776	0.52	outcome	TRUE	reported	FVRUio
267	rs4894132	2	180738654	T	C	0.733488	0.0003399	0.0005319	0.57	outcome	TRUE	reported	FVRUio
2	rs1004530	5	127411454	C	G	0.764705	0.0003575	0.0005601	0.54	outcome	TRUE	reported	FVRUio
92	rs1269427	2	213188795	T	C	0.305937	0.0003645	0.000523	0.45	outcome	TRUE	reported	FVRUio
89	rs1266103	6	163737476	T	C	0.77201	0.0003653	0.0005719	0.51	outcome	TRUE	reported	FVRUio
376	rs7567296	7	131321010	C	T	0.957696	0.0003673	0.0011784	0.75	outcome	TRUE	reported	FVRUio
173	rs2291516	19	11508177	G	A	0.896944	0.0003684	0.0007749	0.64	outcome	TRUE	reported	FVRUio
346	rs7255933	19	45766729	G	A	0.747374	0.0003684	0.0005422	0.46	outcome	TRUE	reported	FVRUio
348	rs7274250	1	221265336	C	T	0.701294	0.0003691	0.000515	0.46	outcome	TRUE	reported	FVRUio
236	rs3772219	3	56771251	A	C	0.67124	0.0003791	0.000501	0.44	outcome	TRUE	reported	FVRUio
189	rs2607015	6	31762843	G	C	0.58506	0.0003931	0.0004776	0.4	outcome	TRUE	reported	FVRUio
419	rs9356744	6	20685486	T	C	0.684812	0.0003937	0.0005077	0.41	outcome	TRUE	reported	FVRUio
285	rs6019165	9	753648	A	G	0.813532	0.0003967	0.0006044	0.5	outcome	TRUE	reported	FVRUio
244	rs3980686	3	168697602	G	T	0.886256	0.0004024	0.0007468	0.63	outcome	TRUE	reported	FVRUio
117	rs1437649	18	48132646	G	A	0.765908	0.0004069	0.0005593	0.47	outcome	TRUE	reported	FVRUio
282	rs5742643	12	102837863	T	C	0.247682	0.0004141	0.000541	0.45	outcome	TRUE	reported	FVRUio
6	rs1006969	5	1279790	C	T	0.735504	0.0004148	0.0005348	0.43	outcome	TRUE	reported	FVRUio
212	rs2900568	9	116689758	C	T	0.485825	0.0004175	0.0004714	0.38	outcome	TRUE	reported	FVRUio
224	rs3441314	18	777282	T	A	0.810696	0.0004182	0.0006038	0.47	outcome	TRUE	reported	FVRUio
367	rs7439567	4	138464842	C	T	0.58602	0.0004324	0.0004797	0.38	outcome	TRUE	reported	FVRUio
351	rs7284788	2	86326717	A	G	0.663878	0.0004523	0.0004983	0.36	outcome	TRUE	reported	FVRUio
163	rs2161967	2	218680529	T	G	0.435166	0.0004527	0.0004786	0.38	outcome	TRUE	reported	FVRUio
35	rs1111788	1	217736417	A	G	0.769789	0.0004543	0.0005593	0.41	outcome	TRUE	reported	FVRUio
298	rs6177259	1	56979681	A	G	0.879433	0.0004646	0.0007248	0.51	outcome	TRUE	reported	FVRUio
361	rs7320104	13	115059765	A	G	0.843518	0.0004662	0.0006531	0.46	outcome	TRUE	reported	FVRUio
385	rs7722243	5	50818437	G	A	0.491719	0.000467	0.0004726	0.32	outcome	TRUE	reported	FVRUio
193	rs263532	1	2164116	T	C	0.573043	0.0004673	0.000477	0.32	outcome	TRUE	reported	FVRUio
107	rs1341275	2	191634958	G	A	0.727771	0.0004818	0.0005286	0.37	outcome	TRUE	reported	FVRUio
230	rs3528896	3	153732022	G	A	0.738635	0.0004892	0.0005348	0.37	outcome	TRUE	reported	FVRUio
234	rs3754944	2	231279616	C	A	0.42044	0.0004918	0.0004782	0.3	outcome	TRUE	reported	FVRUio
430	rs9869437	3	196228360	C	A	0.643669	0.0005093	0.0004947	0.29	outcome	TRUE	reported	FVRUio
126	rs1575290	6	7715689	C	T	0.527568	0.0005154	0.0004715	0.27	outcome	TRUE	reported	FVRUio
184	rs2493296	1	3327032	C	T	0.862986	0.0005219	0.000685	0.44	outcome	TRUE	reported	FVRUio
239	rs3819532	12	2436837	T	C	0.395838	0.0005239	0.000481	0.28	outcome	TRUE	reported	FVRUio
203	rs2833834	21	33814378	C	A	0.718565	0.0005314	0.0005254	0.31	outcome	TRUE	reported	FVRUio
95	rs1288587	14	104007555	A	G	0.231426	0.0005325	0.0005733	0.36	outcome	TRUE	reported	FVRUio
283	rs5794634	10	63499951	T	C	0.853804	0.0005366	0.0006662	0.4	outcome	TRUE	reported	FVRUio
160	rs2111557	3	169325621	C	T	0.53621	0.0005436	0.0004723	0.24	outcome	TRUE	reported	FVRUio
148	rs1871190	5	97953719	G	T	0.679512	0.0005438	0.0005084	0.28	outcome	TRUE	reported	FVRUio
318	rs6686433	11	65390803	G	A	0.778906	0.0005555	0.0005665	0.36	outcome	TRUE	reported	FVRUio
136	rs1745417	1	228204279	C	T	0.488672	0.0005557	0.0004706	0.24	outcome	TRUE	reported	FVRUio
214	rs2913920	5	141726983	C	T	0.228986	0.0005566	0.0005612	0.32	outcome	TRUE	reported	FVRUio
292	rs6058088	20	30139886	T	G	0.84065	0.0005639	0.0006672	0.39	outcome	TRUE	reported	FVRUio
20	rs1052501	3	41925398	C	T	0.17186	0.0005656	0.0006346	0.37	outcome	TRUE	reported	FVRUio
58	rs1172066	12	133086888	C	T	0.89377	0.0005708	0.0007673	0.46	outcome	TRUE	reported	FVRUio
30	rs1086682	8	23401534	C	T	0.733101	0.0005721	0.0005377	0.3	outcome	TRUE	reported	FVRUio
129	rs1664781	5	53276301	G	A	0.302619	0.0005796	0.0005114	0.23	outcome	TRUE	reported	FVRUio
279	rs5714081	14	68018247	C	G	0.828186	0.000597	0.000622	0.35	outcome	TRUE	reported	FVRUio
103	rs1314920	4	89750668	T	C	0.779156	0.0005972	0.0005695	0.26	outcome	TRUE	reported	FVRUio
123	rs1544861	11	10679441	T	C	0.324798	0.0006084	0.000504	0.21	outcome	TRUE	reported	FVRUio
201	rs2801008	20	51788718	T	G	0.677404	0.0006169	0.0005065	0.22	outcome	TRUE	reported	FVRUio
51	rs1159210	10	122968964	G	A	0.693279	0.0006214	0.0005109	0.22	outcome	TRUE	reported	FVRUio
378	rs7615099	3	53143901	A	G	0.67146	0.000627	0.0005024	0.22	outcome	TRUE	reported	FVRUio
91	rs1269398	2	204085635	C	T	0.604436	0.0006325	0.0004806	0.18	outcome	TRUE	reported	FVRUio
246	rs4143175	12	67782397	T	C	0.241043	0.0006442	0.000552	0.24	outcome	TRUE	reported	FVRUio
135	rs1725708	2	135630498	A	G	0.820846	0.0006458	0.0006168	0.3	outcome	TRUE	reported	FVRUio
291	rs6054139	20	6327810	G	A	0.397826	0.0006476	0.0004805	0.17	outcome	TRUE	reported	FVRUio
402	rs7963801	12	79685226	T	C	0.41767	0.0006493	0.0004825	0.19	outcome	TRUE	reported	FVRUio
10	rs1020772	2	112744260	C	T	0.70754	0.0006506	0.0005159	0.21	outcome	TRUE	reported	FVRUio
270	rs4952609	2	40555733	A	G	0.745277	0.0006747	0.0005441	0.21	outcome	TRUE	reported	FVRUio
27	rs1078223	6	126228512	G	A	0.521833	0.0006825	0.0004698	0.14	outcome	TRUE	reported	FVRUio
93	rs1273164	1	169090660	C	T	0.601369	0.0006884	0.0004833	0.16	outcome	TRUE	reported	FVRUio
114	rs1422279	5	122470209	C	T	0.613392	0.0007	0.0004846	0.14	outcome	TRUE	reported	FVRUio
41	rs1122208	11	130273230	A	T	0.637038	0.0007079	0.0004889	0.16	outcome	TRUE	reported	FVRUio
266	rs4888408	16	75432824	G	A	0.410141	0.0007136	0.0004786	0.13	outcome	TRUE	reported	FVRUio
49	rs1154214	18	24546824	T	G	0.396597	0.0007166	0.0004812	0.14	outcome	TRUE	reported	FVRUio
209	rs2866780	4	26785356	A	T	0.594081	0.0007188	0.0004819	0.14	outcome	TRUE	reported	FVRUio
215	rs294886	6	159680744	T	C	0.281629	0.0007209	0.0005246	0.15	outcome	TRUE	reported	FVRUio
67	rs1199330	3	138101529	A	G	0.878846	0.0007386	0.0007249	0.34	outcome	TRUE	reported	FVRUio
155	rs1984195	6	79657391	G	A	0.510145	0.0007485	0.0004708	0.11	outcome	TRUE	reported	FVRUio
197	rs2724377	1	207974818	A	G	0.533345	0.0007658	0.000472	0.095	outcome	TRUE	reported	FVRUio
177	rs2354862	8	64501744	A	C	0.648642	0.0007748	0.0004966	0.11	outcome	TRUE	reported	FVRUio
1	rs1000423	17	59475642	C	T	0.278995	0.0007778	0.000529	0.14	outcome	TRUE	reported	FVRUio
104	rs1325335	8	68920135	C	T	0.688999	0.0007795	0.0005129	0.12	outcome	TRUE	reported	FVRUio
229	rs3494109	16	50550137	G	A	0.851703	0.0007809	0.0006612	0.24	outcome	TRUE	reported	FVRUio
25	rs1077675	1	113044328	G	T	0.926031	0.0007872	0.0009001	0.36	outcome	TRUE	reported	FVRUio
195	rs268263	2	164954174	T	A	0.257667	0.0008161	0.0005463	0.14	outcome	TRUE	reported	FVRUio
329	rs6921291	6	97066242	C	T	0.813242	0.0008266	0.0006033	0.19	outcome	TRUE	reported	FVRUio
23	rs1074957	10	82136664	G	T	0.455856	0.0008312	0.0004734	0.085	outcome	TRUE	reported	FVRUio
208	rs2865079	5	55861464	C	T	0.81005	0.000837	0.0005979	0.16	outcome	TRUE	reported	FVRUio
247	rs42032	7	92237426	G	A	0.739787	0.000839	0.0005378	0.11	outcome	TRUE	reported	FVRUio
390	rs7796	1	1684169	C	G	0.514719	0.0008475	0.0004734	0.074	outcome	TRUE	reported	FVRUio
373	rs7546155	1	15810172	C	T	0.800626	0.0008489	0.0005883	0.17	outcome	TRUE	reported	FVRUio
71	rs1213692	1	67007389	G	A	0.493887	0.0008611	0.000473	0.065	outcome	TRUE	reported	FVRUio
115	rs1433121	19	32591878	C	T	0.298285	0.0008628	0.0005173	0.093	outcome	TRUE	reported	FVRUio
207	rs2863403	4	157004869	T	A	0.666829	0.0008769	0.0005042	0.079	outcome	TRUE	reported	FVRUio
13	rs1028212	7	2529623	C	T	0.330395	0.0008771	0.0005002	0.074	outcome	TRUE	reported	FVRUio
85	rs1263757	3	121682388	A	G	0.470105	0.0008857	0.0004719	0.059	outcome	TRUE	reported	FVRUio
83	rs1261065	19	5006598	A	G	0.658243	0.0008991	0.0004968	0.066	outcome	TRUE	reported	FVRUio
353	rs7294548	12	50543201	A	G	0.363469	0.000928	0.0004897	0.055	outcome	TRUE	reported	FVRUio
34	rs1109790	4	106911321	T	C	0.146694	0.0009359	0.0006641	0.16	outcome	TRUE	reported	FVRUio
199	rs2745599	6	1613686	A	G	0.540183	0.000939	0.0004771	0.054	outcome	TRUE	reported	FVRUio
248	rs4245599	10	60365755	A	G	0.458314	0.0009488	0.000475	0.05	outcome	TRUE	reported	FVRUio
249	rs4284362	10	45377839	C	A	0.281996	0.0009596	0.0005315	0.077	outcome	TRUE	reported	FVRUio
269	rs4932373	15	91429287	A	C	0.68104	0.0009761	0.0005048	0.056	outcome	TRUE	reported	FVRUio
363	rs7395791	11	69262916	G	A	0.556021	0.0009786	0.0004739	0.037	outcome	TRUE	reported	FVRUio
176	rs2353940	4	145740898	T	C	0.755468	0.0009835	0.0005516	0.071	outcome	TRUE	reported	FVRUio
191	rs2627313	15	81006712	C	T	0.569389	0.0010123	0.0004788	0.037	outcome	TRUE	reported	FVRUio
228	rs3491784	8	95278307	G	C	0.872427	0.0010653	0.000704	0.14	outcome	TRUE	reported	FVRUio
47	rs1133400	10	134459388	A	G	0.781647	0.0010864	0.0005683	0.057	outcome	TRUE	reported	FVRUio
374	rs7555285	1	209970355	G	C	0.194025	0.0011052	0.000593	0.052	outcome	TRUE	reported	FVRUio
268	rs4926499	1	249155909	G	C	0.169938	0.001108	0.0006414	0.073	outcome	TRUE	reported	FVRUio
141	rs17762	11	22492454	G	A	0.914614	0.0011123	0.0008834	0.2	outcome	TRUE	reported	FVRUio
427	rs9651825	12	27159784	A	G	0.73018	0.0011127	0.0005314	0.037	outcome	TRUE	reported	FVRUio
337	rs7093894	10	124234880	C	A	0.844802	0.0011133	0.0006588	0.1	outcome	TRUE	reported	FVRUio
125	rs1565440	1	243387788	G	A	0.619181	0.0011264	0.0004859	0.017	outcome	TRUE	reported	FVRUio
334	rs7012866	8	135616959	T	G	0.492259	0.0011334	0.0004713	0.015	outcome	TRUE	reported	FVRUio
392	rs7847431	13	73826901	A	G	0.955908	0.0011466	0.0011452	0.32	outcome	TRUE	reported	FVRUio
290	rs604723	11	100610546	T	C	0.275115	0.0011837	0.0005316	0.021	outcome	TRUE	reported	FVRUio
414	rs8904	14	35871217	G	A	0.630679	0.0011849	0.0004872	0.015	outcome	TRUE	reported	FVRUio
233	rs365990	14	23861811	A	G	0.626643	0.0011962	0.0004864	0.013	outcome	TRUE	reported	FVRUio
238	rs3807925	7	18543250	A	G	0.653141	0.0011964	0.0004957	0.012	outcome	TRUE	reported	FVRUio
119	rs1481405	22	50228044	C	T	0.914278	0.0012179	0.0008413	0.15	outcome	TRUE	reported	FVRUio
375	rs7555924	6	152160286	A	G	0.915932	0.0012442	0.0008468	0.14	outcome	TRUE	reported	FVRUio
400	rs7938477	20	31214944	C	T	0.851674	0.0012517	0.000673	0.059	outcome	TRUE	reported	FVRUio
111	rs1393548	2	242344695	T	C	0.973491	0.0012626	0.0015059	0.39	outcome	TRUE	reported	FVRUio
108	rs1342046	2	37517566	A	G	0.757636	0.0012834	0.0005532	0.022	outcome	TRUE	reported	FVRUio
130	rs1701095	4	86719165	T	C	0.851651	0.0012973	0.0006669	0.05	outcome	TRUE	reported	FVRUio
319	rs6699618	1	11881441	C	G	0.838416	0.0013048	0.0006381	0.035	outcome	TRUE	reported	FVRUio
417	rs9303175	17	1372987	T	G	0.350597	0.0013219	0.0004961	0.0081	outcome	TRUE	reported	FVRUio
289	rs6044468	10	115717311	G	A	0.959858	0.0013454	0.0012115	0.25	outcome	TRUE	reported	FVRUio
220	rs3402599	9	123516572	A	G	0.420351	0.001356	0.0004799	0.0056	outcome	TRUE	reported	FVRUio
311	rs6490019	12	115920472	A	G	0.379766	0.001359	0.0004855	0.0046	outcome	TRUE	reported	FVRUio
60	rs1174644	10	107158054	G	A	0.981492	0.0013603	0.0017456	0.45	outcome	TRUE	reported	FVRUio
113	rs1419583	19	2165383	G	A	0.960002	0.0013848	0.0011991	0.24	outcome	TRUE	reported	FVRUio
403	rs7978281	6	25882678	G	T	0.902797	0.0014855	0.0007931	0.057	outcome	TRUE	reported	FVRUio
120	rs1484010	8	81386066	C	A	0.965174	0.001499	0.0012821	0.25	outcome	TRUE	reported	FVRUio
380	rs7644357	5	96211594	G	C	0.964022	0.0015206	0.0012626	0.2	outcome	TRUE	reported	FVRUio
170	rs2283500	16	4138378	A	C	0.895426	0.001538	0.0007723	0.048	outcome	TRUE	reported	FVRUio
79	rs1247391	2	182987704	G	A	0.790134	0.0016302	0.000578	0.0044	outcome	TRUE	reported	FVRUio
36	rs1114580	9	139520789	A	G	0.41863	0.0016435	0.0004818	0.00072	outcome	TRUE	reported	FVRUio
352	rs7293174	11	69825414	A	G	0.912211	0.0018013	0.000833	0.036	outcome	TRUE	reported	FVRUio
350	rs7278003	21	44966069	T	C	0.438511	0.0018515	0.0004742	9.90E-05	outcome	TRUE	reported	FVRUio
368	rs7453887	11	67976593	G	C	0.945551	0.0019026	0.0010649	0.07	outcome	TRUE	reported	FVRUio
310	rs6452769	5	87389027	G	A	0.789503	0.0019639	0.0005834	0.00087	outcome	TRUE	reported	FVRUio
364	rs7404819	11	2114221	T	C	0.952147	0.0019745	0.0011218	0.074	outcome	TRUE	reported	FVRUio
82	rs1259663	16	2065666	C	T	0.906016	0.0023614	0.0008167	0.0031	outcome	TRUE	reported	FVRUio
33	rs1098040	9	113249071	T	C	0.963007	0.0024066	0.0012562	0.054	outcome	TRUE	reported	FVRUio

Supplementary Figure 1

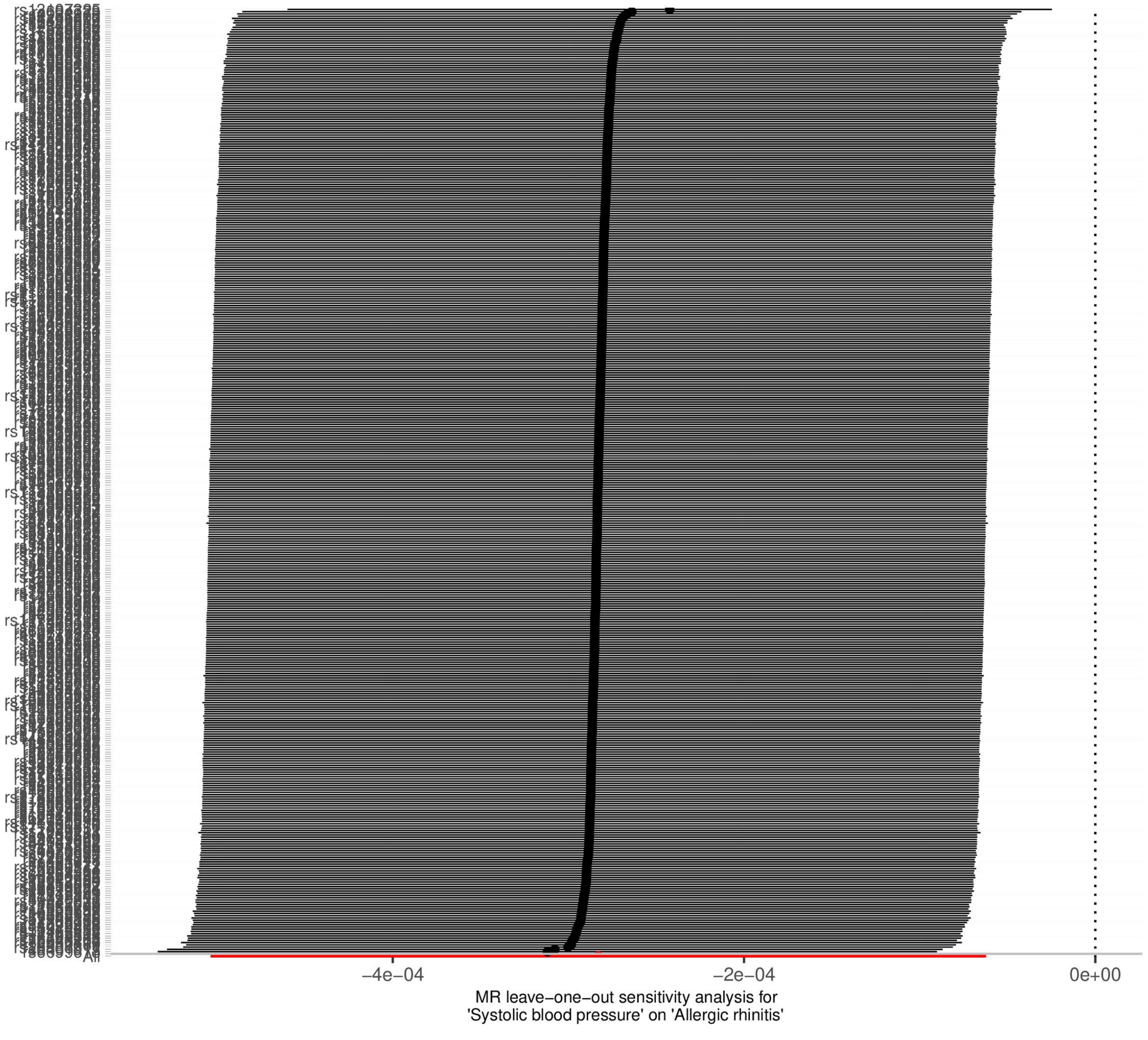

Supplementary Figure 2

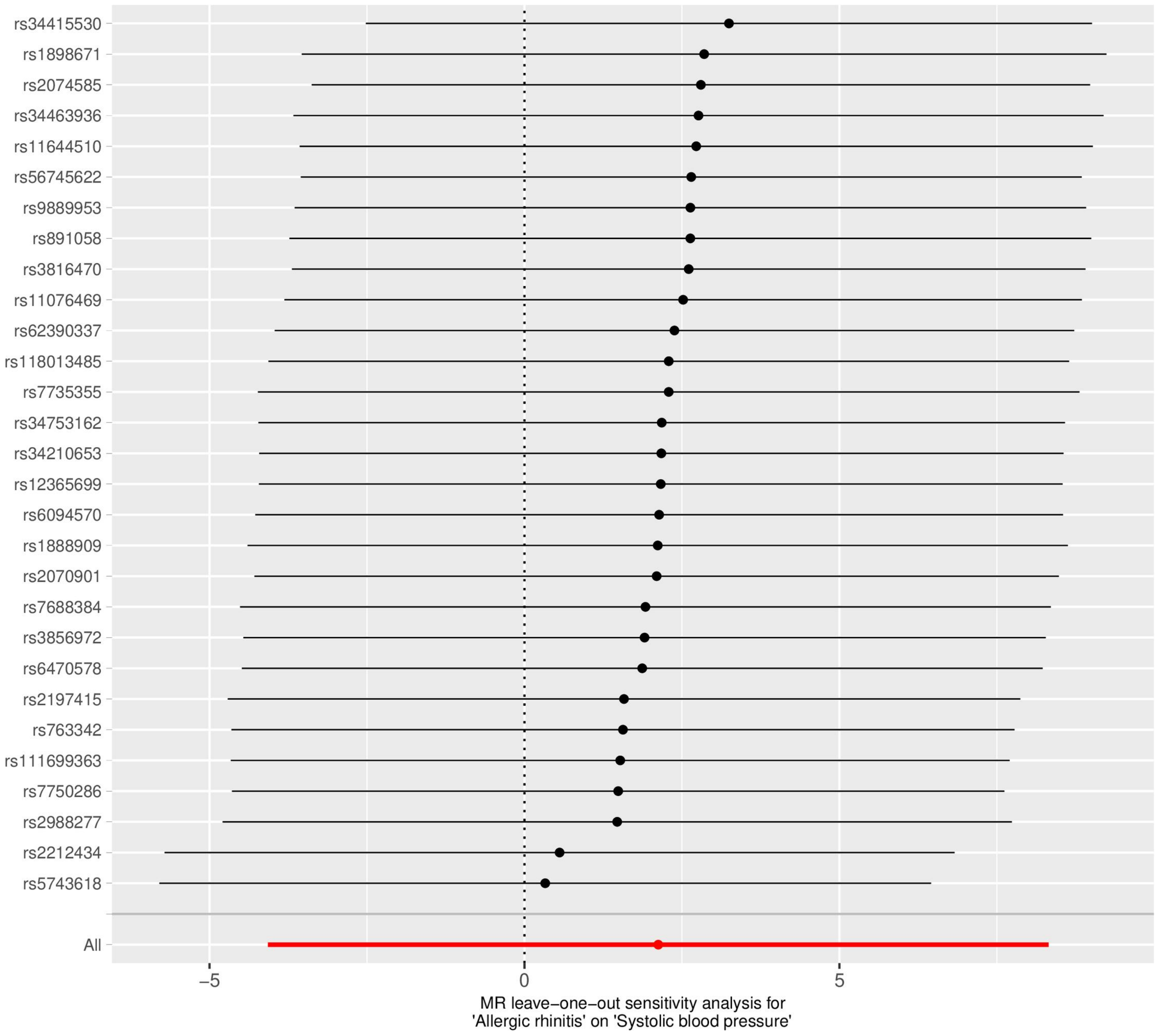

© 2026 Mao Z. et al.
